# Supplementary material for: Iron-borane catalyzed carbonyl hydroboration and isolation of an iron(I)-ketyl radical
Source: Nat Commun. 2026 Feb 19;17:2929. doi: 10.1038/s41467-026-69500-2 (PMC13031794; doi:10.1038/s41467-026-69500-2)
Supplement: Supplementary file 1 — Supplementary Information [file 41467_2026_69500_MOESM1_ESM.pdf]

Supporting Information

Iron-Borane Catalyzed Carbonyl Hydroboration and Isolation of an Iron(I)-Ketyl Radical

Laura A. Grose, Ryan J. Schwamm, Adam Brookfield, David Robinson* and Darren Willcox*

Department of Chemistry, University of Manchester, Oxford Road, M13 9PL

*darren.willcox@manchester.ac.uk

* david.robinson@ntu.ac.uk

Table of Contents

Experimental Procedures 3

Table of Optimizations 4

General procedure 1: hydroboration of ketones 4

General procedure 2: hydroboration of cyclic esters 4

General Procedure 3: hydroboration of carbon dioxide 4

Characterization data of hydroborated products of ketones 5

Characterization data of hydroborated products of cyclic esters 11

CO_2 experiments 13

Mechanistic studies 14

Synthesis of [(^{iPr}DPB^{Ph})Fe(OC(Ph)$_2$)] (9) 14

Infrared spectroscopy 14

UV-Vis spectroscopy 15

General crystallographic methods 15

X-ray diffraction experimental parameters for complex 9 15

Determination of tau (τ) 17

Solution magnetic susceptibility measurements 17

Kinetic experiments on ketones 18

Pre-catalyst rate order assessment 18

HBpin rate order assessment 19

Kinetic isotope effect determination 20

SQUID data 21

Computational details (cyclohexylphenyl ketone mechanism) 25

NMR spectra of compounds 29

References 74

Experimental Procedures

All commercially purchased starting materials were used as received unless otherwise stated. All manipulations were performed using standard Schlenk techniques or an Mbraun glovebox, under an atmosphere of dry N_2. Dry solvents (THF, Et_2O, Pentane, C_6H_6, CH_3CN, toluene and CH_2Cl_2) were obtained using innovative technologies anhydrous engineering solvent purification systems, subsequently degassed and left over 3 Å activated sieves before being transferred to a potassium mirror, except for CH_2Cl_2 and THF. All other solvents used were of HPLC grade, unless otherwise stated. Solvents removed under "reduced pressure" were by rotary evaporation and "*in vacuo*" under high vacuum via Schlenk line. THF-d_8, C_6D_6 and $CDCl_3$ were dried over activated 3 Å molecular sieves and degassed by sparging with dry N_2. All glassware and stirrer bars were flame dried with a blowtorch under a vacuum before use.

^{1}H, ^{11}B, $^{13}C\{^{1}H\}$ and $^{31}P\{^{1}H\}$ NMR spectra were recorded on a Bruker advance III HD 400 spectrometer (operating frequencies: 399.78 MHz, 128.25 MHz, 100.53 MHz and 161.83 ppm, respectively) for reaction monitoring and product characterization. ^{1}H NMR spectra for kinetic experiments was recorded on a Bruker 700 MHz, 5mm BBO prodigy, He-cooled cryoprobe. ^{1}H and $^{13}C\{^{1}H\}$ NMR chemical shifts were internally referenced to the residual solvent resonances ($CDCl_3$ (Chloroform-d): ^{1}H δ = 7.26 ppm, $^{13}C\{^{1}H\}$ δ = 77.16 ppm; C_6D_6 (benzene-d_6): ^{1}H δ = 7.16 ppm, $^{13}C\{^{1}H\}$ δ = 128.02 ppm), THF-d_8 (Tetrahydrofuran-d_8): ^{1}H δ = 3.58, 1.73 ppm, $^{13}C\{^{1}H\}$ δ = 67.57, 25.37 ppm). NMR samples were prepared under an inert atmosphere in 5 mm J. Youngs NMR tubes. Data was analyzed using MestReNova V14.0.0 software. ATR-IR spectra were recorded as microcrystalline powders using a Bruker Tensor 27 spectrometer.

SQUID magnetometry was carried out on a Quantum Design MPMS3 with an Evercool closed cycle helium recirculator. Measurements were carried out in DC mode with eicosane fixed powders loaded into flame sealed NMR tubes. Background corrections for the diamagnetism of the NMR tube/Straw/eicosane were taken into account.

Low temperature EPR measurements were carried out on a Bruker EMXPlus Spectrometer equipped with a Bruker ER4122SHQ resonator. Samples were loaded under vacuum into flame sealed clear fused quartz EPR tubes of 4mm outer diameter. Sample cooling was achieved using a ColdEdge Stinger mated to an Oxford Instruments ESR900 cryostat. Temperature control was managed by an Oxford Instruments MercuryITC.

Table of Optimizations

Table S1: Optimized conditions.

Entry	Fe (mol%)	HBpin Equiv	Solvent	Temp (°C)	Time (h)	Yield (%)[a]
1	0	1.1	C_6D_6	r.t	96	23
2	2	1.1	C_6D_6	r.t	5	99
3	1	1.1	C_6D_6	r.t	5	99
4	0.5	1.1	C_6D_6	r.t	24	49
5	1	1.1	Neat	r.t	24	11
6	1	1.1	Et_2O	r.t	5	89
7	1	1.1	Toluene	r.t	5	95
8	1	1.1	THF	r.t	5	91
9	1	1.1	DCM	r.t	5	0
10	1	1.0	C_6D_6	r.t	5	98
11	1	1.2	C_6D_6	r.t	5	96
12	1	1.5	C_6D_6	r.t	5	96

[a] Yields are given as NMR spectroscopic yields using trimethoxybenzene as internal standard.

General procedure 1: hydroboration of ketones

In a nitrogen filled glovebox, an oven dried J-Youngs NMR tube was charged with $[\{(^{iPr}DPB^{Ph})Fe\}_2(\mu\text{-}1,2\text{-}N_2)]$ (0.00206 mmol), C_6D_6 (0.6 mL), substrate (0.205 mmol), HBpin (0.225 mmol), followed by toluene (0.205 mmol) as internal standard for NMR quantification. The reaction mixture was left at R.T. until reaction was complete (0.1-24 h), monitored by ^1H NMR spectroscopy. Volatiles were removed *in vacuo*, the mixture was suspended in Et_2O and filtered through a short plug of Celite® in a glove box, and volatiles were removed *in vacuo* to reveal isolated product.

General procedure 2: hydroboration of cyclic esters

In a nitrogen filled glovebox, an oven dried J-Youngs NMR tube was charged with $[\{(^{iPr}DPB^{Ph})Fe\}_2(\mu\text{-}1,2\text{-}N_2)]$ (0.005 mmol), C_6D_6 (0.6 mL), substrate (0.103 mmol), HBpin (0.225 mmol), followed by toluene (0.103 mmol) as internal standard for NMR quantification. The reaction mixture was either left at R.T. or added to an oil bath (50 °C) for 4-48 h and monitored by ^1H NMR spectroscopy. Volatiles were removed *in vacuo*, the mixture was suspended in Et_2O and filtered through a short plug of Celite® in a glove box, and volatiles were removed *in vacuo* to reveal isolated product.

General Procedure 3: hydroboration of carbon dioxide

In a nitrogen filled glovebox, desired amount of $[\{(^{iPr}DPB^{Ph})Fe\}_2(\mu\text{-}1,2\text{-}N_2)]$ 2.5 mol% - 1 mol% was dissolved in 0.6 mL of C_6D_6 with HBR_2 (0.205 mmol) and hexamethylbenzene as an

internal standard for NMR quantification in a J-Youngs NMR tube. The NMR tube was taken out of the glovebox and degassed by freeze-pump-thaw, then backfilled with CO_2 (1 bar) from a cylinder. Yields were calculated by ^{1}H NMR spectroscopy.

Characterization data of hydroborated products of ketones

Spectroscopic data is in accordance to literature.[1-3]

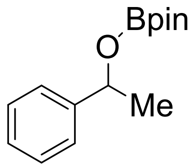

2a, 4,4,5,5-tetramethyl-2-(1-phenylethoxy)-1,3,2-dioxaborolane was prepared according to general procedure 1 using acetophenone (25 µL, 0.205 mmol) and HBpin (32 µL, 0.225 mmol) at R.T. for 0.1 h to furnish product **2a** (49 mg, 0.197 mmol, 96%). **^{1}H NMR** (400 MHz, $CDCl_3$) δ 7.32 – 7.21 (m, 4H), 7.19 – 7.13 (m, 1H), 5.17 (q, *J* = 6.5 Hz, 1H), 1.42 (d, *J* = 6.5 Hz, 3H), 1.17 (s, 6H), 1.14 (s, 6H). **^{13}C{^{1}H} NMR** (101 MHz, $CDCl_3$) δ 144.7, 128.3, 127.2, 125.5, 82.9, 72.7, 25.6, 24.7, 24.7 ppm. **^{11}B NMR** (128 MHz, $CDCl_3$) δ 22.1 ppm.

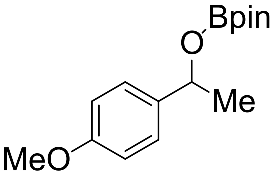

2b, 2-(1-(4-methoxyphenyl)ethoxy)-4,4,5,5-tetramethyl-1,3,2-dioxaborolane was prepared according to general procedure 1 using 4-methoxyphenone (31 µL, 0.205 mmol) and HBpin (32 µL, 0.225 mmol) at R.T. for 0.1 h to furnish product **2b** (53 mg, 0.193 mmol, 94%). **^{1}H NMR** (400 MHz, C_6D_6) δ 7.30 (d, *J* = 8.6 Hz, 2H), 6.76 (d, *J* = 8.7 Hz, 2H), 5.41 (q, *J* = 6.4 Hz, 1H), 3.30 (s, 3H), 1.49 (d, *J* = 6.4 Hz, 3H), 1.04 (s, 6H), 1.02 (s, 6H). **^{13}C NMR** (101 MHz, C_6D_6) δ 159.4, 137.5, 126.9, 114.0, 82.5, 72.7, 54.8, 25.8, 24.7, 24.6. **^{11}B NMR** (128 MHz, C_6D_6) δ 22.6.

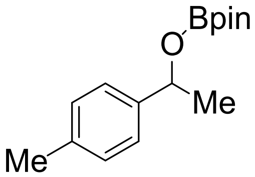

2c, 4,4,5,5-tetramethyl-2-(1-(p-tolyl)ethoxy)-1,3,2-dioxaborolane was prepared according to general procedure 1 using 4-methylacetophenone (28 µL, 0.205 mmol) and HBpin (32 µL, 0.225 mmol) at R.T. for 0.1 h to furnish product **2c** (52 mg, 0.199 mmol, 97%). **^{1}H NMR** (400 MHz, C_6D_6) δ 7.31 (d, *J* = 8.0 Hz, 2H), 6.97 (d, *J* = 7.9 Hz, 2H), 5.43 (q, *J* = 6.4 Hz, 1H), 2.09 (s,

3H), 1.48 (d, *J* = 6.4 Hz, 3H), 1.03 (s, 6H), 1.01 (s, 6H). **^{13}C{^{1}H} NMR** (101 MHz, C_6D_6) δ 142.5, 136.6, 129.2, 125.7, 82.5, 72.9, 25.8, 24.7, 24.6, 21.1. **^{11}B NMR** (128 MHz, C_6D_6) δ 22.6.

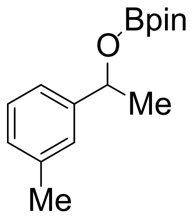

2d, 4,4,5,5-tetramethyl-2-(1-(m-tolyl)ethoxy)-1,3,2-dioxaborolane was prepared according to general procedure 1 using 3-methylacetophenone (28 µL, 0.205 mmol) and HBpin (32 µL, 0.225 mmol) at R.T. for 0.1 h to furnish product **2d** (49 mg, 0.189 mmol, 92%). **^{1}H NMR** (400 MHz, $CDCl_3$) δ 7.24 – 7.18 (m, 2H), 7.18 – 7.13 (m, 1H), 7.09 – 7.03 (m, 1H), 5.23 (q, *J* = 6.4 Hz, 1H), 2.35 (s, 3H), 1.49 (d, *J* = 6.5 Hz, 3H), 1.26 (s, 6H), 1.23 (s, 6H). **^{13}C NMR** (101 MHz, $CDCl_3$) δ 144.6, 137.8, 128.2, 127.9, 126.1, 122.5, 82.8, 72.6, 25.6, 24.6, 21.5. **^{11}B NMR** (128 MHz, $CDCl_3$) δ 22.1.

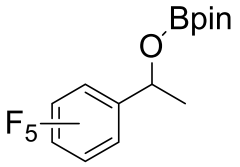

2e, 4,4,5,5-tetramethyl-2-(1-(perfluorophenyl)ethoxy)-1,3,2-dioxaborolane was prepared according to general procedure 1 using 1-(perfluorophenyl)ethenone (38 µL, 0.205 mmol) and HBpin (32 µL, 0.225 mmol) at R.T. for 0.1 h to furnish product **2e** (62 mg, 0.183 mmol, 89%). **^{1}H NMR** (400 MHz, $CDCl_3$) δ 5.58 (q, *J* = 6.7 Hz, 1H), 1.62 (d, *J* = 6.7 Hz, 3H), 1.24 (s, 6H), 1.21 (s, 6H). **^{13}C{^{1}H} NMR** (101 MHz, $CDCl_3$) δ 83.4, 64.3, 24.7 (d, *J* = 12.2 Hz), 22.3 ppm. **^{11}B NMR** (128 MHz, $CDCl_3$) δ 22.1 ppm.

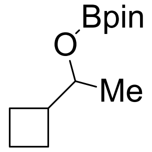

2f, 2-(1-cyclobutylethoxy)-4,4,5,5-tetramethyl-1,3,2-dioxaborolane was prepared according to general procedure 1 using but 3-yn-2-one (16 µL, 0.205 mmol) and HBpin (32 µL, 0.225 mmol) at R.T. for 0.1 h to furnish product **2f** (52 mg, 0.194 mmol, 95%). **^{1}H NMR** (400 MHz, $CDCl_3$) δ 4.07 (p, *J* = 6.3 Hz, 1H), 2.32 (h, *J* = 8.4 Hz, 1H), 2.00 – 1.63 (m, 6H), 1.24 (s, 12H), 1.06

(d, J = 6.2 Hz, 3H). **$^{13}C\{^1H\}$ NMR** (101 MHz, $CDCl_3$) δ 82.6, 74.4, 42.1, 24.7, 24.6, 24.2, 19.7, 17.8 ppm. **^{11}B NMR** (128 MHz, $CDCl_3$) δ 22.0 ppm.

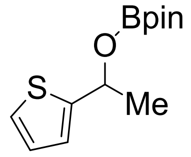

2g, 4,4,5,5-tetramethyl-2-(1-(thiophen-2-yl)ethoxy)-1,3,2-dioxaborolane was prepared according to general procedure 1 using (1-thiophen-2-yl)ethan-1-one (26 mg, 0.205 mmol) and HBpin (32 µL, 0.225 mmol) at R.T. for 0.1 h to furnish product **2g** (50 mg, 0.197 mmol, 96%). **^1H NMR** (500 MHz, C_6D_6) δ 6.85 (dt, J = 3.5, 1.1 Hz, 1H), 6.82 (dd, J = 5.1, 1.3 Hz, 1H), 6.69 (dd, J = 5.1, 3.5 Hz, 1H), 5.64 (q, J = 6.4 Hz, 1H), 1.52 (d, J = 6.5 Hz, 3H), 1.03 (s, 12H). **$^{13}C\{^1H\}$ NMR** (126 MHz, C_6D_6) δ 149.0, 126.6, 124.3, 123.5, 82.7, 69.1, 25.4, 24.7, 24.6. **^{11}B NMR** (128 MHz, C_6D_6) δ 22.1 ppm.

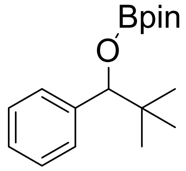

2h, 2-(2,2-dimethyl-1-phenylpropoxy)-4,4,5,5-tetramethyl-1,3,2-dioxaborolane was prepared according to general procedure 1 using 2,2,2-trimethylacetophenone (34 µL, 0.205 mmol) and HBpin (32 µL, 0.225 mmol) at R.T. for 0.5 h to furnish product **2h** (47 mg, 0.162 mmol, 79%). **^1H NMR** (400 MHz, $CDCl_3$) δ 7.28 – 7.12 (m, 5H), 4.69 (s, 1H), 1.13 (s, 6H), 1.08 (s, 6H), 0.83 (s, 9H). **$^{13}C\{^1H\}$ NMR** (101 MHz, $CDCl_3$) δ 141.0, 127.9, 127.3, 84.1, 82.7, 36.0, 26.0, 24.6, 24.5 ppm. **^{11}B NMR** (128 MHz, $CDCl_3$) δ 22.1 ppm.

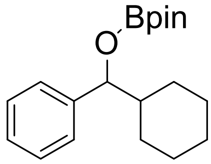

2i, 2-(cyclohexyl(phenyl)methoxy)-4,4,5,5-tetramethyl-1,3,2-dioxaborolane was prepared according to general procedure 1 using cyclohexylphenylketone (39 µL, 0.205 mmol) and HBpin (32 mg, 0.225 mmol) at R.T. for 5 h to furnish product **2i** (64 mg, 0.203 mmol, 99%). **^1H NMR** (400 MHz, $CDCl_3$) δ 7.25 (d, J = 4.4 Hz, 4H), 7.19 (dtd, J = 8.8, 4.3, 2.4 Hz, 1H), 4.74 (d, J = 6.8 Hz, 1H), 1.83 – 1.50 (m, 5H), 1.39 (ddd, J = 12.8, 3.5, 2.0 Hz, 1H), 1.17 (s, 6H), 1.13 (s, 6H), 1.10 – 0.99 (m, 3H), 0.92 (qd, J = 12.5, 3.6 Hz, 1H). **$^{13}C\{^1H\}$ NMR** (101 MHz, $CDCl_3$) δ 142.5, 127.9, 127.1, 126.7, 82.7, 81.1, 45.0, 29.2, 28.2, 26.5, 26.2, 26.1, 24.6 ppm. **^{11}B NMR** (128 MHz, $CDCl_3$) δ 22.2 ppm.

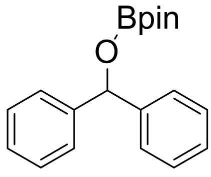

2j, 2-(benzhydryloxy)-4,4,5,5-tetramethyl-1,3,2-dioxaborolane was prepared according to general procedure 1 using benzophenone (37 mg, 0.205 mmol) and HBpin (32 µL, 0.225 mmol) at R.T. for 0.1 h to furnish product **2j** (62 mg, 0.199 mmol, 97%). **^{1}H NMR** (400 MHz, $CDCl_3$) δ 7.48 – 7.41 (m, 4H), 7.40 – 7.23 (m, 6H), 6.24 (s, 1H), 1.26 (s, 12H). **^{13}C{^{1}H} NMR** (101 MHz, $CDCl_3$) δ 143.2, 128.3, 127.4, 126.6, 83.1, 78.0, 24.6 ppm. **^{11}B NMR** (128 MHz, $CDCl_3$) δ 22.5 ppm.

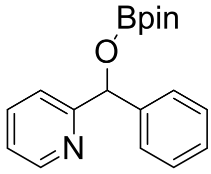

2k, 2-(phenyl((4,4,5,5-tetramethyl-1,3,2-dioxaborolan-2yl)oxy)methyl)pyridine was prepared according to general procedure 1 using 2-benzoylpyridine (38 µL, 0.205 mmol) and HBpin (32 mg, 0.225 mmol) at R.T. for 0.1 h to furnish product **2k** (61 mg, 0.196 mmol, 96%). **^{1}H NMR** (400 MHz, $CDCl_3$) δ 8.56 (dt, *J* = 5.6, 1.3 Hz, 1H), 7.83 (td, *J* = 7.7, 1.5 Hz, 1H), 7.48 – 7.36 (m, 3H), 7.32 – 7.16 (m, 4H), 6.04 (s, 1H), 1.30 (s, 12H). **^{13}C{^{1}H} NMR** (101 MHz, $CDCl_3$) δ 162.1, 141.3, 141.2, 140.9, 128.7, 128.2, 127.0, 124.1, 121.2, 80.2, 78.2, 26.0, 25.8 ppm. **^{11}B NMR** (128 MHz, $CDCl_3$) δ 12.3 ppm.

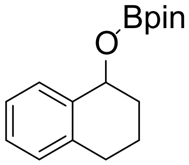

2l, 4,4,5,5-tetramethyl-2-((1,2,3,4-tetrahydronaphthalen-1-yl)oxy)-1,3,2-dioxaborolane was prepared according to general procedure 1 using 3,4-dihydronaphtalen-1(2H)-one (30 mg, 0.205 mmol) and HBpin (32 µL, 0.225 mmol) at R.T. for 0.5 h to furnish product **2l** (51 mg, 0.186 mmol, 91%). **^{1}H NMR** (400 MHz, $CDCl_3$) δ 7.42 – 7.37 (m, 1H), 7.22 – 7.13 (m, 2H), 7.12 – 7.05 (m, 1H), 5.21 (t, *J* = 5.0 Hz, 1H), 2.84 (dt, *J* = 16.8, 5.7 Hz, 1H), 2.71 (ddd, *J* = 16.8, 8.6, 5.6 Hz, 1H), 2.12 – 1.91 (m, 3H), 1.77 (ddd, *J* = 12.4, 10.2, 5.8 Hz, 1H), 1.31 (s, 6H), 1.30 (s, 6H). **^{13}C{^{1}H} NMR** (101 MHz, $CDCl_3$) δ 137.6, 137.3, 128.9, 128.9, 127.5, 125.9, 82.9, 70.6, 31.4, 29.2, 24.8, 24.7, 18.8 ppm. **^{11}B NMR** (128 MHz, $CDCl_3$) δ 22.3 ppm.

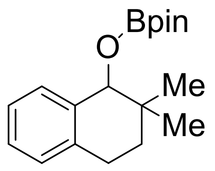

2m, 2-((2,2-dimethyl-1,2,3,4-tetrahydronaphthalen-1-yl)oxy)-4,4,5,5-tetramethyl-1,3,2-dioxaborolane was prepared according to general procedure 1 using 2-dimethyl-3,4-dihydronaphthalene-1-one (36 mg, 0.205 mmol) and HBpin (32 µL, 0.225 mmol) at R.T. for 24 h to furnish product **2m** (28 mg, 0.126 mmol, 61%). **^{1}H NMR** (500 MHz, C_6D_6) δ 7.64 (d, *J* = 7.5 Hz, 1H), 7.13 (t, *J* = 7.7 Hz, 1H), 7.09 (t, *J* = 7.4 Hz, 1H), 6.96 (d, *J* = 7.5 Hz, 1H), 5.01 (s, 1H), 2.77 – 2.39 (m, 3H), 1.89 – 1.83 (m, 1H), 1.32 – 1.37 (m, 1H), 1.11 (s, 6H), 1.09 (s, 6H), 1.07 (s, 3H), 0.89 (s, 3H). **^{13}C{^{1}H} NMR** (126 MHz, C_6D_6) δ 137.9, 136.2, 129.2, 129.0, 127.6, 126.2, 82.6, 79.1, 34.1, 31.9, 26.2, 25.2, 24.7, 24.6, 23.5 ppm. **^{11}B NMR** (128 MHz, C_6D_6) δ 22.4 ppm. **HRMS** (ESI) m/z calcd for [M] 302.22, found $[M+H]^-$ 303.21.

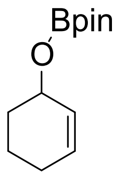

2n, 2-(cyclohex-2-en-1-yloxy)-4,4,5,5-tetramethyl-1,3,2-dioxaborolane was prepared according to general procedure 1 using cyclohexenone (20 µL, 0.205 mmol) and HBpin (32 µL, 0.225 mmol) at R.T. for 24 h to furnish product **2n** (44 mg, 0.196 mmol, 96%). **^{1}H NMR** (400 MHz, $CDCl_3$) δ 5.79 (dtd, *J* = 10.0, 3.6, 1.4 Hz, 1H), 5.74 – 5.63 (m, 1H), 4.54 (dtt, *J* = 4.8, 3.4, 1.5 Hz, 1H), 2.06 – 1.96 (m, 1H), 1.93 (dtt, *J* = 6.4, 4.4, 2.1 Hz, 1H), 1.86 – 1.79 (m, 1H), 1.75 (dddd, *J* = 14.3, 8.4, 5.6, 2.5 Hz, 1H), 1.70 – 1.60 (m, 1H), 1.59 – 1.48 (m, 1H), 1.24 (s, 12H). **^{13}C{^{1}H} NMR** (101 MHz, $CDCl_3$) δ 130.4, 128.9, 82.7, 68.2, 30.9, 25.0, 24.7, 19.1 ppm. **^{11}B NMR** (128 MHz, $CDCl_3$) δ 22.1 ppm.

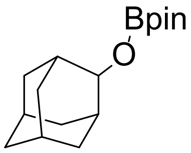

2o, 2-(((1r,3r,5r,7r)-adamantan-2-yl)oxy)-4,4,5,5-tetramethyl-1,3,2-dioxaborolane was prepared according to general procedure 1 using adamantane ketone (33 mg, 0.205 mmol) and HBpin (32 µL, 0.225 mmol) at R.T. for 3 h to furnish product **2o** (52 mg, 0.187 mmol, 91%). **^{1}H NMR** (400 MHz, $CDCl_3$) δ 4.19 (s, 1H), 2.14 (d, *J* = 12.7 Hz, 2H), 1.91 (s, 2H), 1.89 – 1.63 (m, 8H), 1.45 (d, *J* = 12.3 Hz, 2H), 1.25 (s, 12H). **^{13}C NMR** (101 MHz, $CDCl_3$) δ 82.0, 76.6, 37.3, 36.0, 33.6, 30.8, 27.1, 26.7, 24.3. **^{11}B NMR** (128 MHz, $CDCl_3$) δ 22.4.

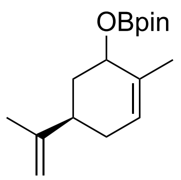

2p, 4,4,5,5-tetramethyl-2-(((5*R*)-2-methyl-5-(prop-1-en-2-yl)cyclohex-2-en-1-yl)oxy)-1,3,2-dioxaborolane was prepared according to general procedure 1 using (*R*)-Carvone (30 µL, 0.205 mmol) and HBpin (32 µL, 0.225 mmol) at R.T. for 0.1 h to furnish product **2p** (54.4 mg, 0.194 mmol, 95%). **^{1}H NMR** (400 MHz, $CDCl_3$) δ 5.46 (d, J = 3.7 Hz, 1H), 4.70 (s, 2H), 4.63 (t, J = 8.6 Hz, 1H), 2.30–2.23 (m, 1H), 2.12–2.07 (m, 1H), 2.02–1.98 (m, 1H), 1.95–1.87 (m, 1H), 1.70 (s, 3H), 1.68 (s, 3H), 1.61–1.52 (m, 1H), 1.25 (s, 12H).**^{13}C{^{1}H} NMR** (101 MHz, $CDCl_3$) δ 149.0, 135.3, 123.9, 109.1, 82.7, 73.6, 40.7, 36.6, 31.1, 24.7, 24.6, 20.4, 18.9 ppm. **^{11}B NMR** (128 MHz, $CDCl_3$) δ 22.3 ppm.

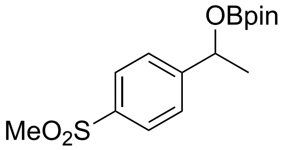

2q, 4,4,5,5-tetramethyl-2-(1-(4-(methylsulfonyl)phenyl)ethoxy)-1,3,2-dioxaborolane was prepared according to general procedure 1 using 4′ -(methylsulfonyl)acetophenone (40.6 mg, 0.205 mmol) and HBpin (32 µL, 0.225 mmol) at R.T. for 0.1 h to furnish product **2q** (50.6 mg, 0.156mmol, 76%). **^{1}H NMR** (400 MHz, $CDCl_3$) δ 7.87 (d, J = 8.0 Hz, 2H), 7.54 (d, J = 7.5 Hz, 2H), 5.29 (q, J = 6.5 Hz, 1H), 3.02 (s, 3H), 1.47 (d, J = 6.5 Hz, 3H), 1.22 (s, 6H), 1.19 (s, 6H).**^{13}C{^{1}H} NMR** (101 MHz, $CDCl_3$) δ 150.8, 139.2, 127.4, 126.2, 83.1, 71.8, 44.6, 25.3, 24.5 ppm. **^{11}B NMR** (128 MHz, $CDCl_3$) δ 22.2 ppm.

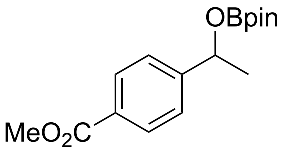

2r, methyl 4-(1-((4,4,5,5-tetramethyl-1,3,2-dioxaborolan-2-yl)oxy)ethyl)benzoate was prepared according to general procedure 1 using methyl 4-acetylbenzoate (36.5 mg, 0.205 mmol) and HBpin (32 µL, 0.225 mmol) at R.T. for 0.1 h to furnish product **2r** (61.0 mg, 0.199 mmol, 97%). **^{1}H NMR** (400 MHz, $CDCl_3$) δ 7.98 (d, J = 8.2 Hz, 2H), 7.41 (d, J = 8.3 Hz, 2H), 5.26 (q, J = 6.5 Hz, 1H), 3.88 (d, J = 1.4 Hz, 3H), 1.47 (d, J = 6.6 Hz, 3H), 1.22 (s, 6H), 1.19 (s, 6H).**^{13}C{^{1}H} NMR** (101 MHz, $CDCl_3$) δ 167.0, 149.7, 129.7, 129.0, 125.3, 83.0, 72.2, 52.0, 25.3, 24.6, 24.5 ppm. **^{11}B NMR** (128 MHz, $CDCl_3$) δ 22.2 ppm.

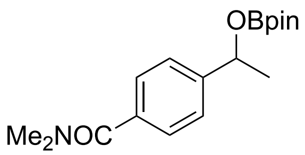

2s, *N*,*N*-dimethyl-4-(1-((4,4,5,5-tetramethyl-1,3,2-dioxaborolan-2-yl)oxy)ethyl)benzamide was prepared according to general procedure 1 using 4-acetyl-*N*,*N*-dimethylbenzamide (39.2 mg, 0.205 mmol) and HBpin (32 µL, 0.225 mmol) at R.T. for 0.1 h to furnish product **2s** (62 mg, 0.195 mmol, 95%). **^{1}H NMR** (400 MHz, $CDCl_3$) δ 7.36 (s, 4H), 5.24 (q, J = 6.4 Hz, 1H), 3.08 (s, 3H), 2.95 (s, 3H), 1.46 (d, J = 6.4 Hz, 3H), 1.22 (s, 6H), 1.20 (s, 6H). **^{13}C{^{1}H} NMR** (101 MHz, $CDCl_3$) δ 171.7, 146.1, 135.2, 127.1, 125.3, 82.9, 72.3, 39.6, 35.4, 25.4, 24.6, 24.6 ppm **^{11}B NMR** (128 MHz, $CDCl_3$) δ 22.2 ppm.

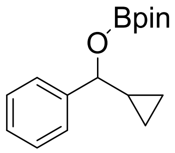

2t, 2-(cyclopropyl(phenyl)methoxy)-4,4,5,5-tetramethyl-1,3,2-dioxaborolane was prepared according to general procedure 1 using benzoylcyclopropane (28 µL, 0.205 mmol) and HBpin (32 µL, 0.225 mmol) at R.T. for 0.1 h to furnish product **2t** (53 mg, 0.193 mmol, 94%). **^{1}H NMR** (400 MHz, C_6D_6) δ 7.46 (d, *J* = 7.8 Hz, 2H), 7.18 – 7.14 (m, 2H), 7.07 (m, 2H), 4.76 (d, *J* = 7.7 Hz, 1H), 1.20 (m, 1H), 1.01 (d, *J* = 10.5 Hz, 12H), 0.70 (m, 1H), 0.41 (m, 1H), 0.31 (m, 2H). **^{13}C NMR** (101 MHz, C_6D_6) δ 143.9, 128.5, 127.5, 126.4, 82.6, 80.2, 24.7, 24.6, 19.8, 3.7, 3.2. **^{11}B NMR** (128 MHz, C_6D_6) δ 22.7.

Characterization data of hydroborated products of cyclic esters

Spectroscopic data is in accordance to literature.[4-5]

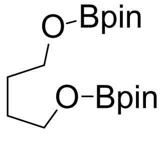

4a, 1,4-bis((4,4,5,5-tetramethyl-1,3,2-dioxaborolan-2-yl)oxy)butane was prepared according to general procedure 2 using γ-butyrolactone (8 mg, 0.103 mmol) and HBpin (32 µL, 0.225 mmol) at R.T. for 12 h to furnish product **4a** (35 mg, 0.102 mmol, 99%). **^{1}H NMR** (400 MHz, $CDCl_3$) δ 3.86 – 3.79 (m, 4H), 1.62 (m, 4H), 1.23 (s, 24H). **^{13}C{^{1}H} NMR** (101 MHz, $CDCl_3$) δ 82.7, 64.6, 27.7, 24.7 ppm. **^{11}B NMR** (128 MHz, $CDCl_3$) δ 22.1 ppm.

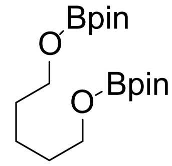

4b, 1,5-bis((4,4,5,5-tetramethyl-1,3,2-dioxaborolan-2-yl)oxy)pentane was prepared according to general procedure 2 using δ-valerolactone (10 µL, 0.103 mmol) and HBpin (32 µL, 0.225 mmol) at R.T. for 24 h to furnish product **4b** (32 mg, 0.090 mmol, 87%). **^{1}H NMR** (400 MHz, $CDCl_3$) δ 3.82 (t, *J* = 6.6 Hz, 4H), 1.60 – 1.54 (m, 4H), 1.39 (m, 2H), 1.24 (s, 24H). **^{13}C{^{1}H} NMR** (101 MHz, $CDCl_3$) δ 82.7, 64.9, 31.2, 24.7, 21.8 ppm. **^{11}B NMR** (128 MHz, $CDCl_3$) δ 22.1 ppm.

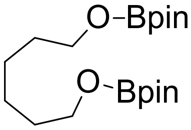

4c, 1,6-bis((4,4,5,5-tetramethyl-1,3,2-dioxaborolan-2-yl)oxy)hexane was prepared according to general procedure 2 using ε-caprolactone (12 µL, 0.103 mmol) and HBpin (32 µL, 0.225 mmol) at R.T. for 4 h to furnish product **4c** (35 mg, 0.095 mmol, 92%).**^{1}H NMR** (400 MHz, $CDCl_3$) δ 3.81 (t, *J* = 6.6 Hz, 4H), 1.59 – 1.49 (m, 4H), 1.37 – 1.31 (m, 4H), 1.23 (s, 24H). **^{13}C{^{1}H} NMR** (101 MHz, $CDCl_3$) δ 82.7, 65.0, 31.5, 25.4, 24.7 ppm. **^{11}B NMR** (128 MHz, $CDCl_3$) δ 22.1 ppm.

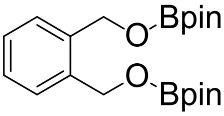

4d, 1,2-bis(((4,4,5,5-tetramethyl-1,3,2-dioxaborolan-2-yl)oxy)methyl)benzene was prepared according to general procedure 2 using phthalide (14 µL, 0.103 mmol) and HBpin (32 µL, 0.225 mmol) at 50 °C for 24 h to furnish product **4d** (35 mg, 0.090 mmol, 87%). **^{1}H NMR** (400 MHz, $CDCl_3$) δ 7.41 (dd, *J* = 5.6, 3.4 Hz, 2H), 7.25 – 7.22 (m, 2H), 4.95 (s, 4H), 1.23 (s, 24H). **^{13}C{^{1}H} NMR** (101 MHz, $CDCl_3$) δ 136.5, 127.6, 127.3, 83.1, 64.1, 24.7 ppm. **^{11}B NMR** (128 MHz, $CDCl_3$) δ 22.3 ppm.

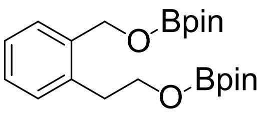

4e, 4,4,5,5-tetramethyl-2-((2-(2-((4,4,5,5-tetramethyl-1,3,2-dioxaborolan-2-yl)oxy)ethyl)benzyl)oxy)-1,3,2-dioxaborolane was prepared according to general procedure 2

using isochroman-1-one (14 µL, 0.103 mmol) and HBpin (32 µL, 0.225 mmol) at 50 °C for 24 h to furnish product **4e** (29 mg, 0.072 mmol, 70%). **^{1}H NMR** (400 MHz, $CDCl_3$) δ 7.46 – 7.34 (m, 1H), 7.23 – 7.15 (m, 3H), 4.98 (s, 2H), 4.03 (t, *J* = 7.2 Hz, 2H), 2.93 (t, *J* = 7.2 Hz, 2H), 1.26 (s, 12H), 1.19 (s, 12H). **^{13}C{^{1}H} NMR** (101 MHz, $CDCl_3$) δ 137.5, 135.9, 130.1, 127.9, 127.6, 126.6, 83.0, 82.8, 65.2, 64.6, 34.4, 24.8 ppm. **^{11}B NMR** (128 MHz, CDCl3) δ 22.2 ppm.

CO_2 experiments

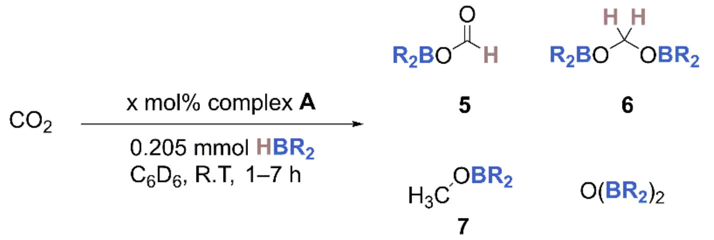

Entry	Borane	mol% A	5	6	7
1	HBpin	2.5	10	14	14
2	HBpin	2.5	9	11	12
3	(9-BBN)$_2$	2.5	-	-	66
4	(9-BBN)$_2$	2.5	-	-	65
5	(9-BBN)$_2$	1	-	-	65
6	(9-BBN)$_2$	1	-	-	62

Mechanistic studies

Synthesis of [(^{iPr}DPB^{Ph})Fe(OC(Ph)$_2$)] (9)

Complex **9** was prepared by dissolving complex **A** (82 mg, 0.150 mmol) and benzophenone (27.2 mg, 0.150 mol) in C_6D_6 (0.6 ml). Immediately the colour changed from dark red to dark purple at R.T, volatiles were removed *in vacuo* affording **9** as a dark solid. Slow evaporation from a concentrated solution of diethyl ether gave dark purple single crystals suitable for XRD (101 mg, 0.141 mmol, 94%). **^{1}H NMR** (400 MHz, C_6D_6) δ 132.20, 126.21, 48.60, 37.76, 35.20, 22.83, 4.96, 2.41, 0.02, –3.78, –5.77, –8.79, –14.25, –29.77, –31.28. ATR-IR (cm^{-1}) 1359 (C–O). Solution magnetic moment (25 °C, C_6D_6) 2.76 μ_B. UV-vis [toluene, λ(nm){ε(M^{-1} cm^{-1})}]: 522(8345), 622(6376).

Infrared spectroscopy

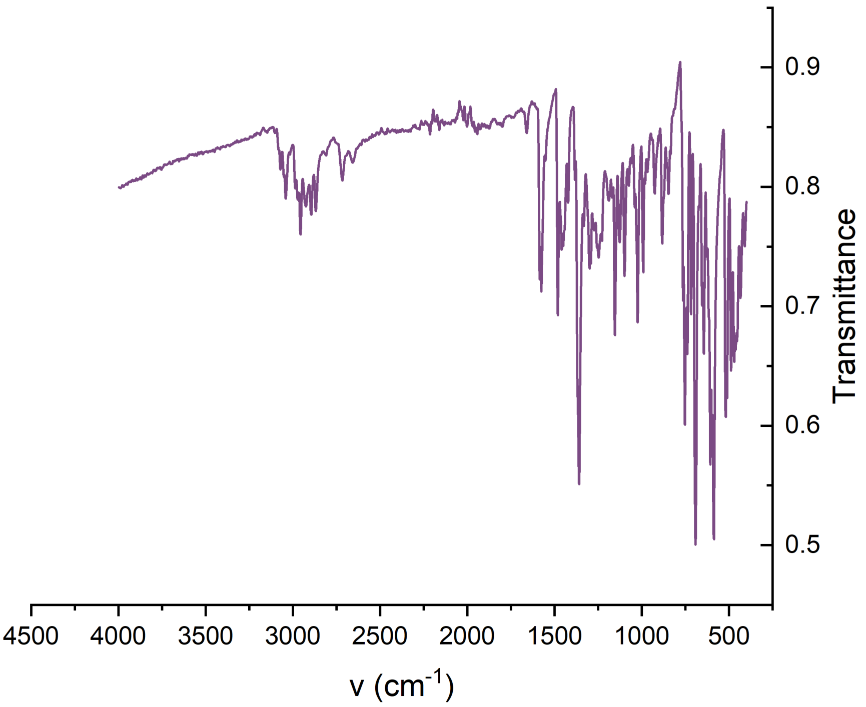

Figure S1 IR spectrum of complex **9**

UV-Vis spectroscopy

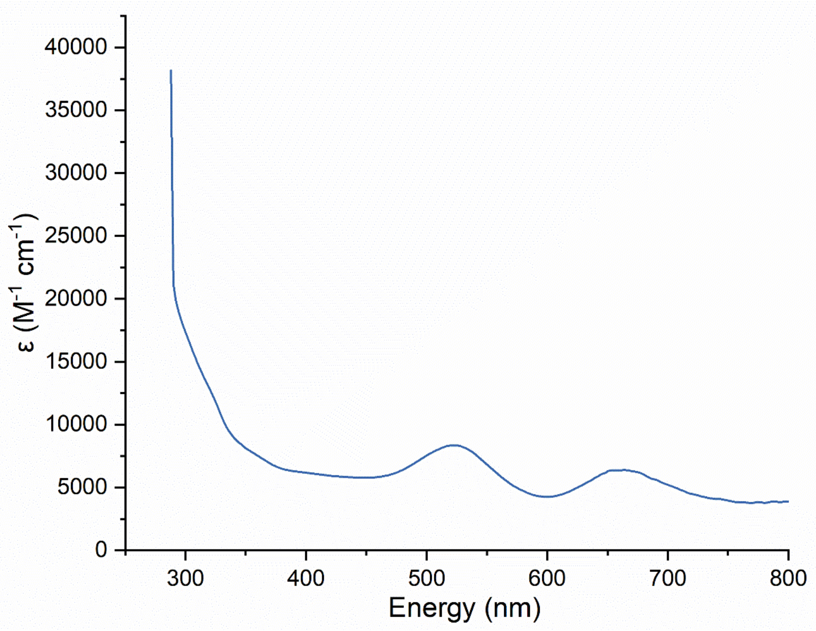

Figure S2 UV-vis spectrum of complex **9** in toluene

General crystallographic methods

The crystal data for complex **9** is recorded in XRD experimental parameters. The crystals were obtained from slow evaporation of diethyl ether. The crystals were examined using an Agilent Supernova diffractometer, equipped with an Eos CCD area detector and a microfocus source with Mo Kα radiation (λ = 0.71073 Å). Intensities were integrated from data recorded on 1° frames by ω rotation. Data was reduced and processed using CrysAlisPro.[6] The structure was solved using olex2.solve[7] structure solution program using Charge Flipping and refined with olex2.refine with anisotropic displacement parameters for all non-hydrogen atoms, and with constrained riding hydrogen geometries; U_{iso}(H) was set at 1.2 (1.5 for methyl groups) times U_{eq} of the parent atom employed through OLEX2 suit program.[8] For molecular graphics ORTEP-3[9] was employed. This data can be obtained free of charge from the Cambridge Crystallographic Data Centre via deposition number 2339051.

X-ray diffraction experimental parameters for complex 9

Empirical formula	$C_{43}H_{51}BFeOP_2$
Formula weight	712.500
Temperature/K	99.9(4)

Crystal system	monoclinic
Space group	C2/c
a/Å	21.4390(8)
b/Å	9.6203(3)
c/Å	37.7813(13)
α/°	90
β/°	104.985(4)
γ/°	90
Volume/Å^3	7527.4(5)
Z	8
ρ_{calc} g/cm^3	1.257
μ/mm^{-1}	0.518
F(000)	3029.9
Crystal size/mm^3	0.223 × 0.201 × 0.041
Radiation	Mo Kα (λ = 0.71073)
2Θ range for data collection/°	5 to 56.52
Index ranges	$-27 \le h \le 23$, $-12 \le k \le 7$, $-34 \le l \le 49$
Reflections collected	15001
Independent reflections	7836 [R_{int} = 0.0416, R_{sigma} = 0.0746]
Data/restraints/parameters	7836/0/441
GOF	1.048
R, $_wR^2$ ($F^2 > 2\sigma(F^2)$)	R_1 = 0.0490, wR_2 = 0.1001
R, $_wR^2$ (all data)	R_1 = 0.0727, wR_2 = 0.1122
Max., min. diff map, e Å^{-3}	0.68/-0.58

[a] Conventional $R = \Sigma||F_o| - |F_c||/\Sigma|F_o|$; $R_w = [\Sigma w(F_o^2 - F_c^2)^2 / \Sigma w(F_o^2)^2]^{1/2}$; $S = [\Sigma w(F_o^2 - F_c^2)^2$ /no. data – no. params)]$^{1/2}$ for all data.

Table 2 Selected bond angles for **9**

Atom	Angle/°
P2–Fe1–P1	119.56(3)
O1–Fe1–P1	112.05(6)
O1–Fe1–P2	103.15(6)
B1–Fe1–P1	80.69(8)
B1–Fe1–P2	81.43(8)
B1–Fe1–O1	160.54(10)
C31–O1–Fe1	172.01(18)

Determination of tau (τ)

$$\tau_4 = \frac{360-(\alpha+\beta)}{141} \qquad (1)$$

β (largest angle) = O1-Fe1-(Fe-B-C)$_{centroid}$ = 140.29 °

α (2nd largest angle) = P1-Fe1-P2 = 119.56 °

τ_4 = 0.71

Solution magnetic susceptibility measurements

Evans' method for solution-state magnetic susceptibility determination was modified and applied to complex **9**.

Equation S1

$$X_M = \frac{3\Delta f}{4\pi F c}$$

Equation S2

$$\mu_{eff} = \sqrt{8(X_M T)}$$

$\Delta f(Hz)$ = change in chemical shift = 344 Hz

F = operating frequency of NMR machine = 400,000 Hz

c = concentration of sample = 0.064380685 Mol/ L

T = operating temperature of NMR = 298 K

Kinetic experiments on ketones

In a nitrogen filled glovebox, an oven dried J-Youngs NMR tube was charged with [{(^{iPr}DPB^{Ph})Fe}$_2$(μ-1,2-N_2)] and cyclohexylphenyl ketone dissolved in C_6D_6 (0.6 mL) followed by HBpin and toluene as internal standard for NMR quantification. The tube was immediately removed from the glovebox and placed into an acetonitrile/liquid nitrogen bath to freeze the solution, the mixture was thawed and shaken to mix the contents before being placed in the spectrometer. Kinetic analysis was performed by monitoring the reaction progress by ^{1}H NMR (700 MHz, C_6D_6) analysis at 60 s intervals over 20 minutes at 298.15 K measured against an internal standard toluene.

Pre-catalyst rate order assessment

Cat A (M)	HBpin (M)	Cyclohexylphenyl ketone (M)	k_{obs}	R^2
0.001603	0.344	0.313	$4.90x10^{-5} \pm 2.5x10^{-7}$	0.9992
0.001985	0.344	0.313	$5.59x10^{-5} \pm 1.2x10^{-7}$	0.9988
0.002366	0.344	0.313	$6.4x10^{-5} \pm 1.0x10^{-6}$	0.9990
0.003893	0.344	0.313	$8.7x10^{-5} \pm 1.3x10^{-6}$	0.9991
0.004656	0.344	0.313	$9.9x10^{-5} \pm 8.6x10^{-7}$	0.9995

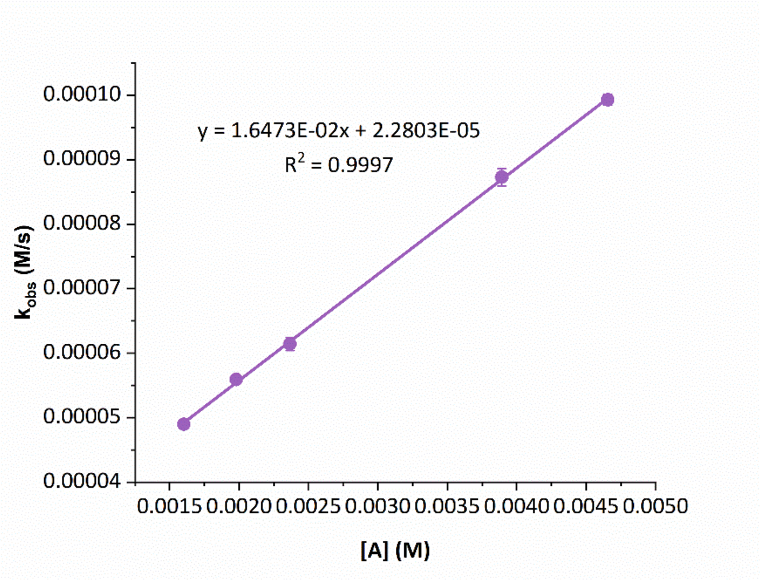

Figure S3 Graph to show concentration of complex **A** *vs.* observed rate equates to a first order dependence on the concentration of **A** over the investigated concentration range.

Ketone rate order assessment

Cat A (M)	HBpin (M)	Cyclohexylphenyl ketone (M)	k_{obs}	R^2
0.00315	0.344	0.032	$1.28x10^{-5} \pm 8.35x10^{-8}$	0.9992
0.00315	0.344	0.056	$2.44x10^{-5} \pm 1.44x10^{-6}$	0.9971
0.00315	0.344	0.084	$3.67x10^{-5} \pm 1.04x10^{-6}$	0.9995
0.00315	0.344	0.122	$5.37x10^{-5} \pm 6.01x10^{-7}$	0.9938
0.00315	0.344	0.160	$6.78x10^{-5} \pm 3.86x10^{-7}$	0.9997
0.00315	0.344	0.237	$6.75x10^{-5} \pm 2.33x10^{-7}$	0.9999
0.00315	0.344	0.389	$6.70x10^{-5} \pm 1.80x10^{-7}$	0.9998
0.00315	0.344	0.466	$6.74x10^{-5} \pm 1.68x10^{-7}$	0.9995

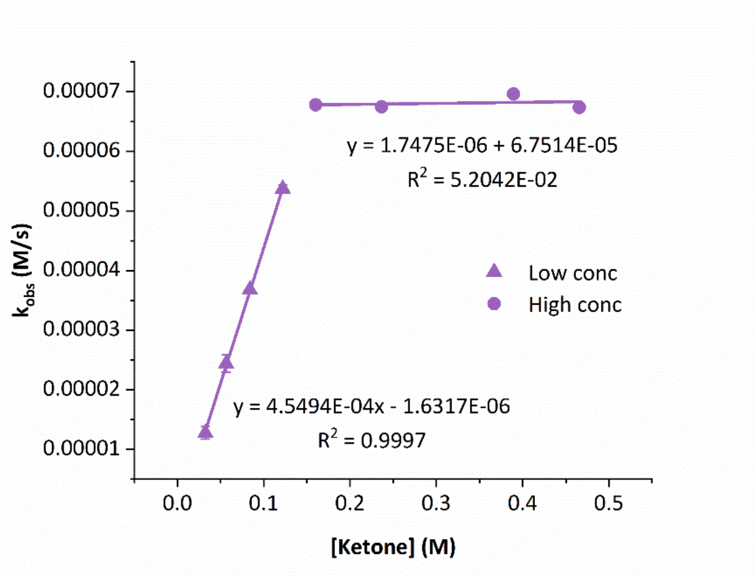

Figure S4 Graph to show concentration of cyclohexylphenyl ketone *vs.* observed rate equates to a zero-order dependence on the concentration of ketone at high concentration and first-order dependence at low concentration.

HBpin rate order assessment

Cat A (M)	HBpin (M)	Cyclohexylphenyl ketone (M)	k_{obs}	R^2
0.00134	0.149	0.205	$5.56x10^{-5} \pm 1.2x10^{-6}$	0.9984
0.00134	0.187	0.205	$5.13x10^{-5} \pm 3.5x10^{-7}$	0.9982
0.00134	0.225	0.205	$4.74x10^{-5} \pm 1.1x10^{-6}$	0.9980
0.00134	0.377	0.205	$3.00x10^{-5} \pm 1.0x10^{-6}$	0.9991
0.00134	0.454	0.205	$2.15x10^{-5} \pm 1.0x10^{-6}$	0.9993

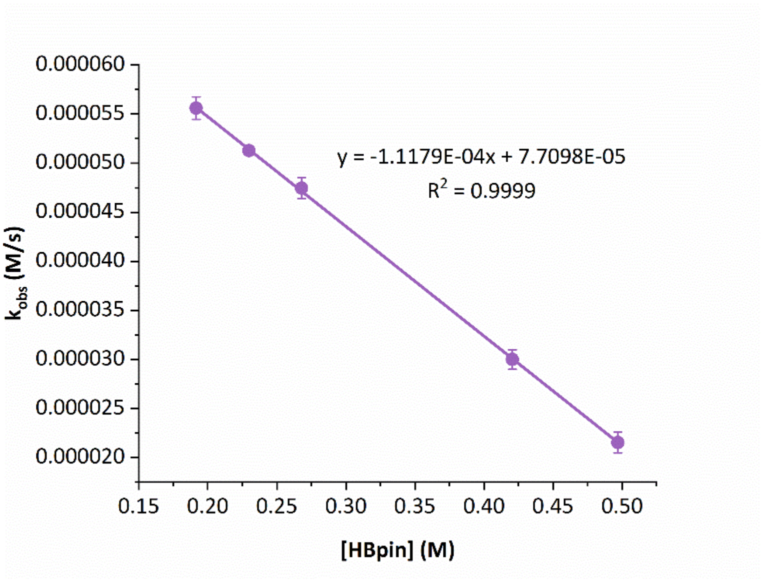

Figure S5 Graph to show concentration of HBpin *vs.* observed rate equates to inverse first-order over investigated concentration range.

Kinetic isotope effect determination

DBpin was synthesized according to literature and used as a known concentration in a solution of C_6D_6.[10]

The KIE was determined using the above procedure for kinetics of ketones with a solution of DBpin in C_6D_6. Data points were collected every 60 seconds over 20 minutes at 298.15 K.

Entry	Cat A (M)	DBpin (M)	Cyclohexylphenyl ketone (M)	k_{obs}	R^2
1	0.00315	0.344	0.313	$3.21x10^{-5}$	0.9959
2	0.00315	0.344	0.313	$3.09x10^{-5}$	0.9764

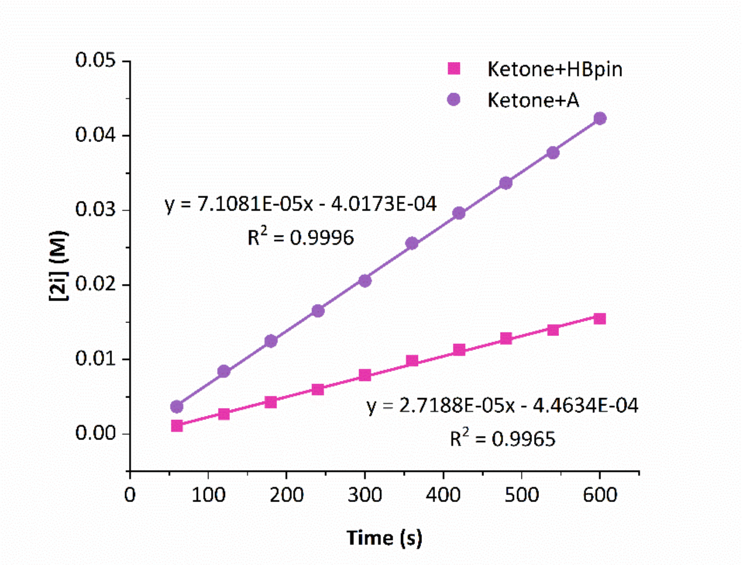

Figure S6 Graph the show the difference in concentration growth with time when series of addition for catalytic experiments is changed. Green – ketone and complex **A** are added together before addition of HBpin. Pink – ketone and HBpin are added together before addition of complex **A**.

SQUID data

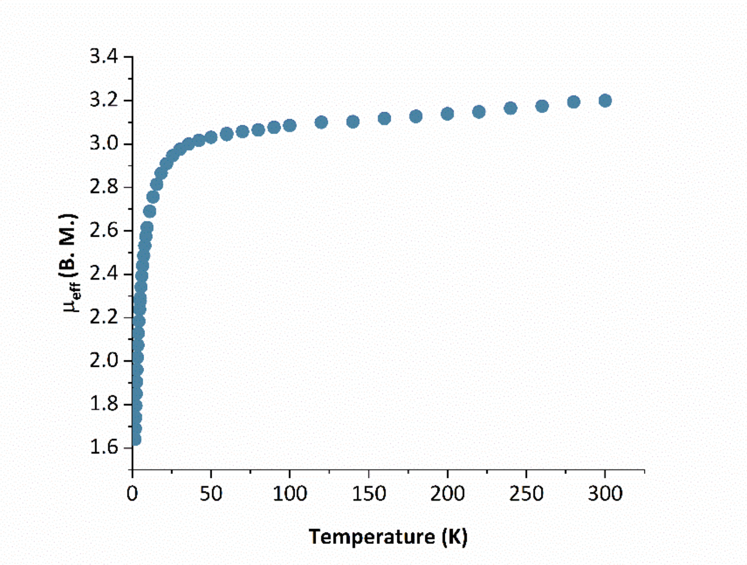

Figure S7 μ_{eff} *vs* Temperature SQUID graph

Computational Details

Initial single-point energy calculations were carried out with the ωB97X-D3 functional[11] and Def2-SVP basis set,[12] followed by stability analysis of the resulting solution for the S=1 spin multiplicity. Once a stable solution had been identified (with $\langle S^2 \rangle$ ~3.25), a geometry optimisation was performed to relax the molecule to a minimum. Further stability analyses were performed to ensure that the SCF solution remained stable. Additionally, we performed calculations where the initial guess was done as a double fragment guess. In these calculations, we performed two different initial guesses: the first with a single unpaired electron each on the ketyl fragment, and the second fragment (with the Fe centre); the second with a single unpaired electron on the ketyl radical and three unpaired electrons on the Fe centre (antiferromagnetically coupled, with S=1). Following convergence of the SCF solutions and stability analysis to the most stable solution, both guesses gave the same SCF solution, with $\langle S^2 \rangle$ ~3.25. The spin density was plotted in IQmol,[13] according to equation [1]:

$$\rho_{spin} = \rho_\alpha - \rho_\beta \qquad [1]$$

The Natural Bond Orbital (NBO, version 5.0) program,[14] as implemented in the Q-Chem 5.4 package,[15] was utilised to assign the nature of the d orbitals with unpaired spin.

The J_{AB} coupling parameter within the Heisenberg-Dirac-van Vleck Hamiltonian is used to characterise the (anti)ferromagnetic coupling strength between two sites with unpaired spins:[16]

$$\hat{H} = -2J_{AB}\vec{S}_A\vec{S}_B \qquad [2]$$

To calculate the (anti)ferromagnetic coupling using broken-spin symmetry methods (in our case, spin-unrestricted DFT), the following three equations can be used:[16]

$$J_{ab}^{(1)} = \frac{^{\mathrm{LS}}E(\mathrm{UDFT}) - {}^{\mathrm{HS}}E(\mathrm{UDFT})}{S_{\max}^2} \qquad [3a]$$

$$J_{ab}^{(2)} = \frac{^{\mathrm{LS}}E(\mathrm{UDFT}) - {}^{\mathrm{HS}}E(\mathrm{UDFT})}{S_{\max}(S_{\max}+1)} \qquad [3b]$$

$$J_{ab}^{(3)} = \frac{^{\mathrm{LS}}E(\mathrm{UDFT}) - {}^{\mathrm{HS}}E(\mathrm{UDFT})}{^{\mathrm{HS}}\langle S^2\rangle - {}^{\mathrm{LS}}\langle S^2\rangle} \qquad [3c]$$

In these approximations, $J_{ab}^{(1)}$ approximately holds when the overlap of the two spin sites' orbitals is small, $J_{ab}^{(2)}$ approximately holds when the overlap is large, while the final parameter, $J_{ab}^{(3)}$, is approximately valid across all coupling scenarios. Negative J_{AB} values correspond to

antiferromagnetic coupling between the two spin sites. Calculations of the high spin (S=2) and low spin (S=1) were performed at the ωB97X-D3/Def2-SVP optimised geometry, with both the same basis set and the higher quality Def2-TZVPP basis set.

All DFT calculations described here were performed with the Q-Chem 5.4 package,[15] except the fragment guess calculations, which were performed with Gaussian 16 (revision A.03).[17]

Time-dependent density functional theory (TDDFT) calculations were performed with the same functional and basis set as given above. The orbital contributions to the two highest intensity peaks from the UV/vis spectrum are highlighted below.

<table>
<tr><th colspan="2">Excited state 1, λ=605 nm (f = 0.0321)</th></tr>
<tr><td>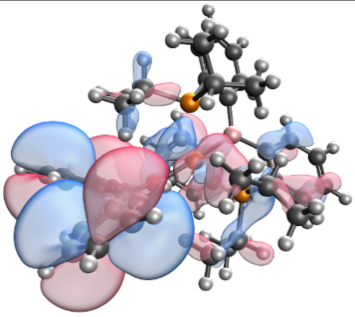
Occupied (MO 187, ketyl π)</td><td>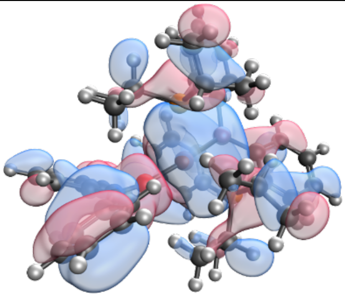
SOMO (mixed Fe d + π*)
Amplitude = 0.73</td></tr>
<tr><th colspan="2">Excited state 2, λ=490 nm (f = 0.0922)</th></tr>
<tr><td rowspan="3">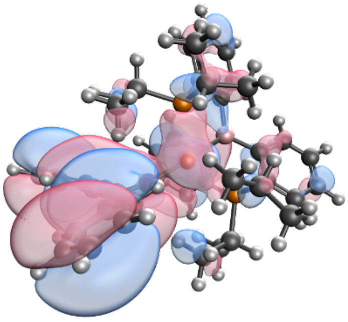
Occupied (MO 188, ketyl π)</td><td>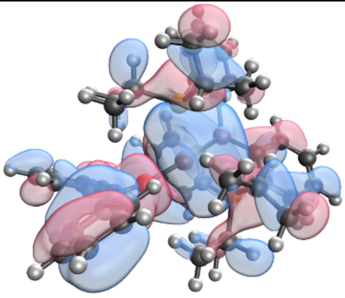
SOMO (MO 189, mixed Fe d + π*)
Amplitude = 0.43</td></tr>
<tr><td>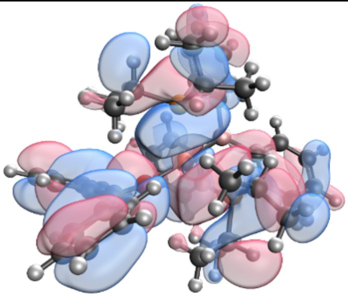
SOMO (MO 190, mixed Fe d + π*)
Amplitude = 0.52</td></tr>
<tr><td>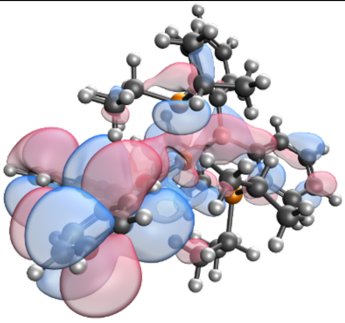
Virtual (MO 192, ketyl π*)
Amplitude = 0.52</td></tr>
</table>

Amplitudes of the excitation vectors from TDDFT calculations of the two peaks shown in the experimental UV/vis spectrum.

The Mulliken spin densities (with a significant contribution) are: Fe 3.20), B(-0.31), C (of the C=O group coordinating to Fe): -0.45. The other carbon atoms of benzophenone have spin densities in the range 0.05 to 0.16.

Computational details (cyclohexylphenyl ketone mechanism)

Geometries of reactants, intermediates, products and transition states were optimised with the r^{2}SCAN-3c functional[18] (which includes a pruned triple-zeta basis set and empirical dispersion) using the Orca 6.0 package.[19] Solvation was included using the conductor-like polarisable continuum model (C-PCM), using the standard Orca parameters for benzene (ε = 2.2790, n = 1.5011 and R_{solv} = 1.3000).[20,21] The nature of the stationary points was confirmed with harmonic vibrational frequency analysis (zero imaginary frequencies for minima, one imaginary frequency for transition states). The final energies were improved using single-point calculations using the ωB97X-D3 functional[11] and def2-TZVP basis set,[12] using C-PCM as described above at the r^{2}SCAN-3c optimised geometries. The final energies used for the reaction profile included the zero-point vibrational energies (ZPE) calculated at the r^{2}SCAN-3c level.

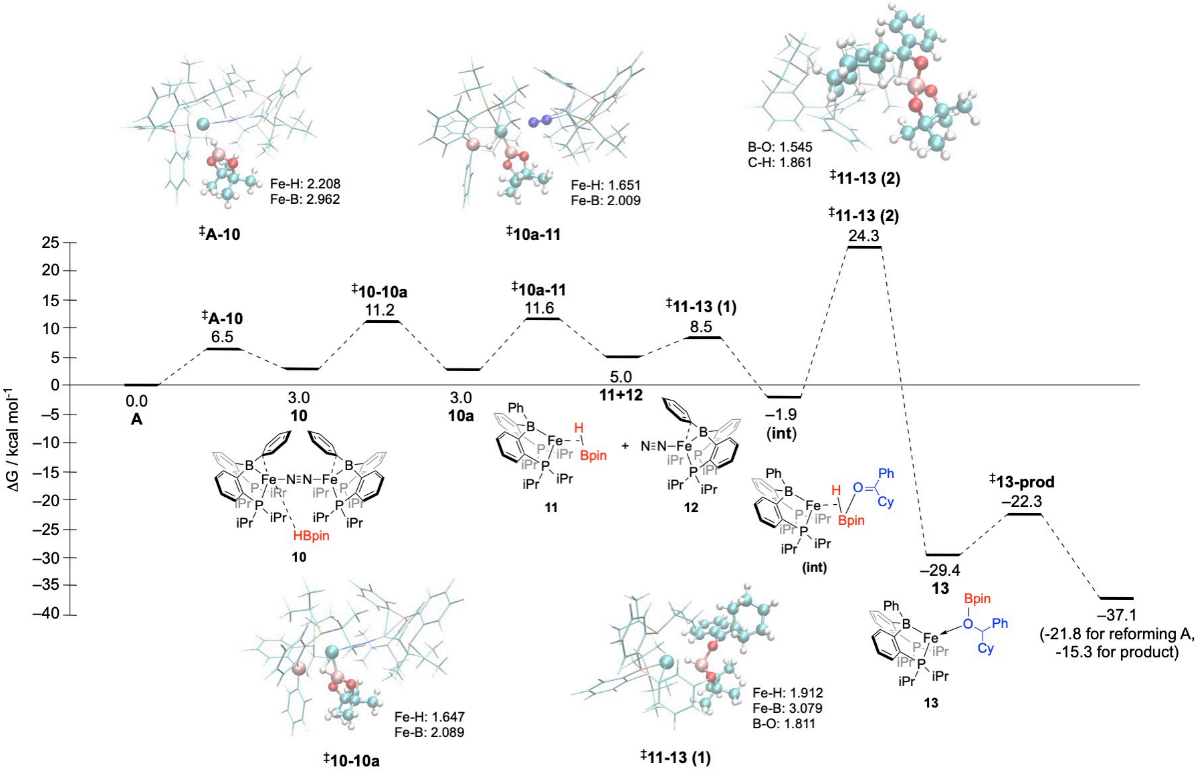

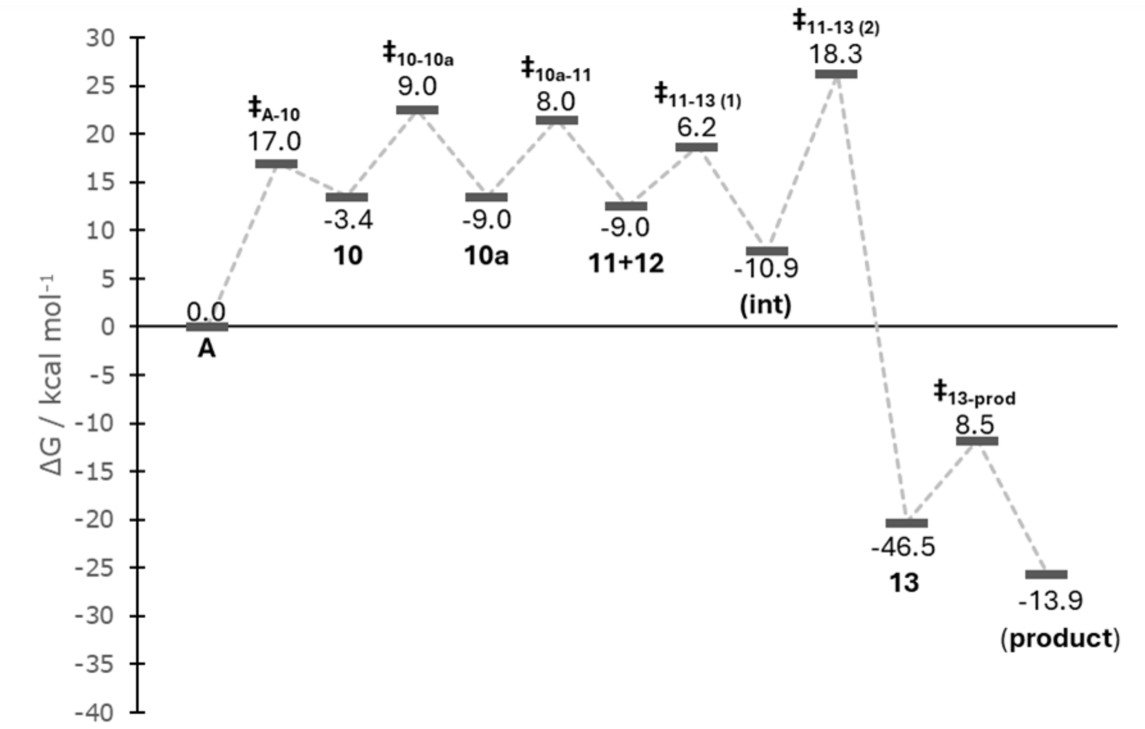

Figure S8. Alternative calculated Gibbs free energy profile. Energies are from ωB97X-D3/def2-TZVP//r^{2}SCAN-3c calculations, with r^{2}SCAN-3c enthalpy and entropy corrections. Values are relative to the previous point. In all cases, the spin multiplicity is S=1 at each Fe centre.

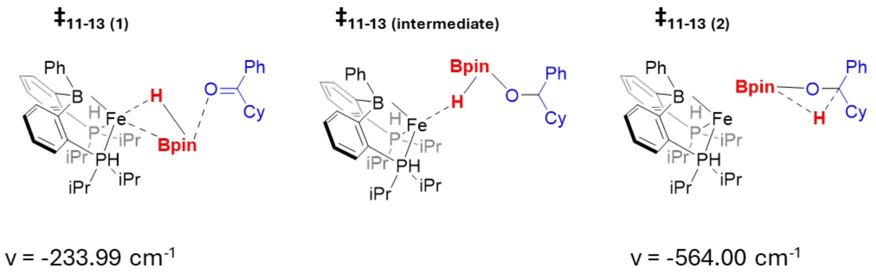

Figure S9. Schematic of the transition state geometries for the LLHT step (rate-limiting step). S=1 for each Fe centre.

Alternative spin multiplicities to S=1 per Fe centre are given in Table S3 (below).

**Molecule**	**E (S=1 per Fe)**	**E (S=0 per Fe)**	**E(S=2 per Fe)**
**A**	-6387.368015	-6387.316636	-6387.262157
**10**	-6799.12918	-6799.089077	-6799.041535
**10a**	-6799.129177	-6799.089074	-6799.094734
**11**	-4130.807416	-4130.786576	-4130.774406
**13**	-4130.856809	-4130.830426	-4130.836008

Table S3. Energies from the optimised geometries for different spin multiplicities of each of the minima reported in the reaction coordinate. Energies are from r^{2}SCAN-3c and are reported in Hartrees.

Alternative pathways were considered (Figure S10). In the alternative to the LLHT step, the (rate-limiting) barrier height was calculated to be 29.1 kcal mol^{-1}, while in the pathway in which the ketone coordinates to the Fe complex first, the rate-limiting barrier height is 31.6 kcal mol^{-1} (top in the alternative pathway, Figure S10) and 38.2 kcal mol^{-1} (bottom in the alternative pathway, Figure S10).

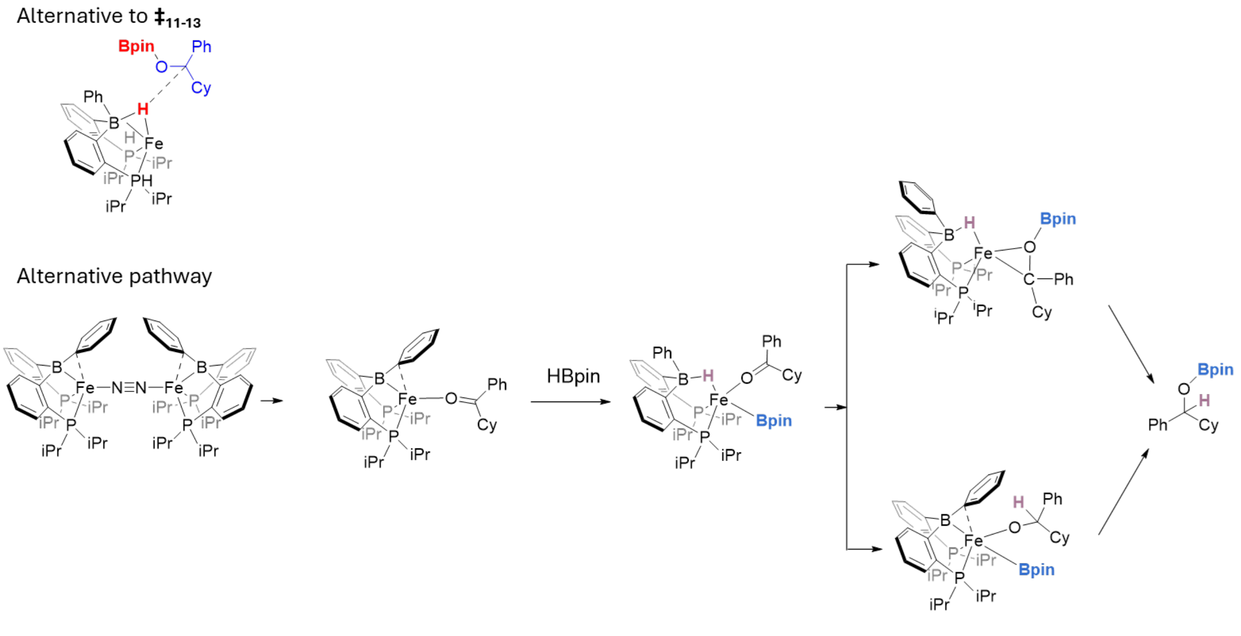

Figure S10. Schematic of the alternative pathways considered. The value of the spin quantum number is S=1 at each Fe centre.

Species	E (wb97X-D3)	Calculated <S^2> values		
		Total S2	no. 1	no. 2
A	-7378.878945	7.505306		
ts(A-10)+ketone	-7378.868771	7.796389		
10 + ketone	-7378.874328	6.901314		
ts(10-10a)+ketone	-7378.861224	7.283765		
10a+ketone	-7378.874299	6.902595		
ts(10a-11)+ketone	-7378.860535	6.551561		
11+9	-7378.87115	***7.3208***	2.398106	2.388085
ts(11-13)+9	-7378.865431	6.98191		
(11-13i)+9	-7378.88209	***8.756***	3.272323	2.388085
ts(11-13)[2]	-7378.840361	6.98191		
13+9	-7378.925962	***7.599***	2.563629	2.388085
ts(13-prod)+9	-7378.914624	***7.594***	2.56074	2.388085
prod + A	-7378.938118	7.505306		

Table S4. Energies (Hartree) and <S^2> values from the DFT calculations. Where calculations were performed on separate components, the individual <S^2> values are given, along with a calculated final value based on solving the equation S(S+1) for S for each component.

NMR spectra of compounds

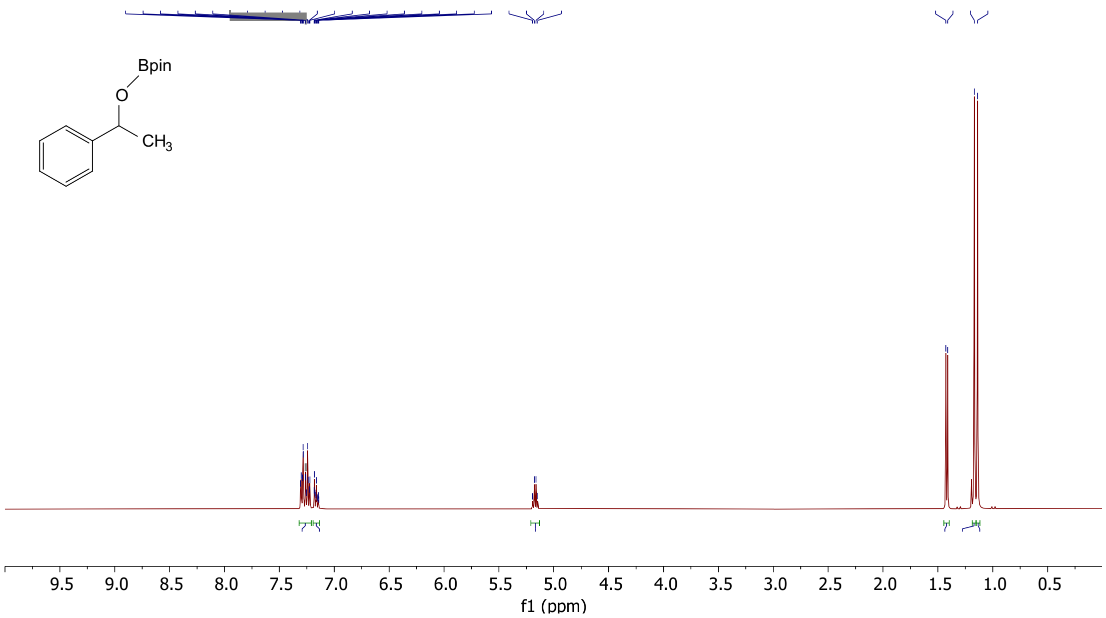

Figure S11: ^{1}H NMR spectrum (400 MHz, $CDCl_3$) of **2a**

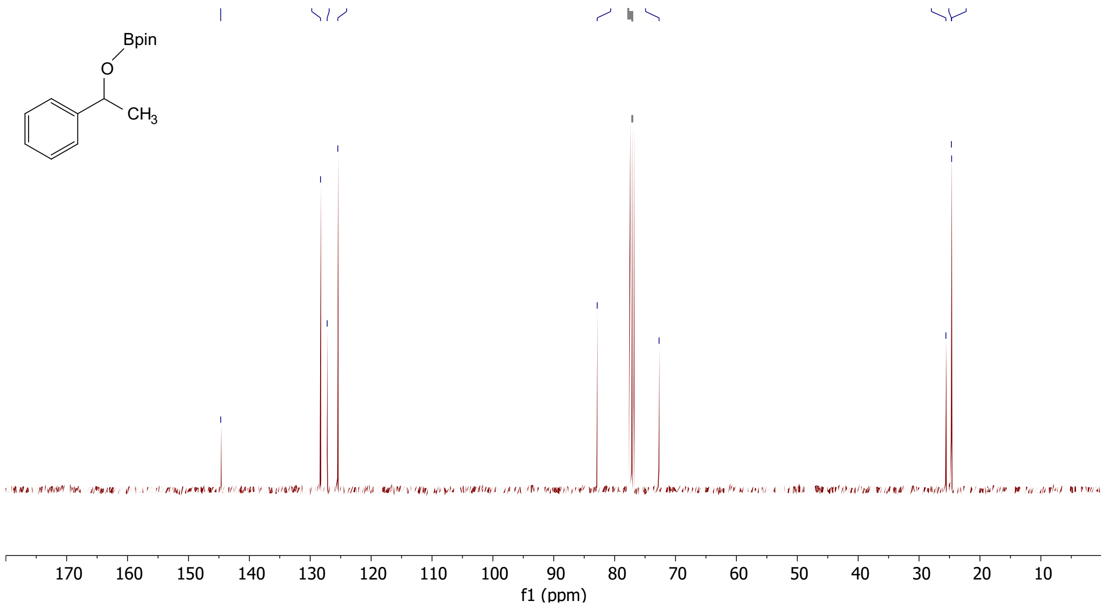

Figure S12: $^{13}C\{^{1}H\}$ NMR spectrum (101 MHz, $CDCl_3$) of **2a**

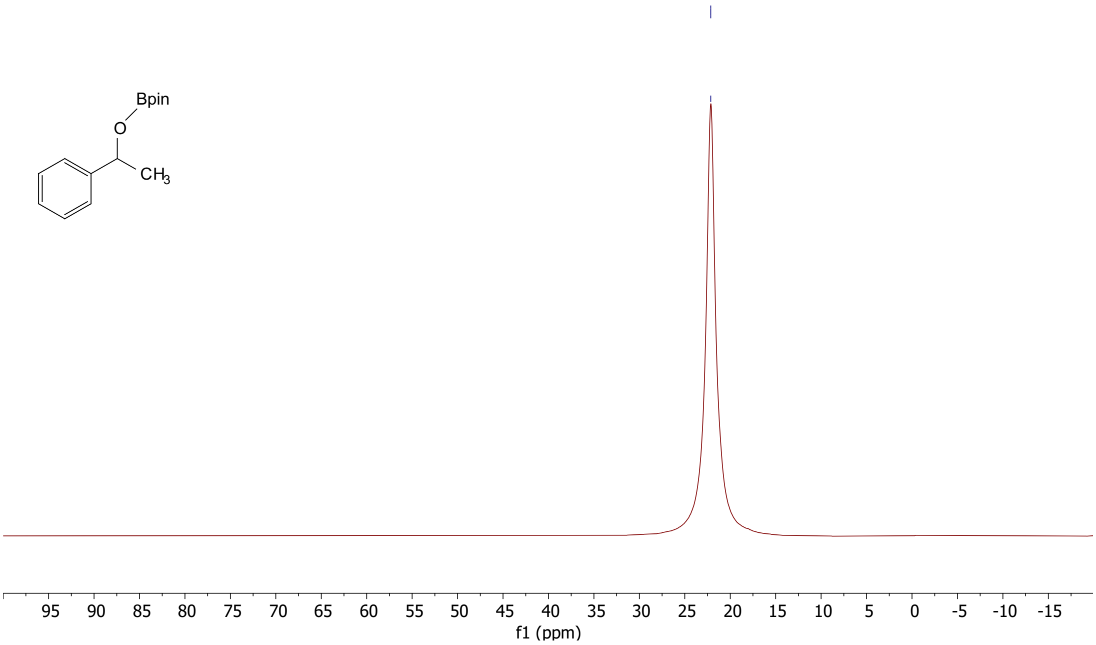

Figure S13: ^{11}B NMR spectrum (128 MHz, $CDCl_3$) of **2a**

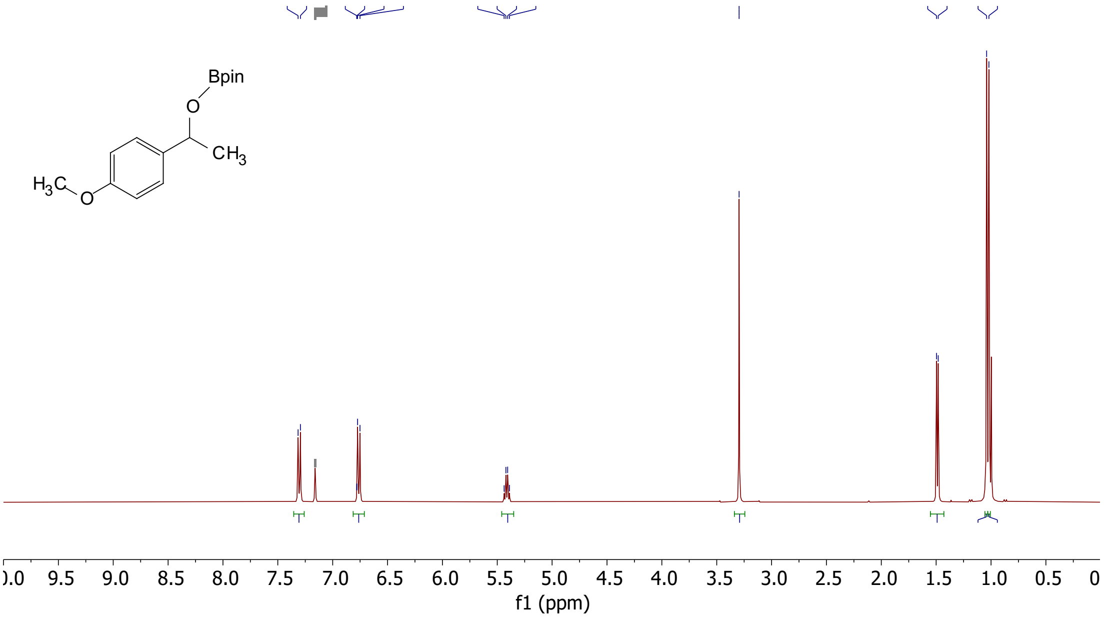

Figure S14: ^{1}H NMR spectrum (400 MHz, C_6D_6) of **2b**

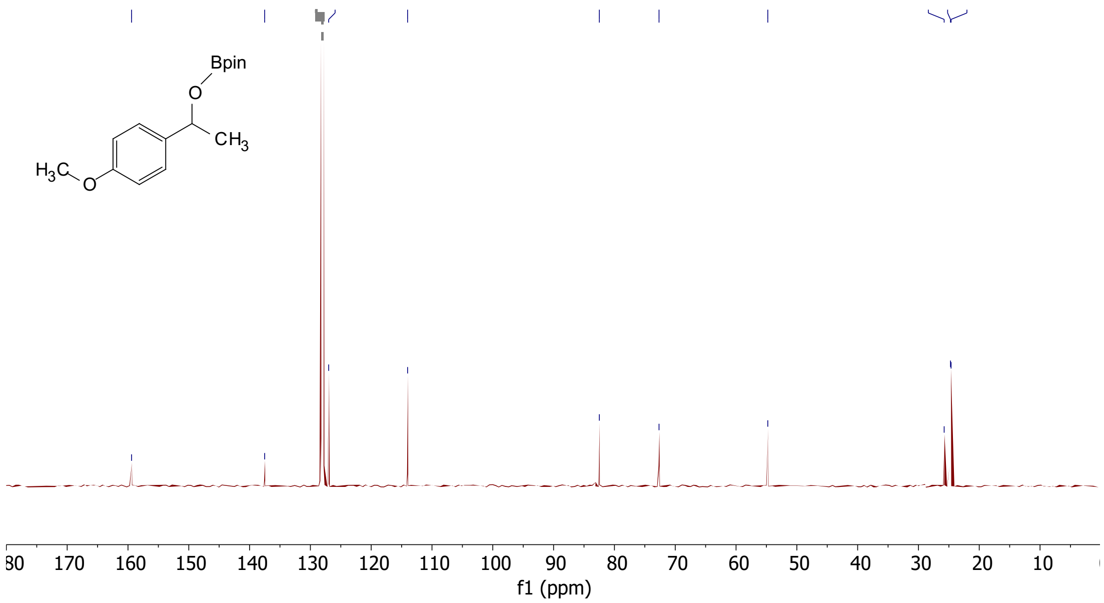

Figure S15: $^{13}C\{^{1}H\}$ NMR spectrum (101 MHz, C_6D_6) of **2b**

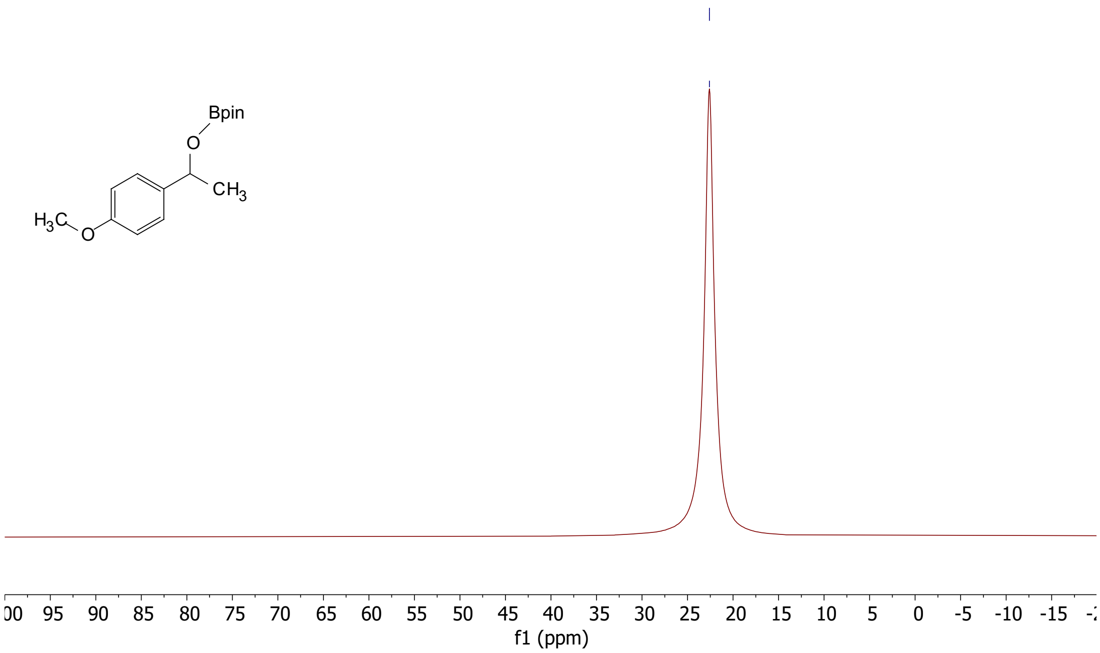

Figure S16: ^{11}B NMR spectrum (128 MHz, C_6D_6) of **2b**

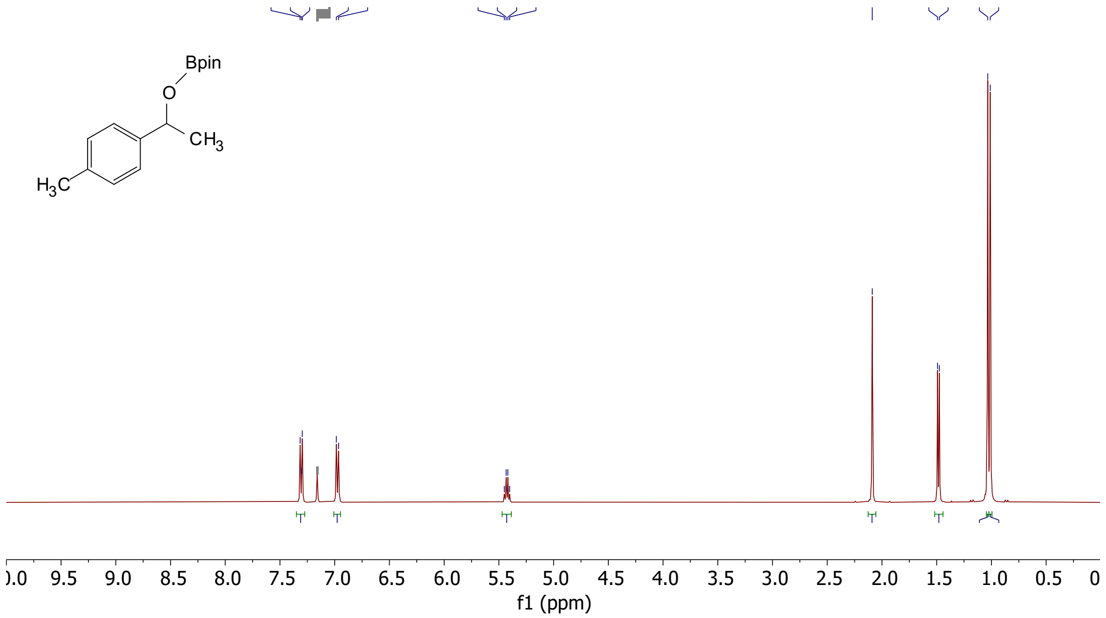

Figure S17: ^{1}H NMR spectrum (400 MHz, C_6D_6) of **2c**

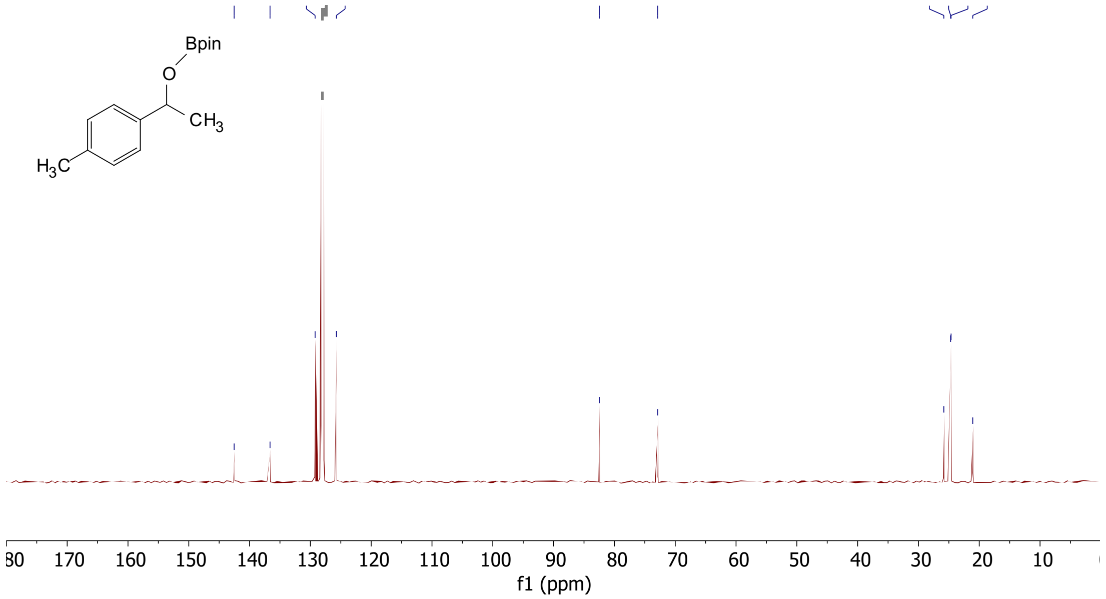

Figure S18: $^{13}C\{^{1}H\}$ NMR spectrum (101 MHz, C_6D_6) of **2c**

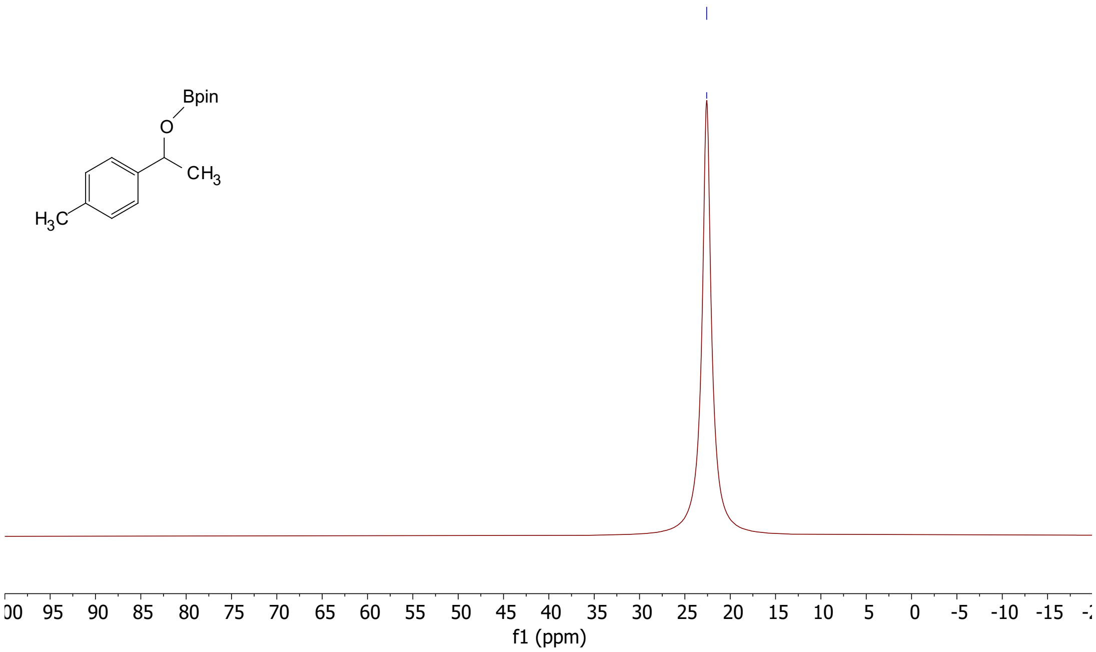

Figure S19: ^{11}B NMR spectrum (128 MHz, C_6D_6) of **2c**

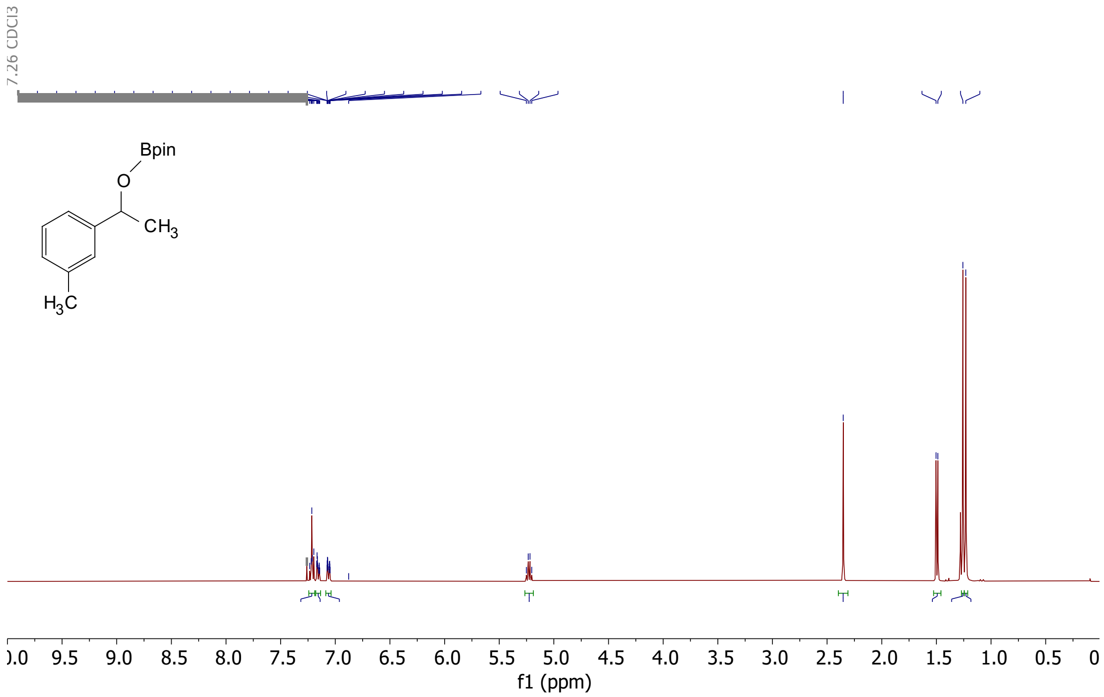

Figure S20: ^{1}H NMR spectrum (400 MHz, $CDCl_3$) of **2d**

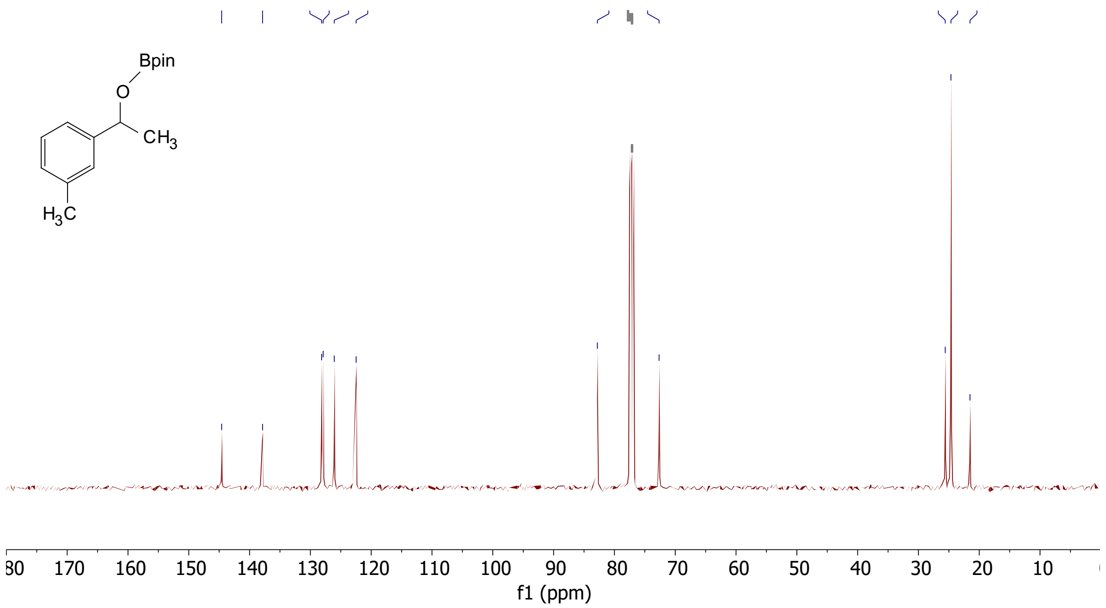

Figure S21: $^{13}C\{^{1}H\}$ NMR spectrum (101 MHz, $CDCl_3$) of **2d**

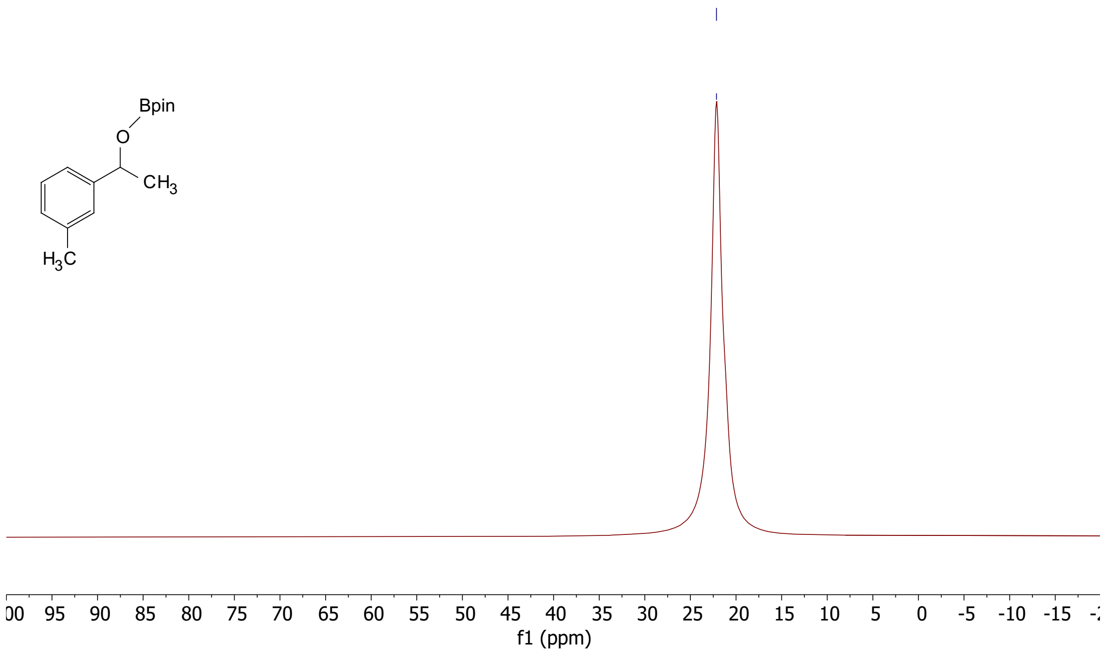

Figure S22: ^{11}B NMR spectrum (128 MHz, $CDCl_3$) of **2d**

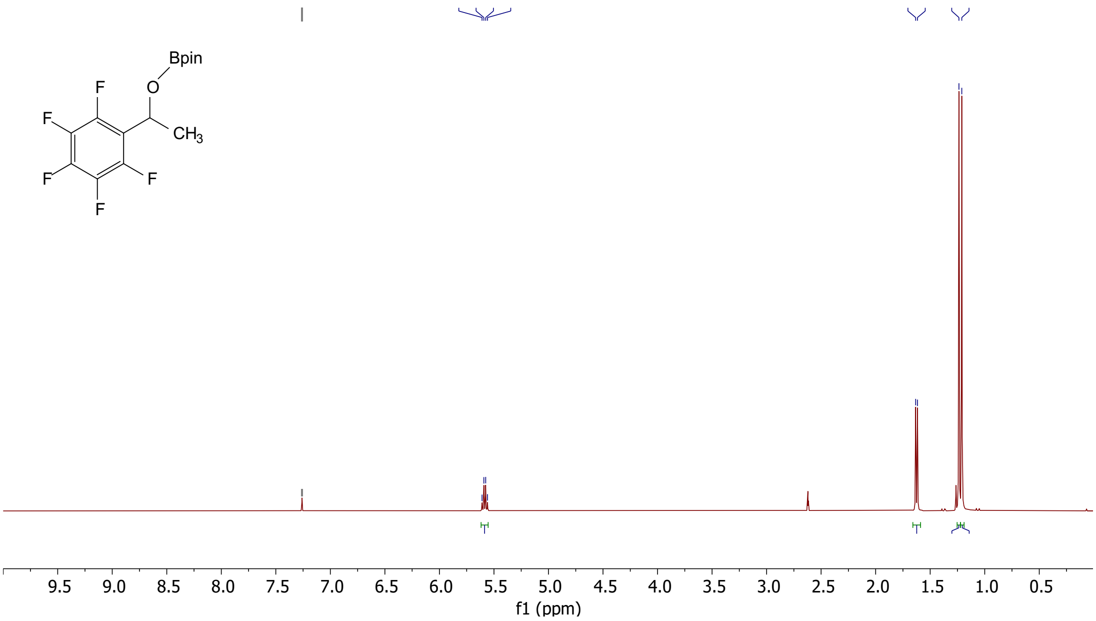

Figure S23: ^{1}H NMR spectrum (400 MHz, $CDCl_3$) of **2e**

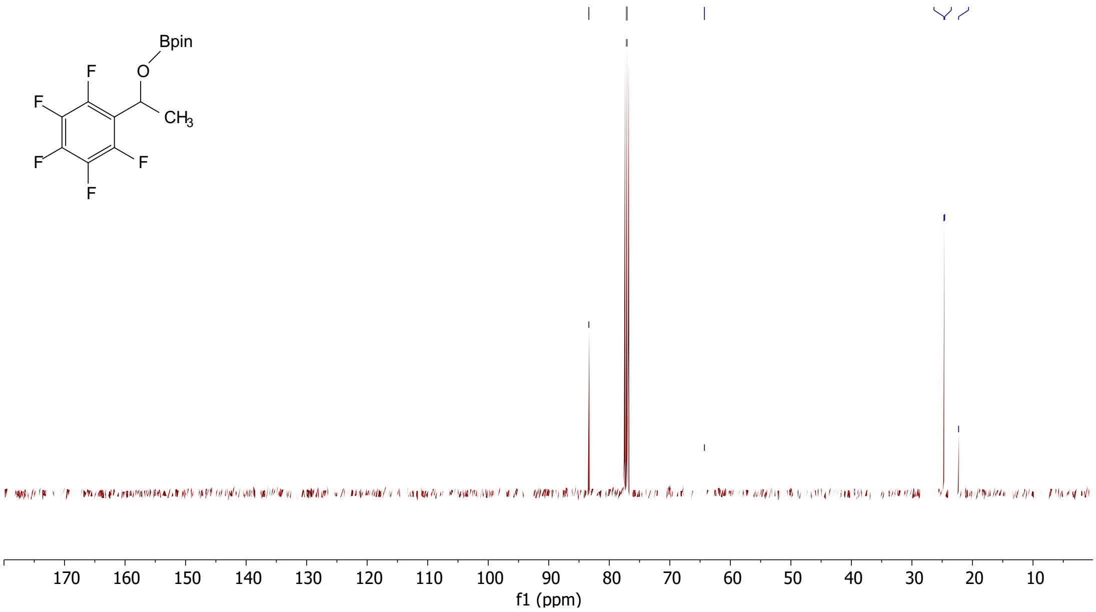

Figure S24: $^{13}C\{^{1}H\}$ NMR spectrum (101 MHz, $CDCl_3$) of **2e**

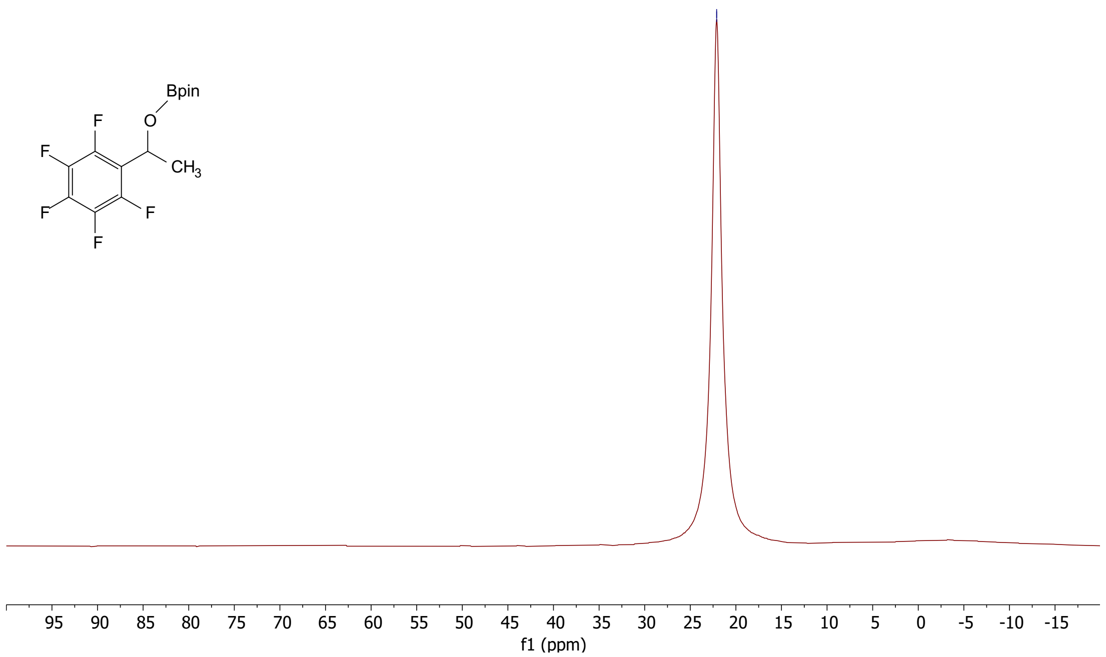

Figure S25: ^{11}B NMR spectrum (128 MHz, $CDCl_3$) of **2e**

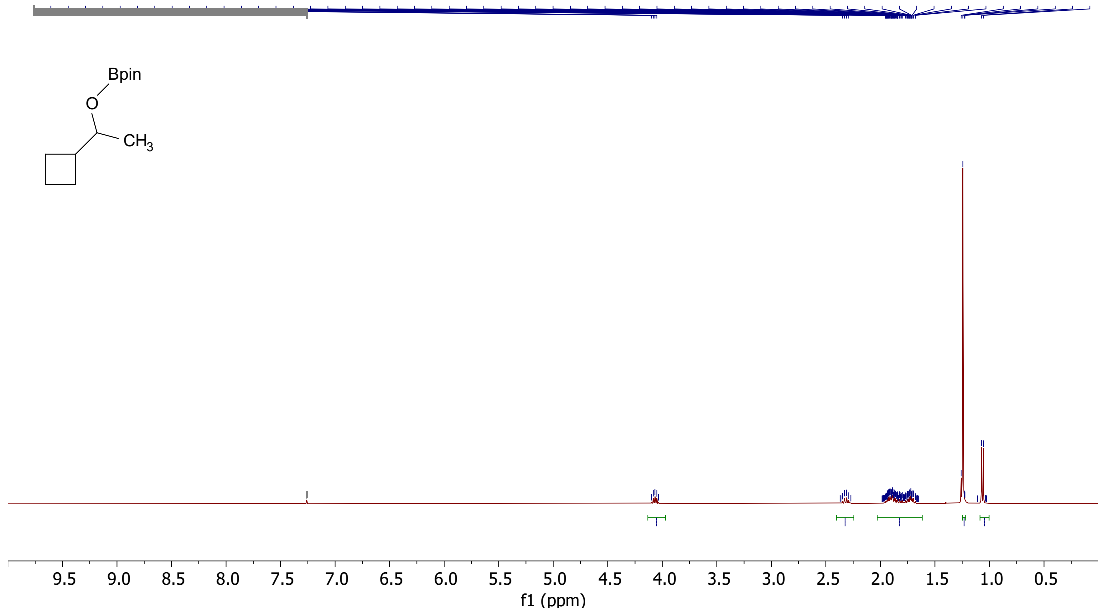

Figure S26: ^{1}H NMR spectrum (400 MHz, $CDCl_3$) of **2f**

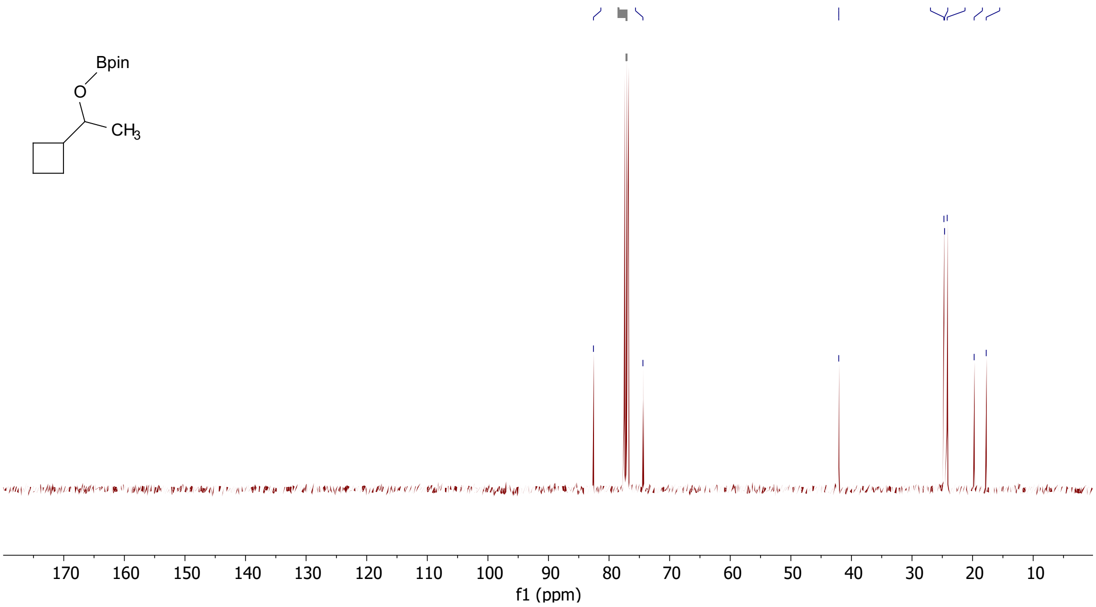

Figure S27: $^{13}C\{^{1}H\}$ NMR spectrum (101 MHz, $CDCl_3$) of **2f**

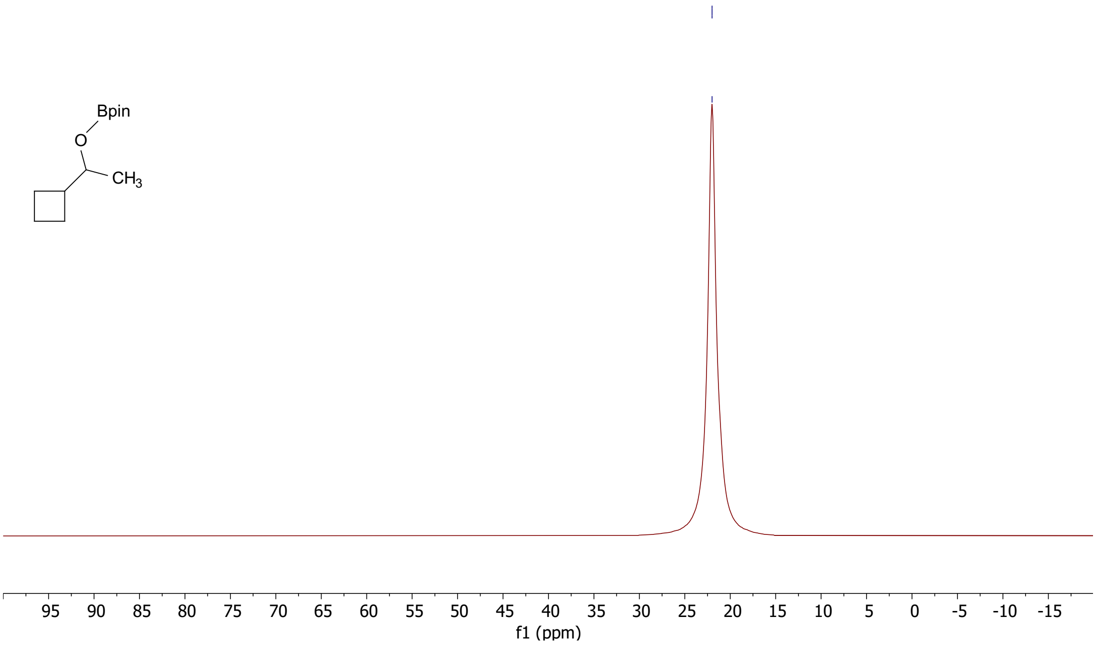

Figure S28: ^{11}B NMR spectrum (128 MHz, $CDCl_3$) of **2f**

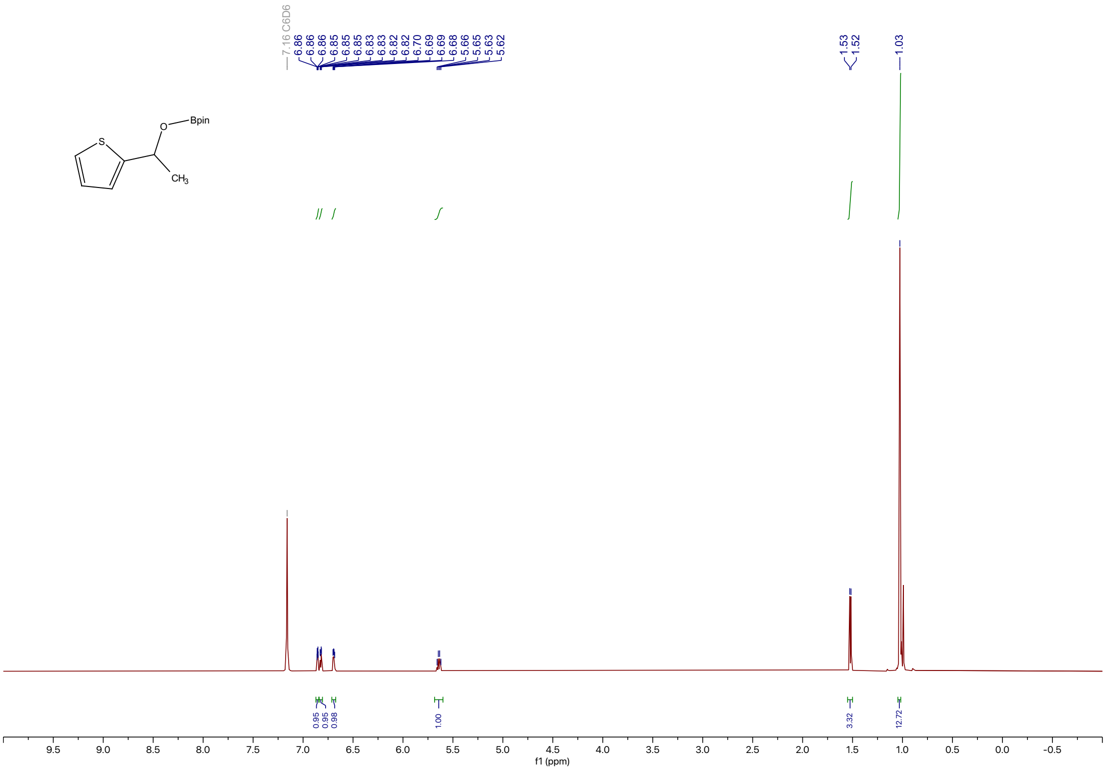

Figure S29: ^{1}H NMR spectrum (500 MHz, C_6D_6) of **2g**

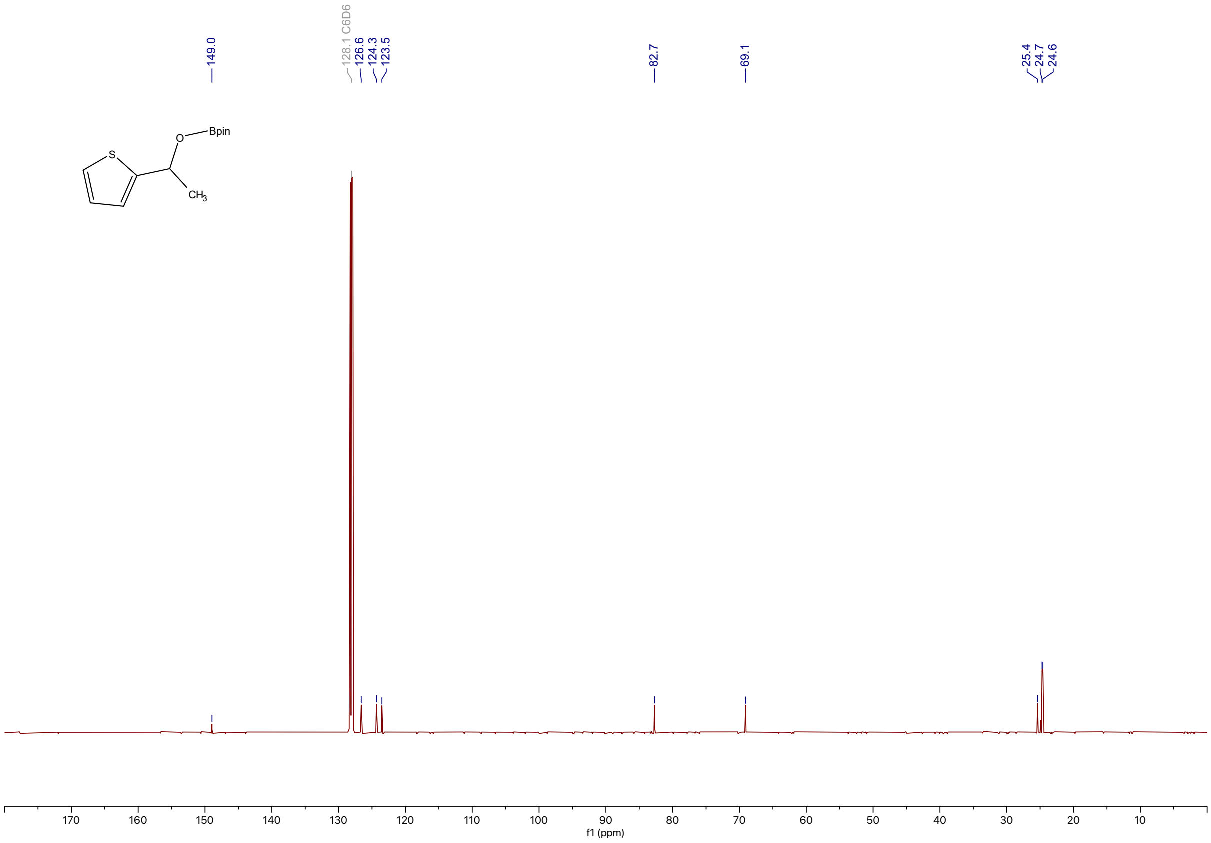

Figure S30: $^{13}C\{^{1}H\}$ NMR spectrum (125 MHz, C_6D_6) of **2g**

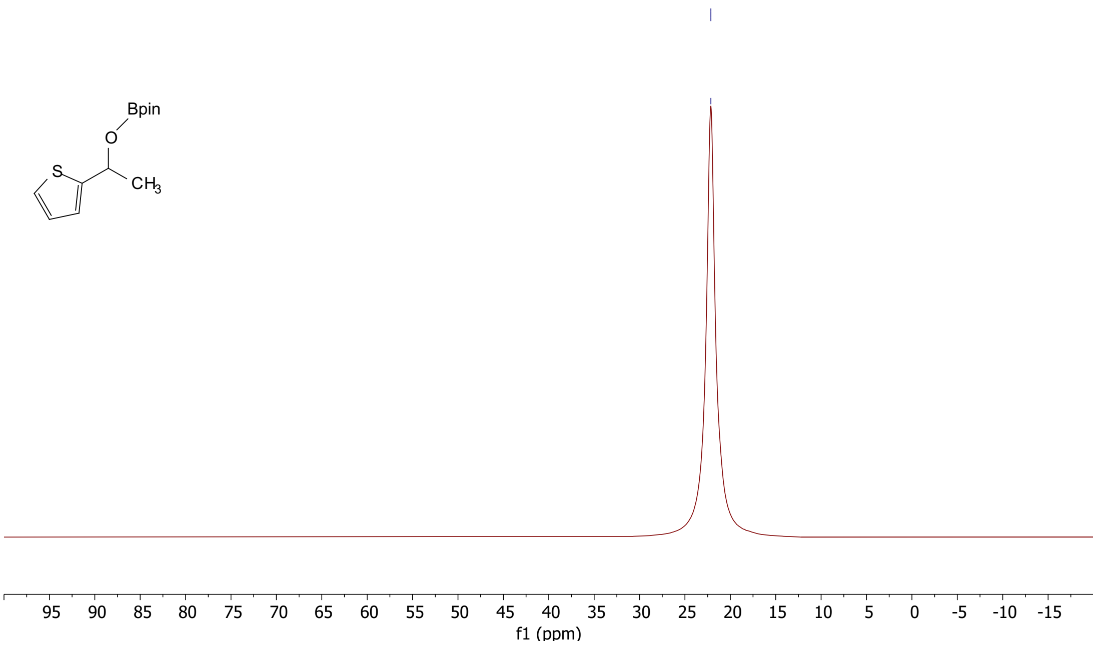

Figure S31: ^{11}B NMR spectrum (128 MHz, C_6D_6) of **2g**

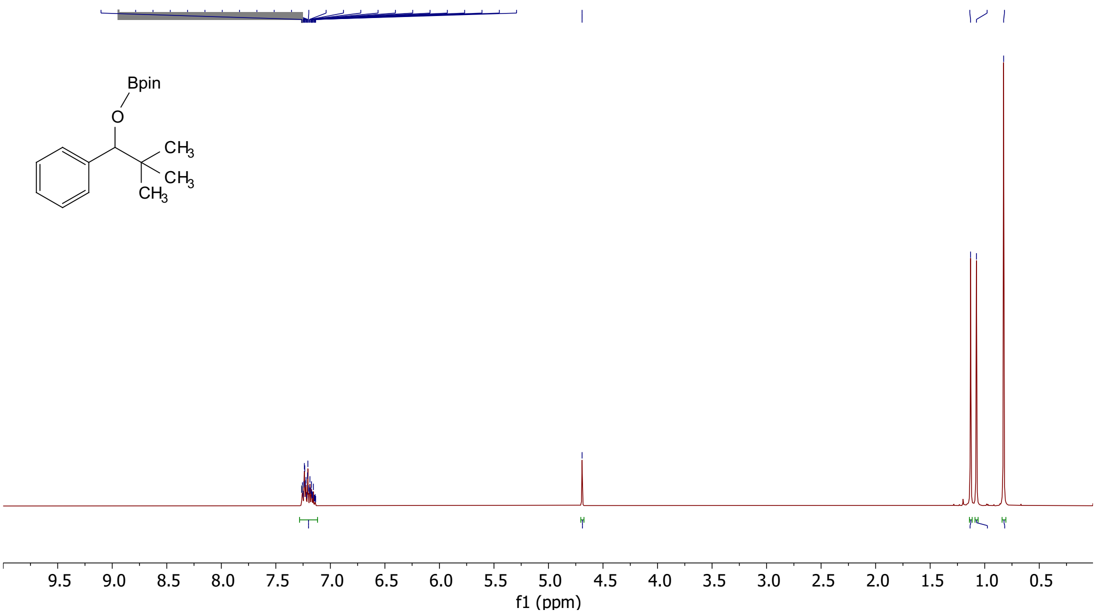

Figure S32: ^{1}H NMR spectrum (400 MHz, $CDCl_3$) of **2h**

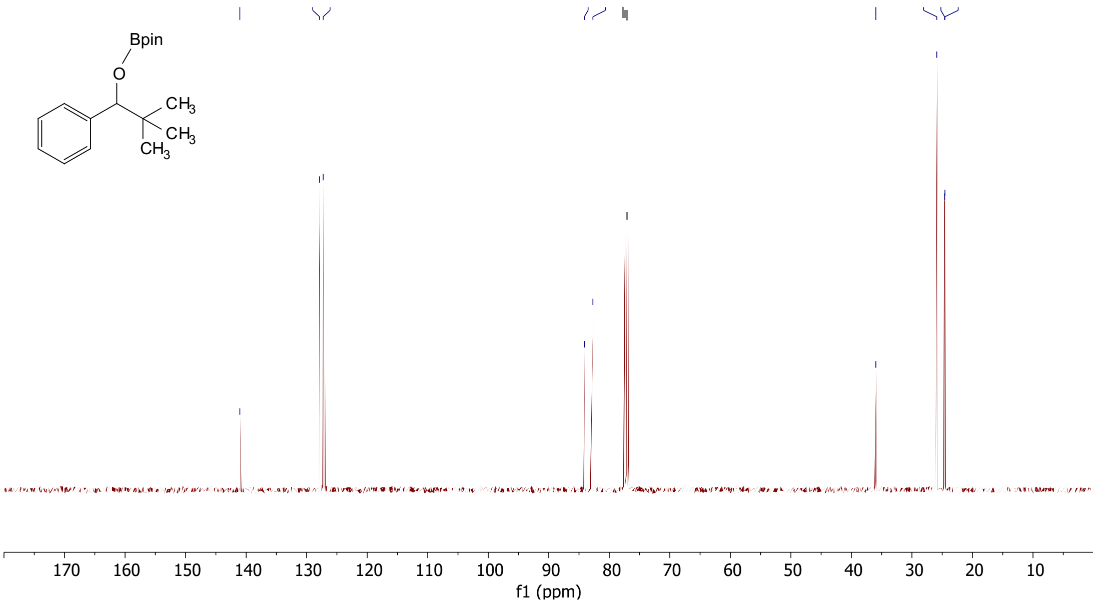

Figure S33: $^{13}C\{^{1}H\}$ NMR spectrum (101 MHz, $CDCl_3$) of **2h**

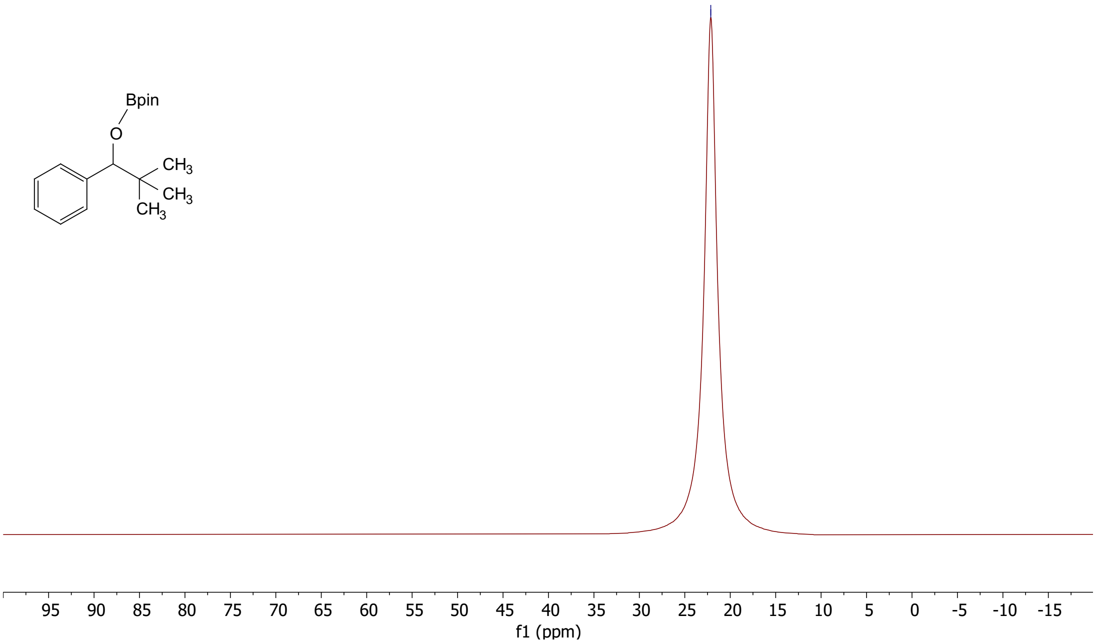

Figure S34: ^{11}B NMR spectrum (128 MHz, $CDCl_3$) of **2h**

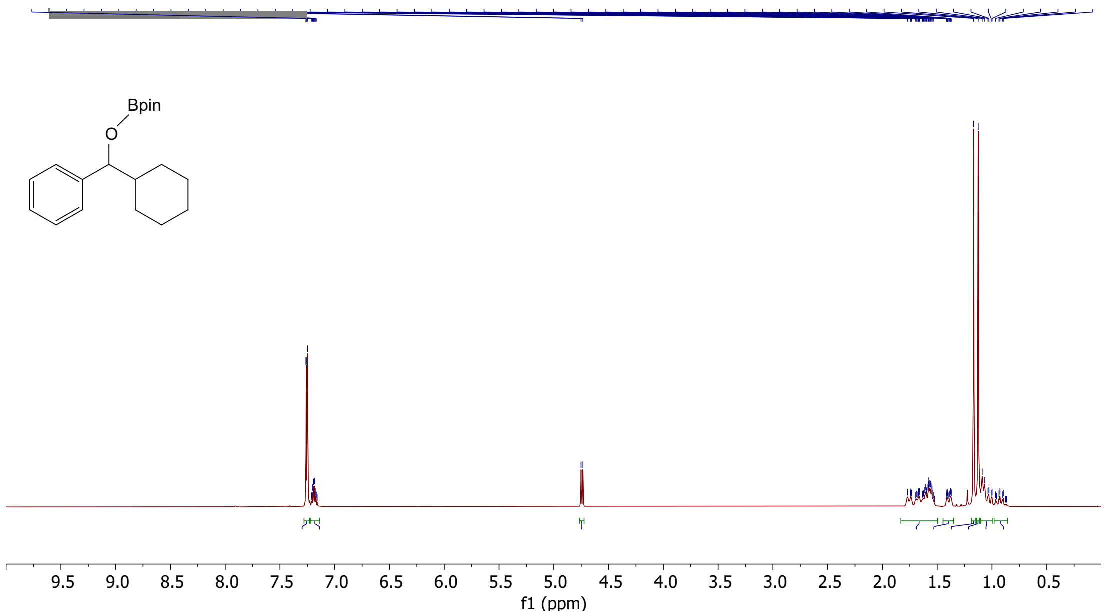

Figure S35: ^{1}H NMR spectrum (400 MHz, $CDCl_3$) of **2i**

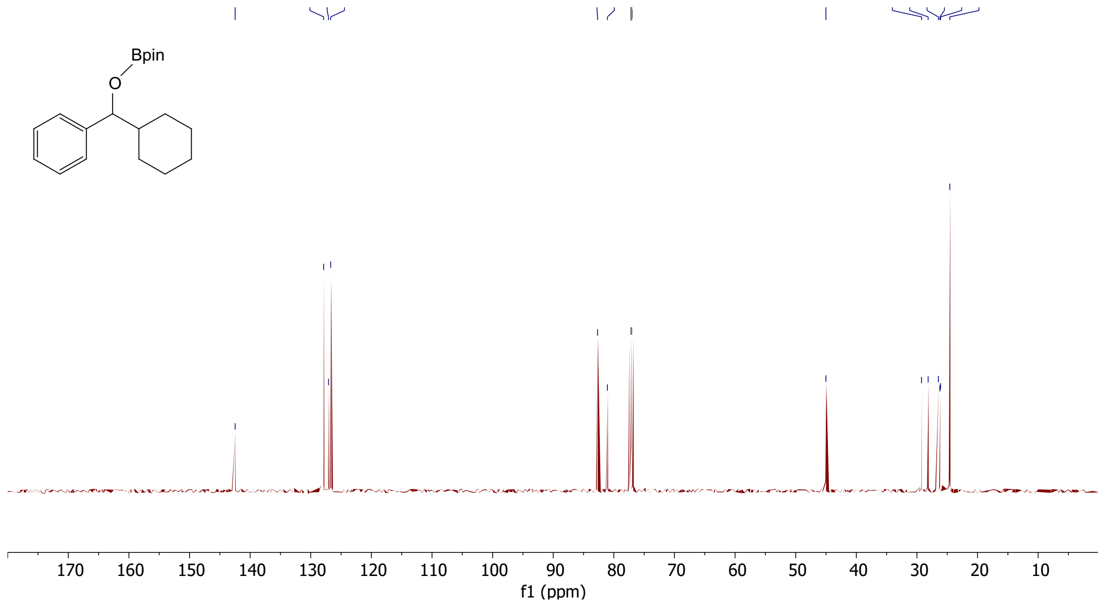

Figure S36: $^{13}C\{^{1}H\}$ NMR spectrum (101 MHz, $CDCl_3$) of **2i**

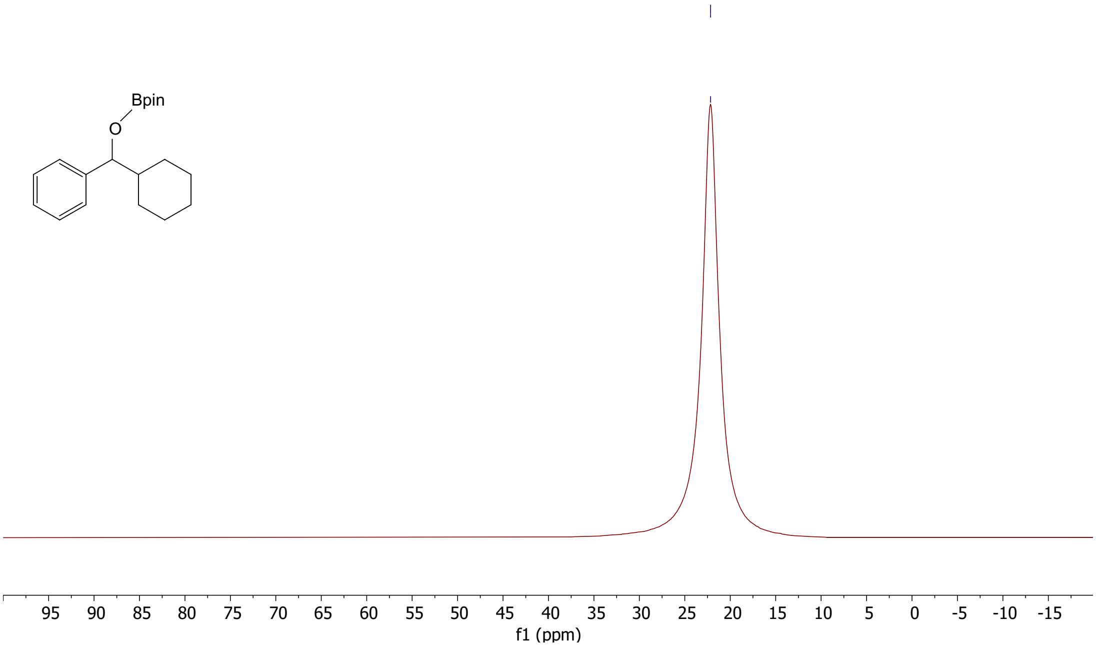

Figure S37: ^{11}B NMR spectrum (128 MHz, $CDCl_3$) of **2i**

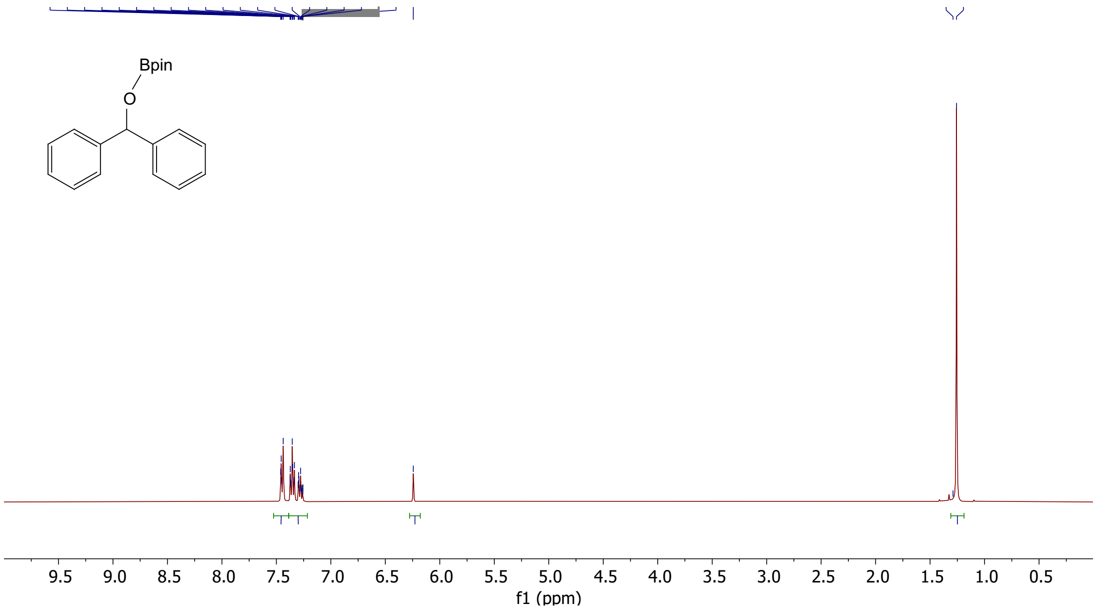

Figure S38: ^{1}H NMR spectrum (400 MHz, $CDCl_3$) of **2j**

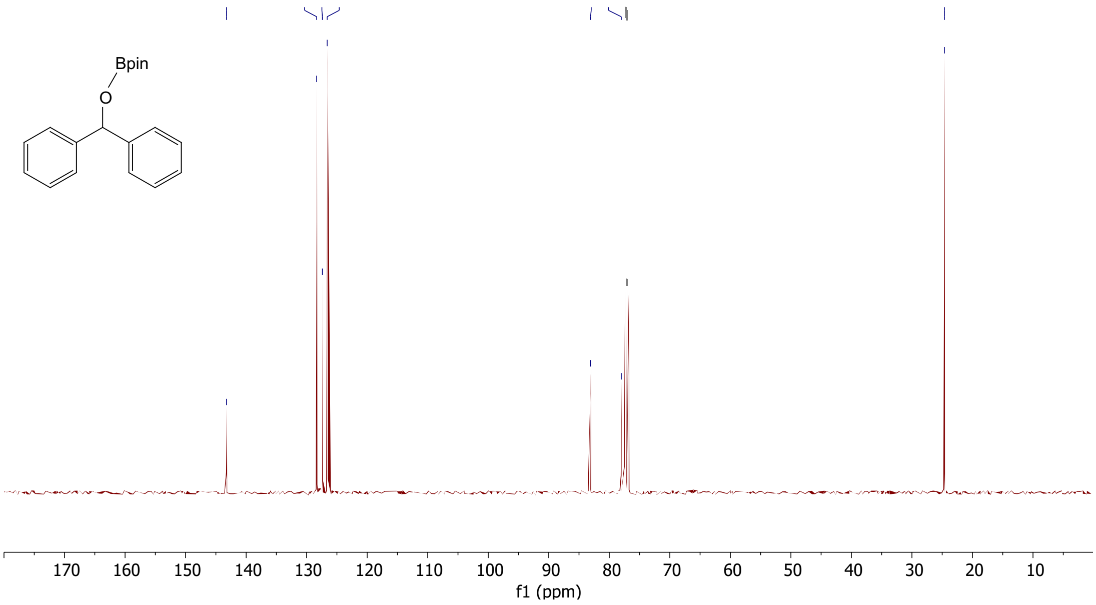

Figure S39: $^{13}C\{^1H\}$ NMR spectrum (101 MHz, $CDCl_3$) of **2j**

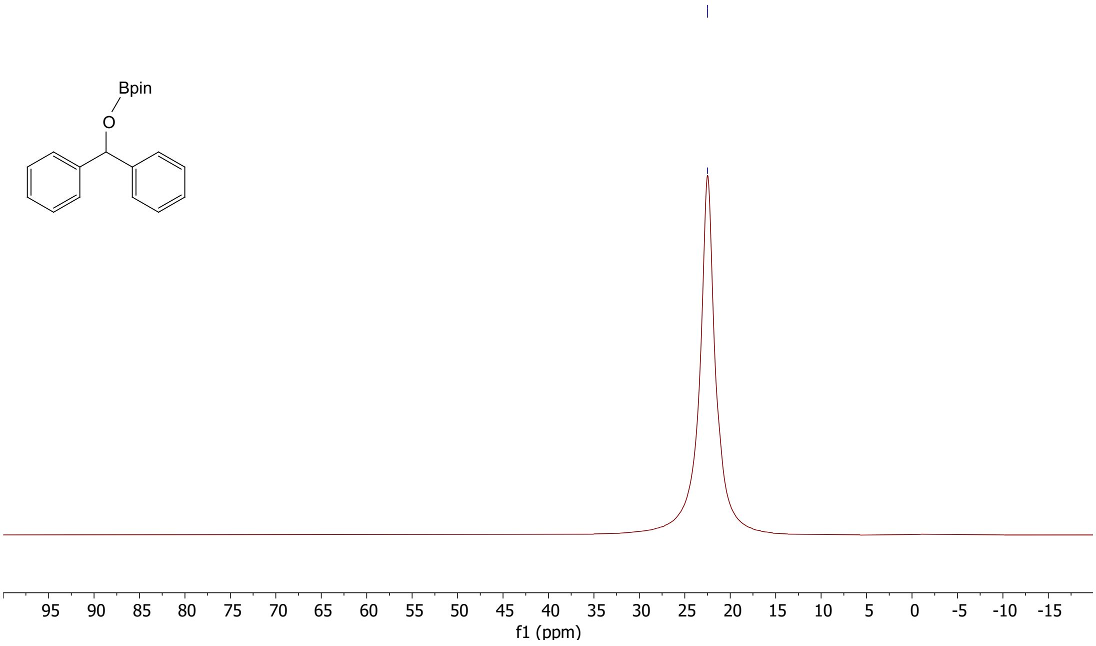

Figure S40: ^{11}B NMR spectrum (128 MHz, $CDCl_3$) of **2j**

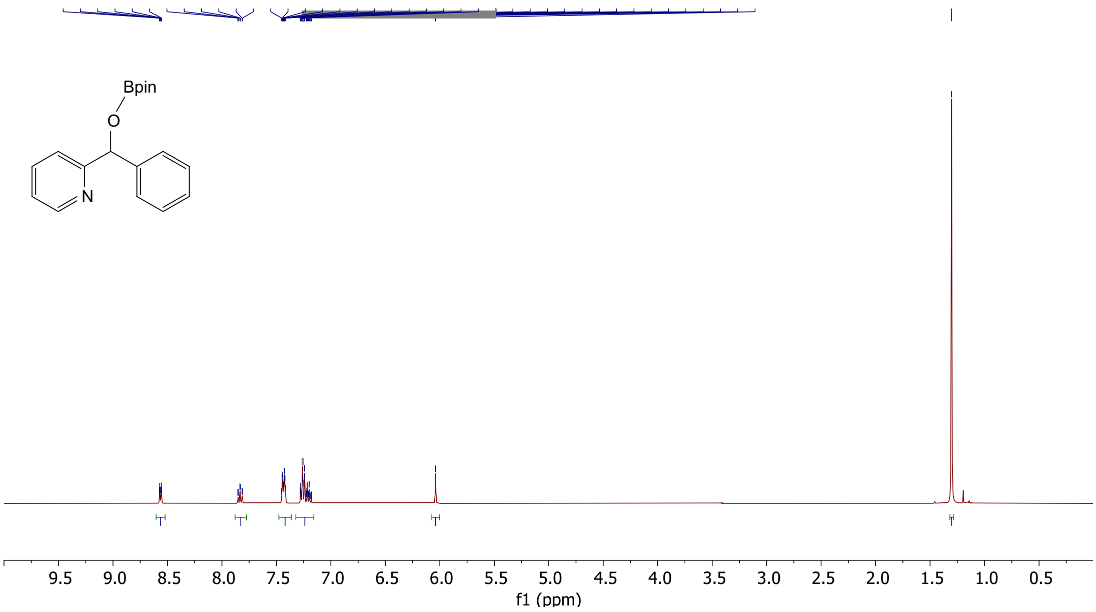

Figure S41: ^{1}H NMR spectrum (400 MHz, $CDCl_3$) of **2k**

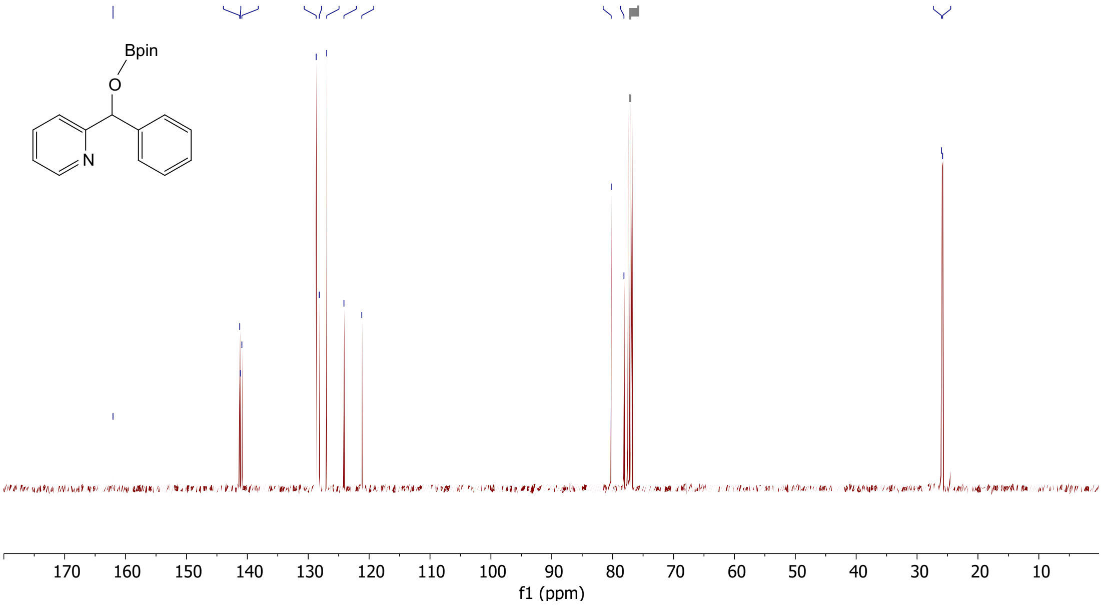

Figure S42: $^{13}C\{^{1}H\}$ NMR spectrum (101 MHz, $CDCl_3$) of **2k**

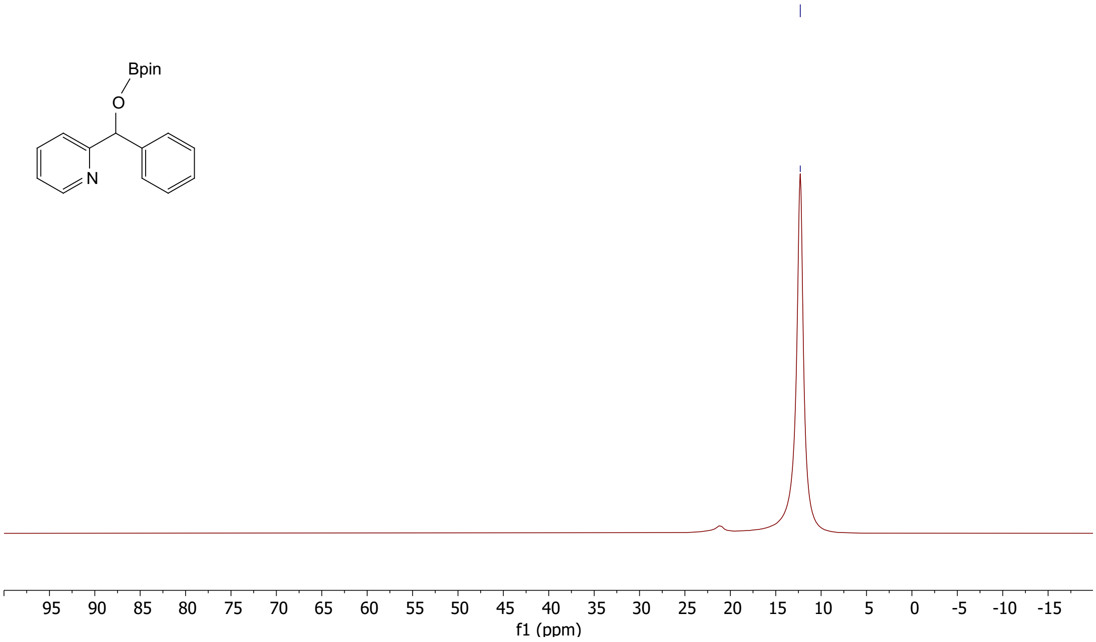

Figure S43: ^{11}B NMR spectrum (128 MHz, $CDCl_3$) of **2k**

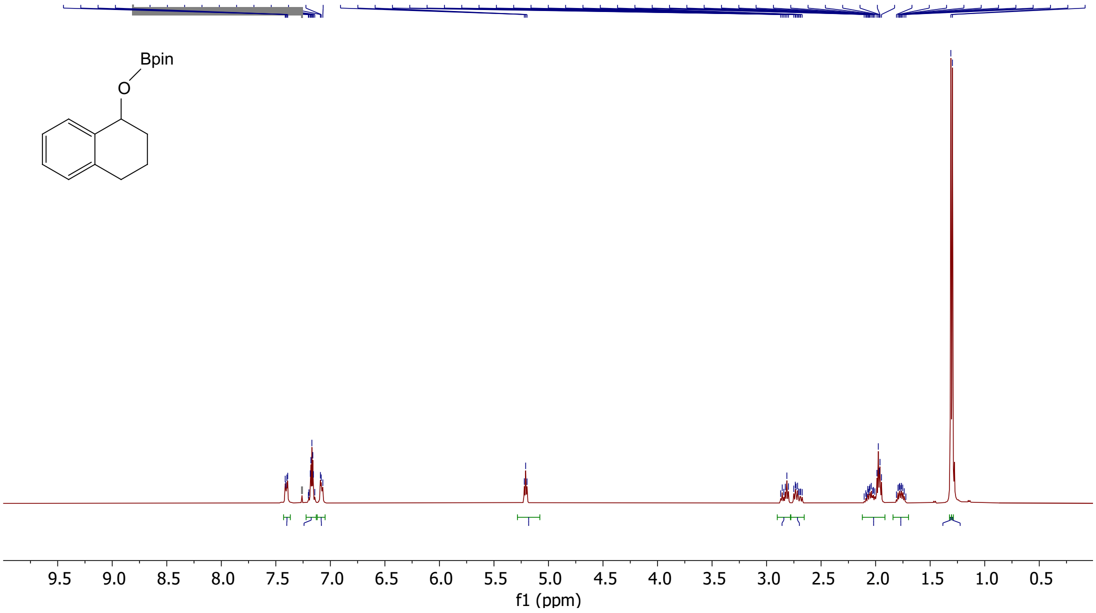

Figure S44: ^{1}H NMR spectrum (400 MHz, $CDCl_3$) of **2l**

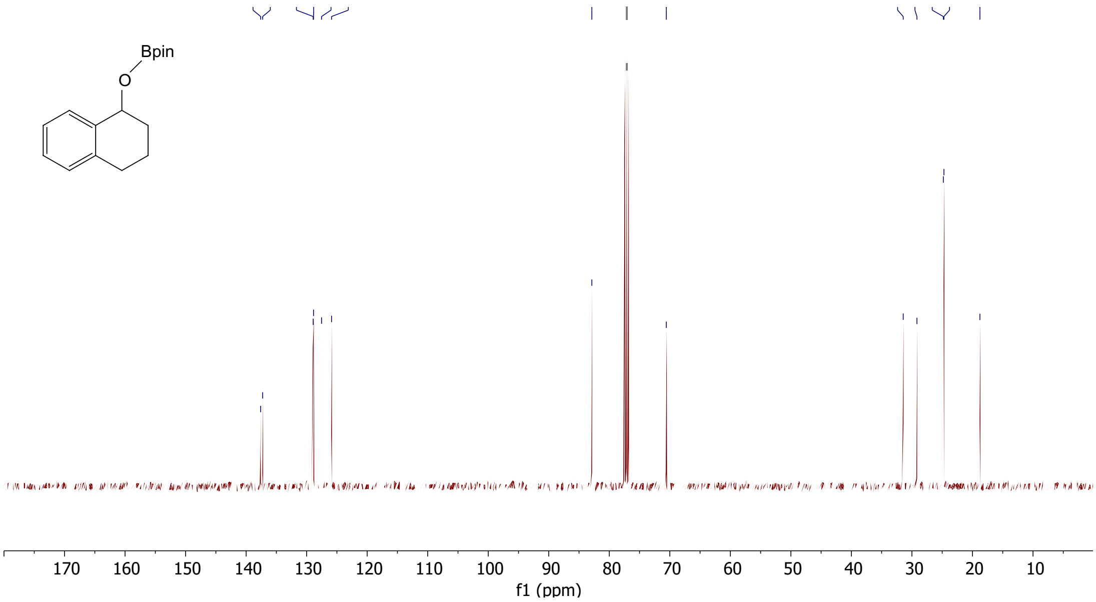

Figure S45: $^{13}C\{^{1}H\}$ NMR spectrum (101 MHz, $CDCl_3$) of **2l**

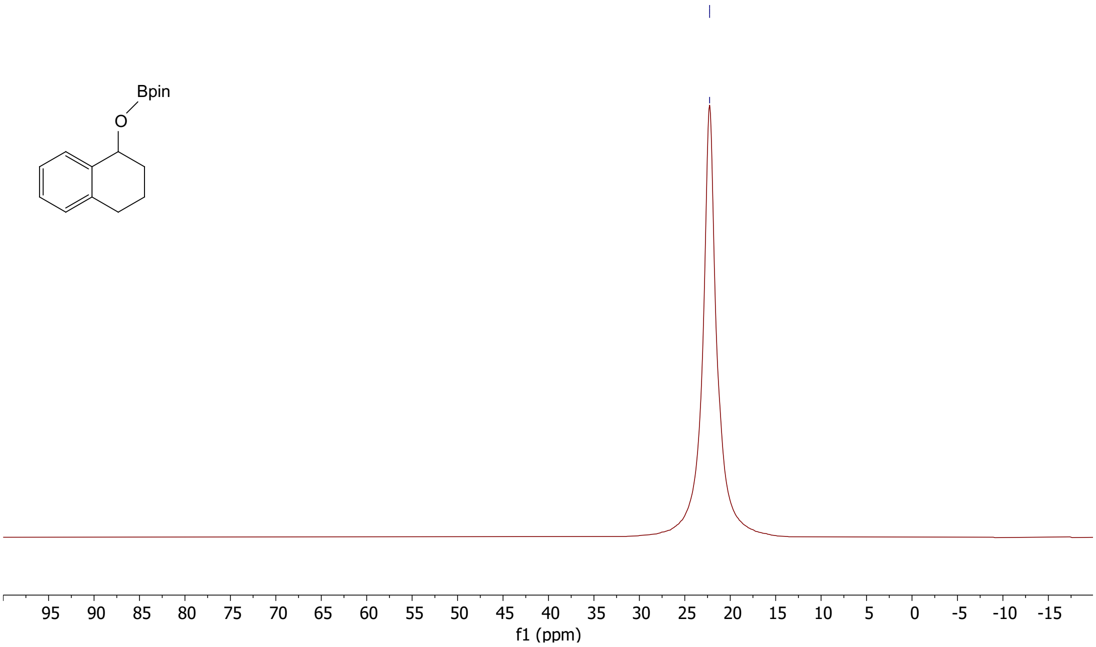

Figure S46: ^{11}B NMR spectrum (128 MHz, $CDCl_3$) of **2l**

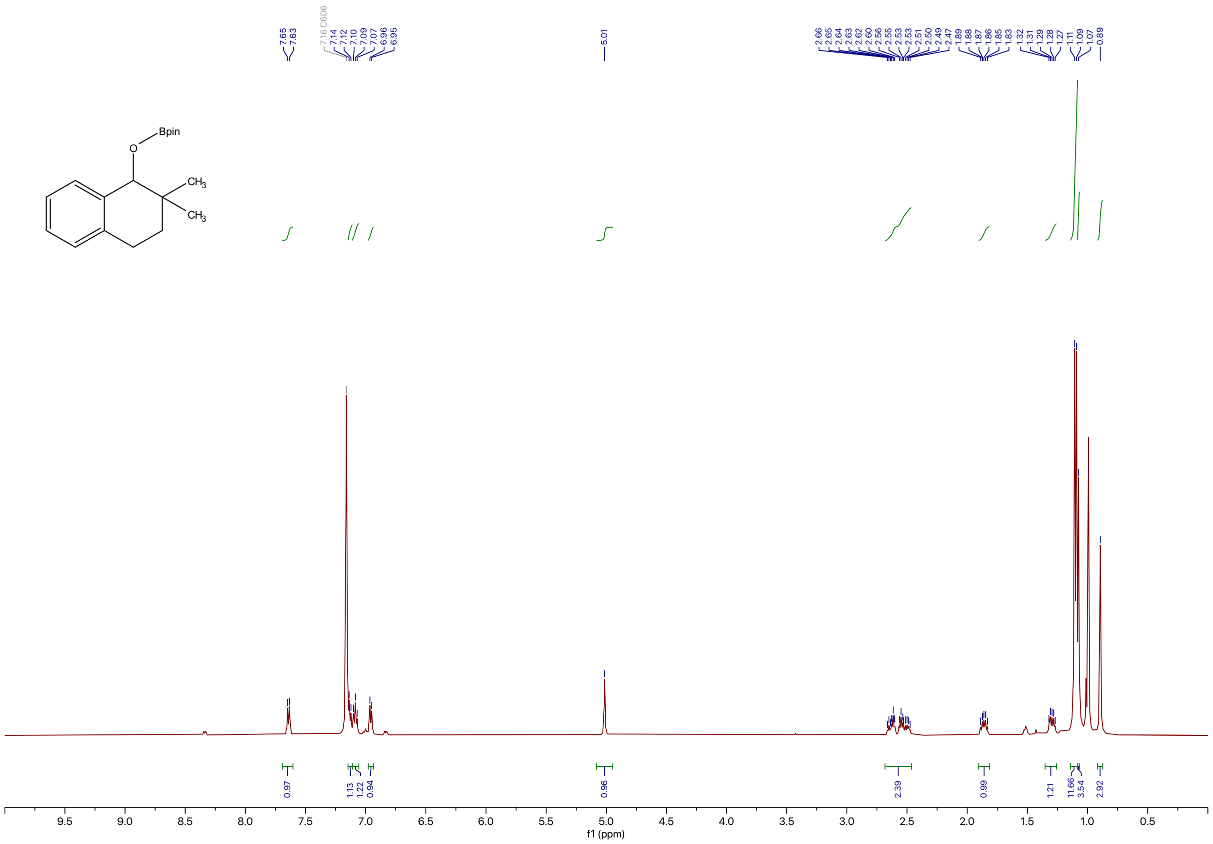

Figure S47: ^{1}H NMR spectrum (500 MHz, C_6D_6) of **2m**.

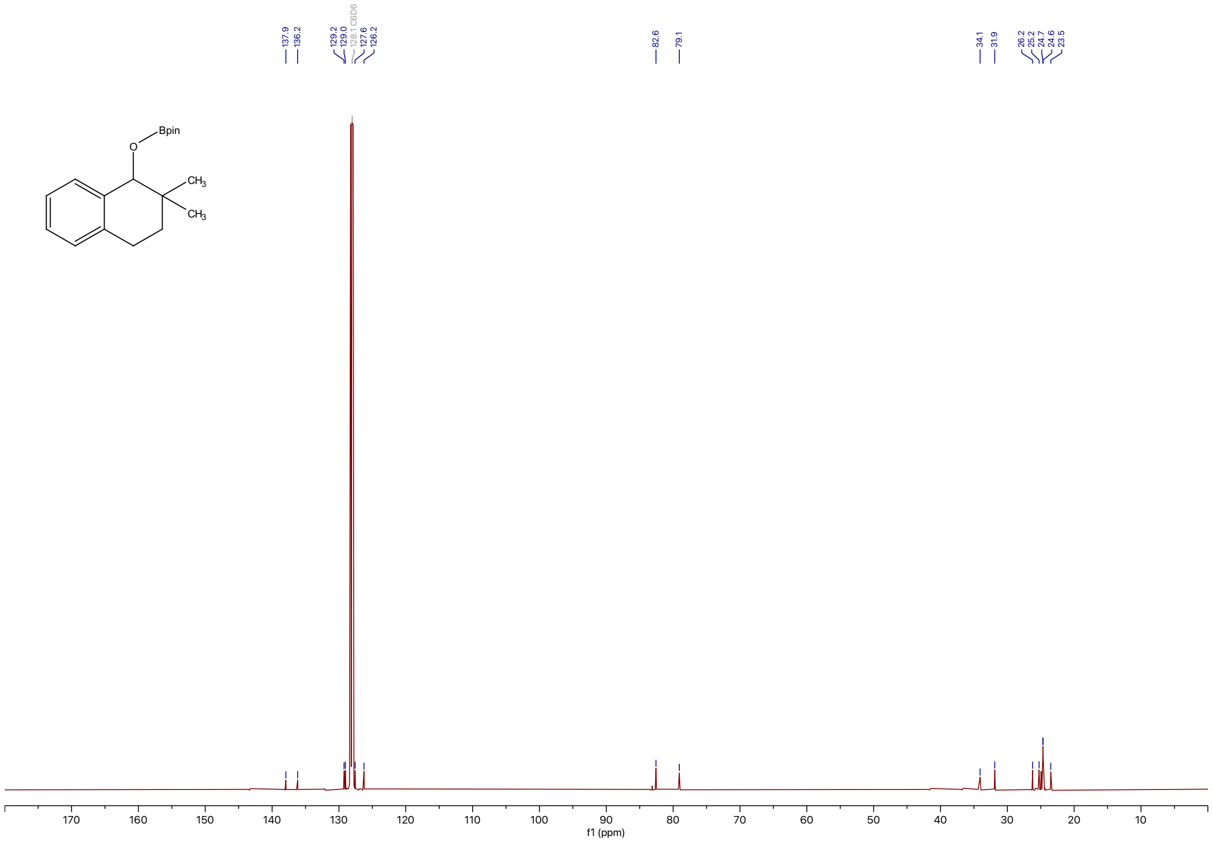

Figure S48: ^{13}C{^{1}H} NMR spectrum (126 MHz, C_6D_6) of **2m**.

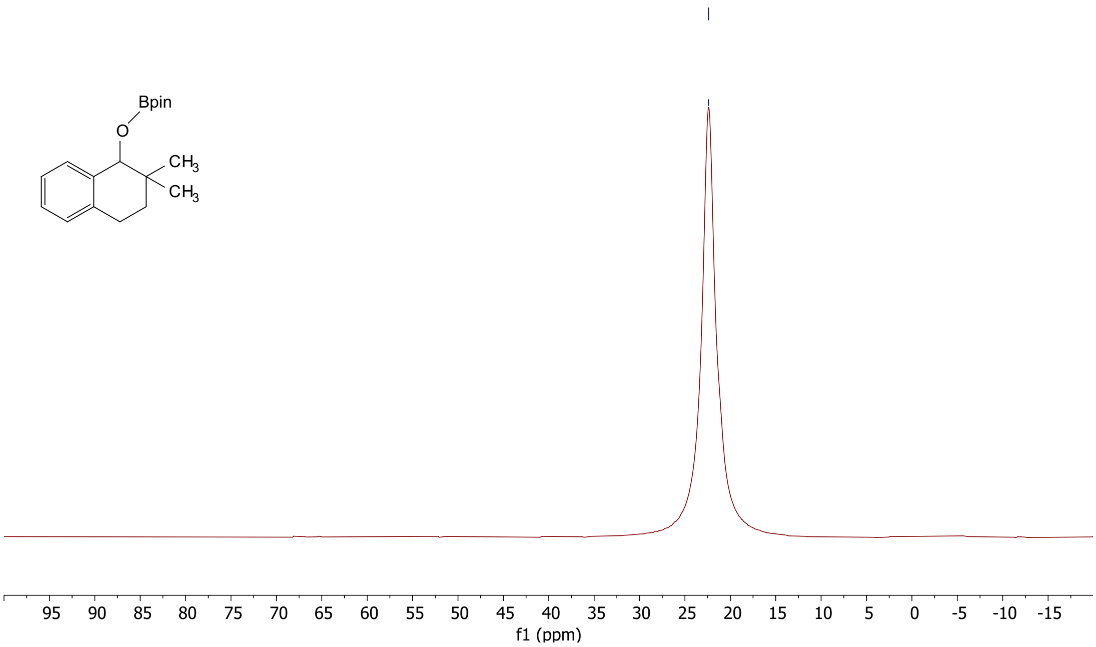

Figure S49: ^{11}B NMR spectrum (128 MHz, $CDCl_3$) of **2m**

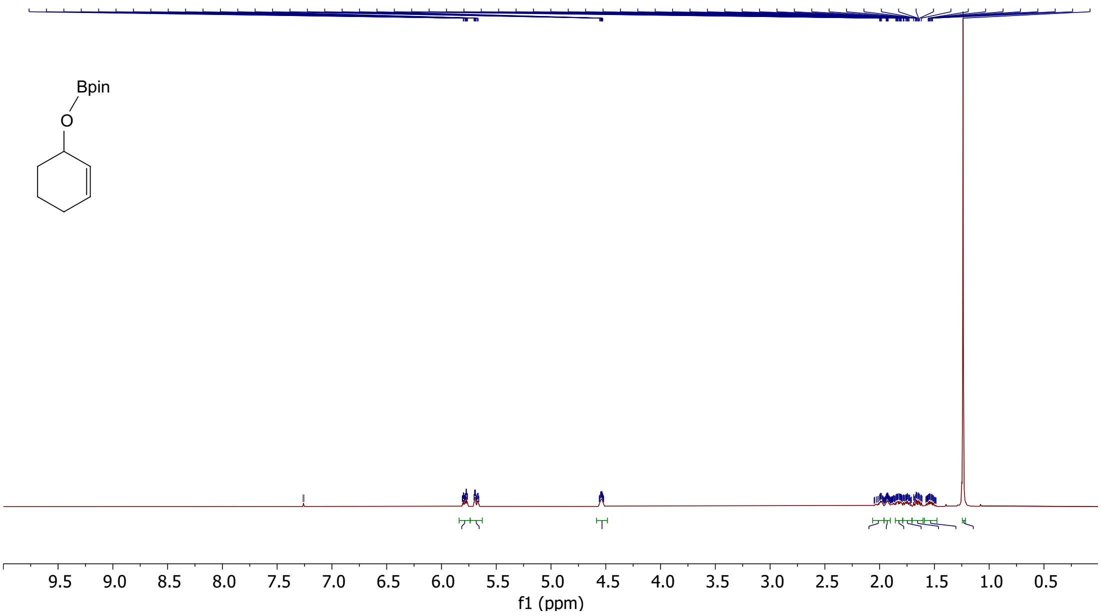

Figure S50: ^{1}H NMR spectrum (400 MHz, $CDCl_3$) of **2n**

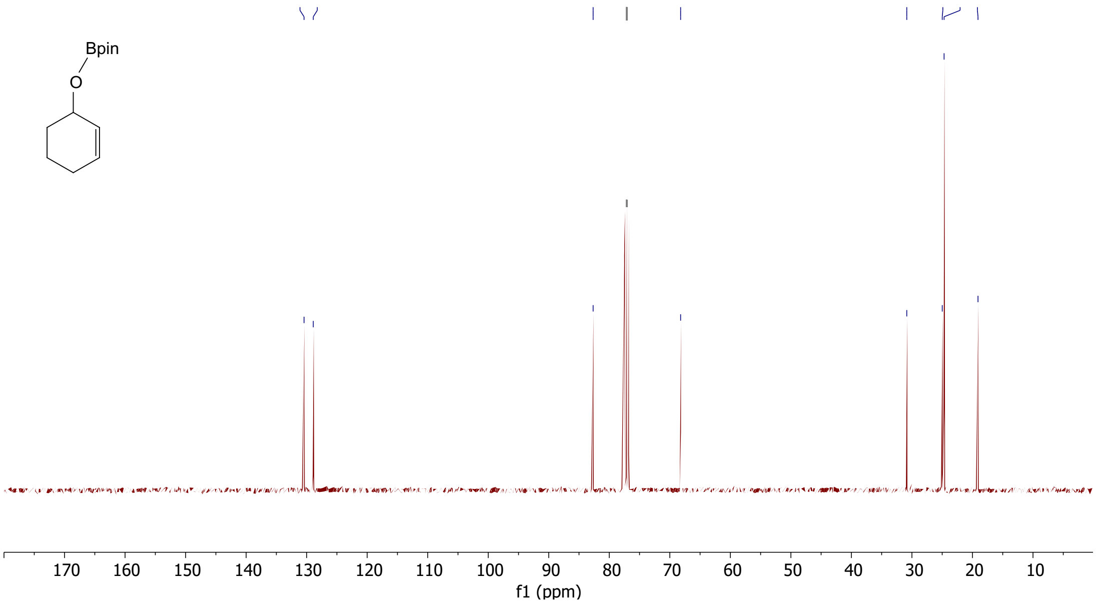

Figure S51: $^{13}C\{^{1}H\}$ NMR spectrum (101 MHz, $CDCl_3$) of **2n**

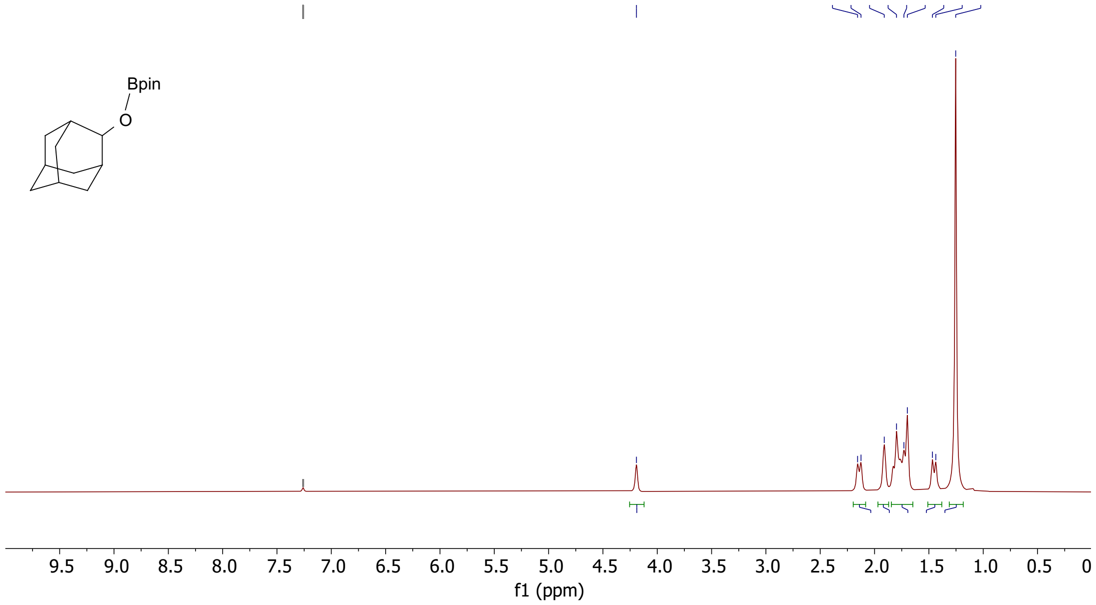

Figure S52*:* ^{1}H NMR spectrum (400 MHz, $CDCl_3$) of **2o**

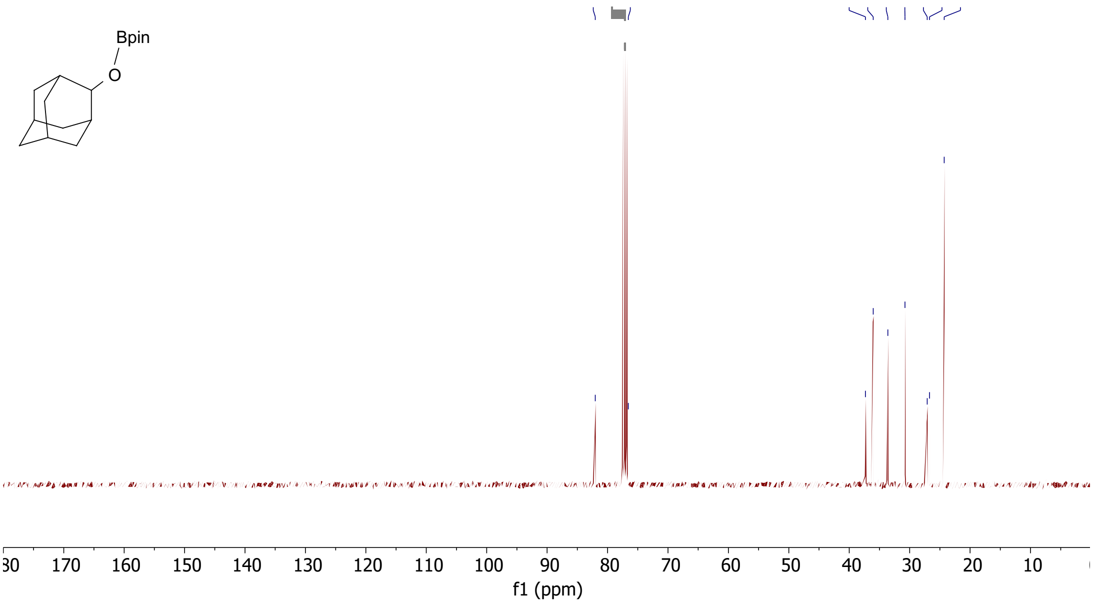

Figure S53: $^{13}C\{^{1}H\}$ NMR spectrum (101 MHz, $CDCl_3$) of **2o**

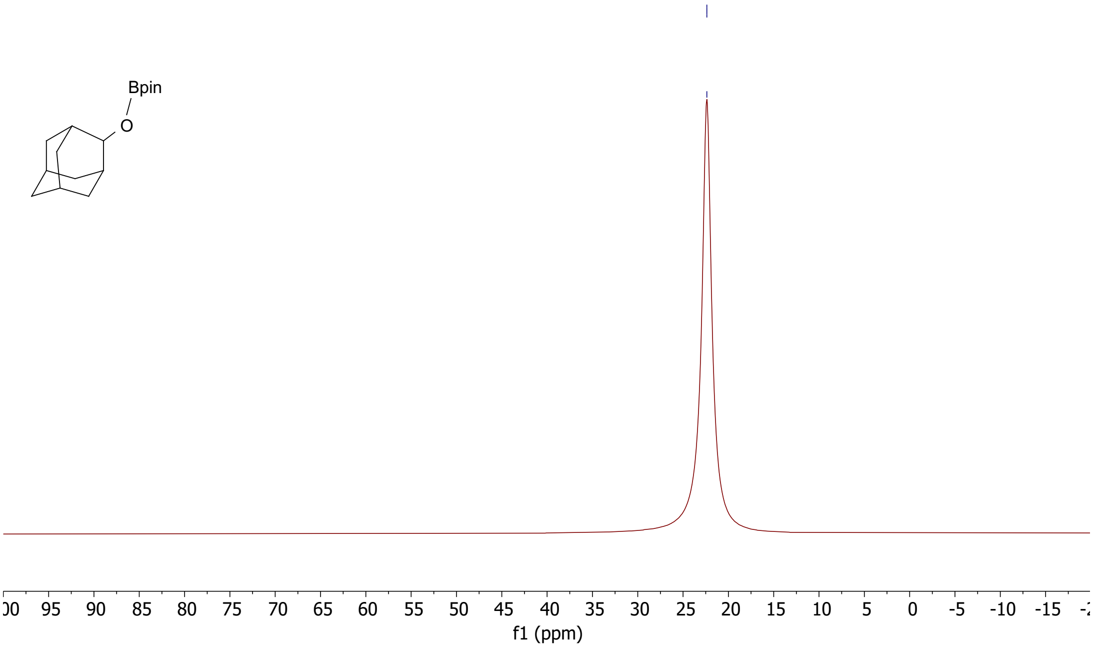

Figure S54: ^{11}B NMR spectrum (128 MHz, $CDCl_3$) of **2o**

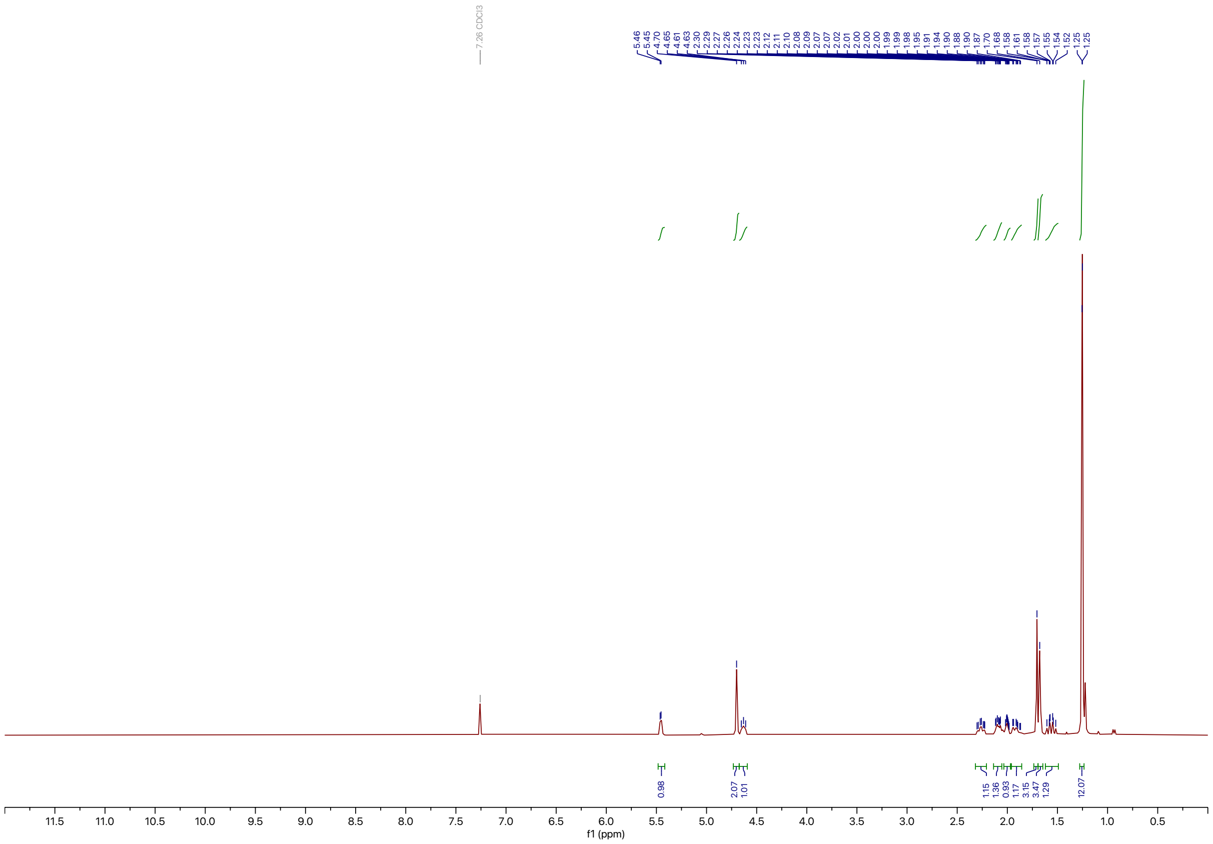

Figure S55. ^{1}H NMR spectrum (400 MHz, $CDCl_3$) of **2p**

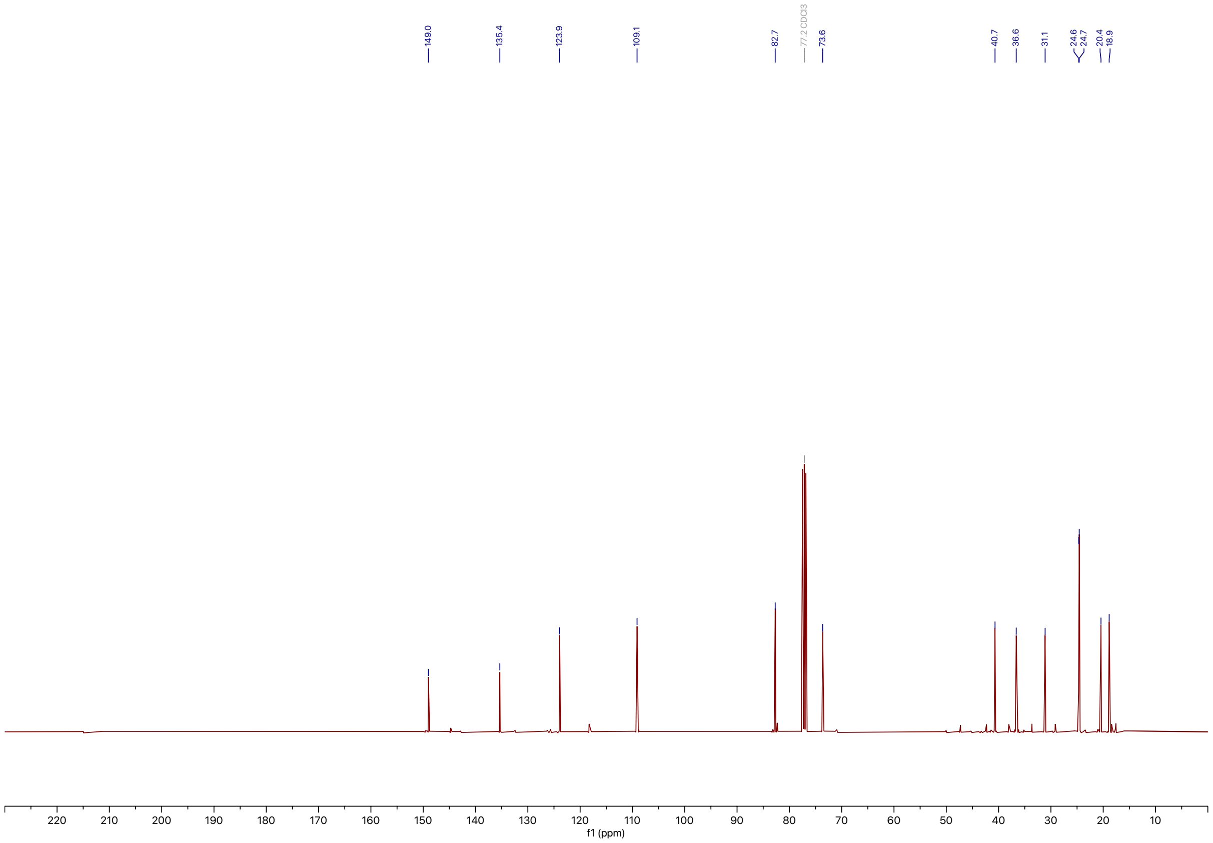

Figure S56. $^{13}C\{^{1}H\}$ NMR spectrum of (101 MHz, $CDCl_3$) of **2p**

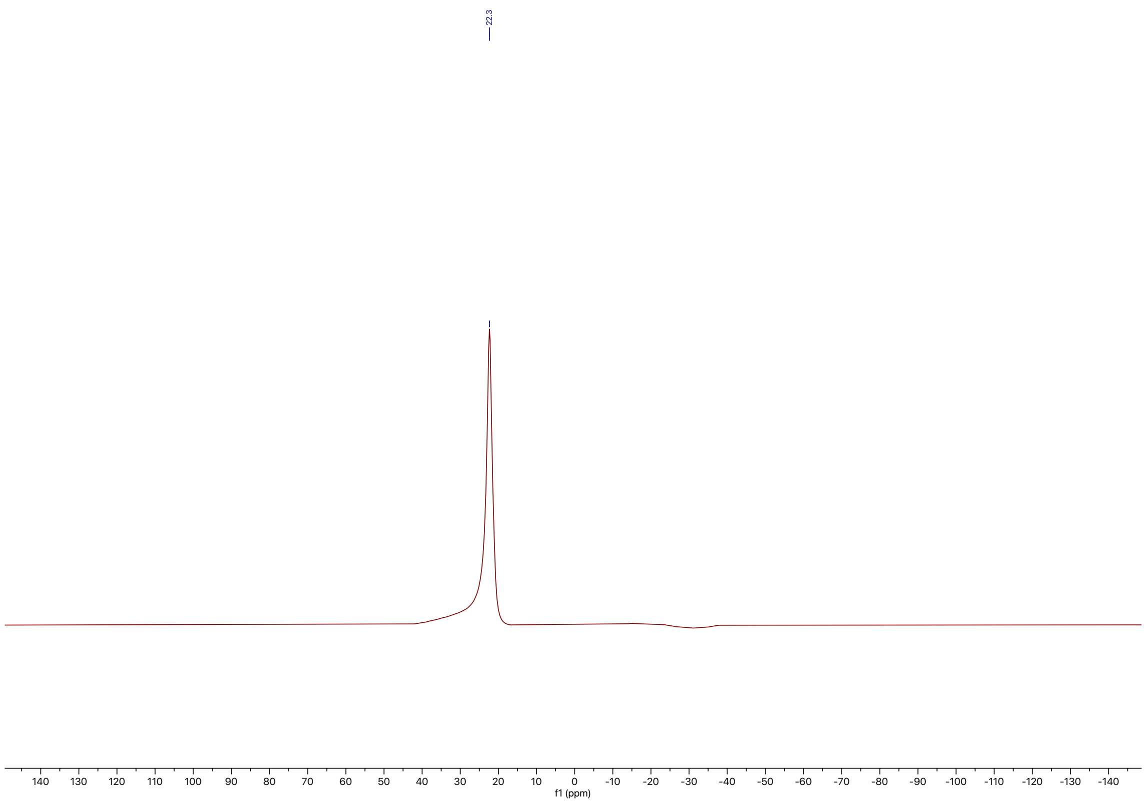

Figure S57.^{11}B NMR spectrum (128 MHz, $CDCl_3$) of **2p**

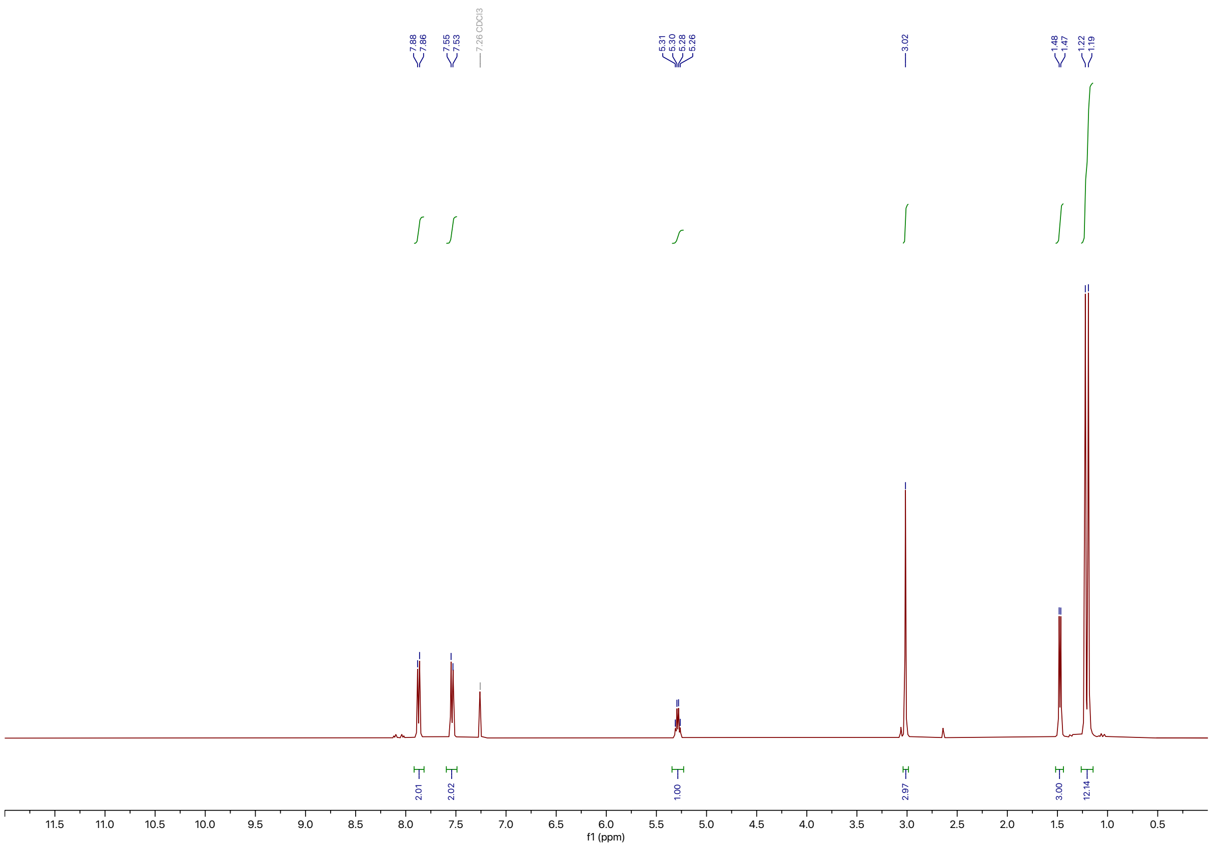

Figure S58. ^{1}H NMR spectrum (400 MHz, $CDCl_3$) of **2q**

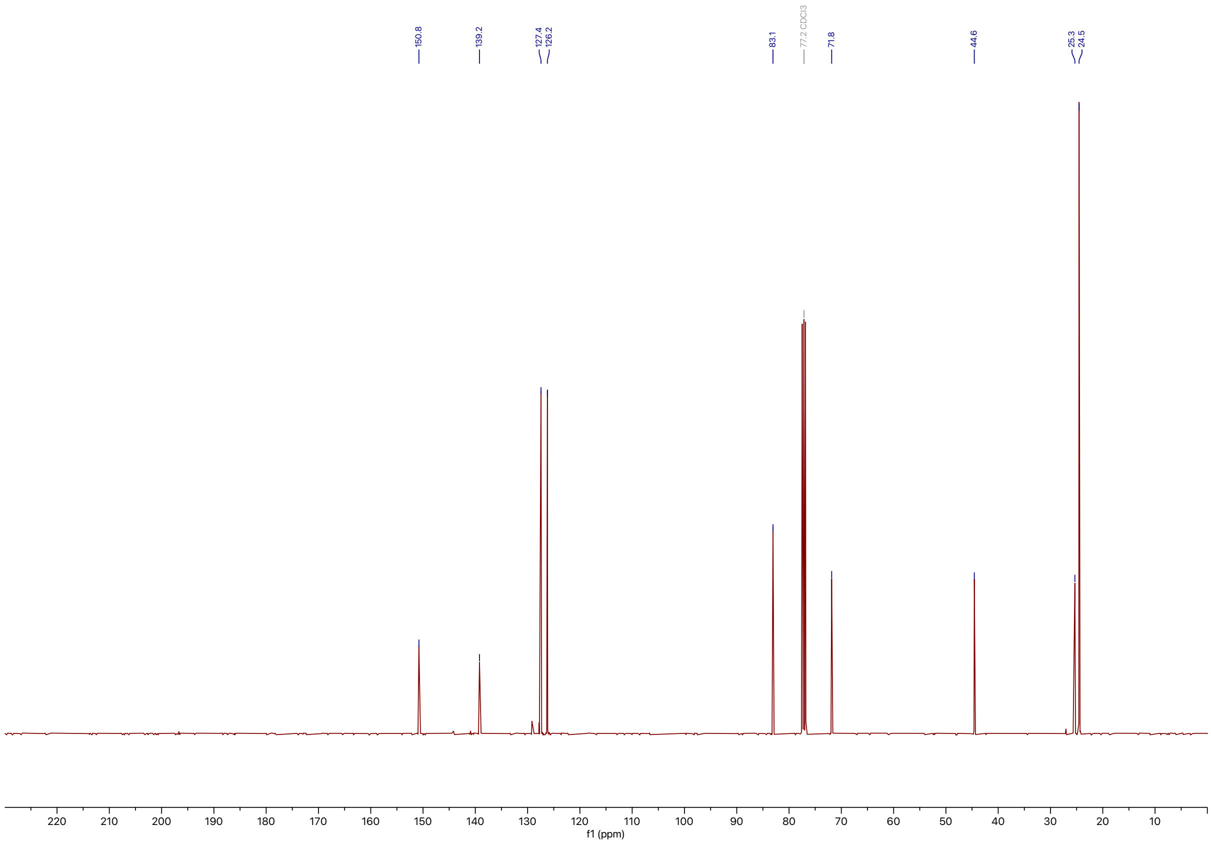

Figure S59. $^{13}C\{^1H\}$ NMR spectrum (101 MHz, $CDCl_3$) of **2q**

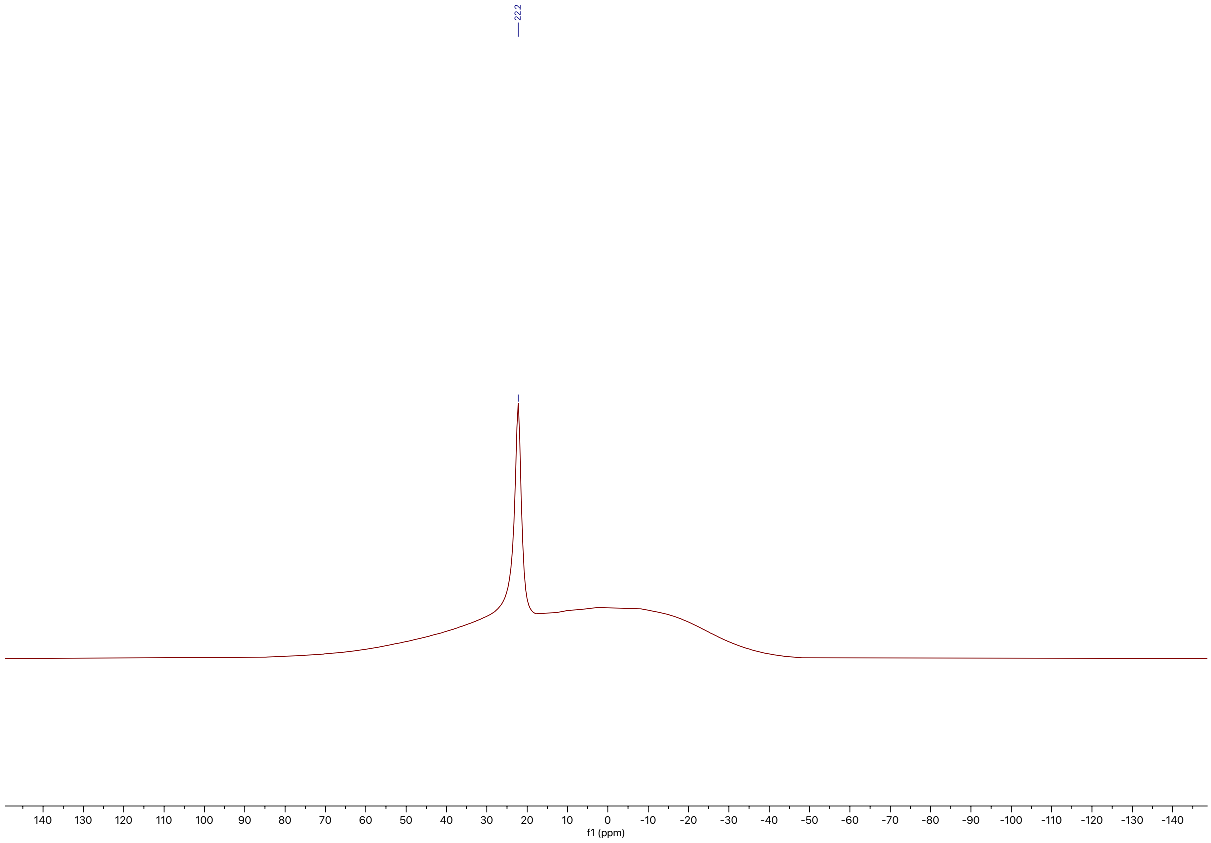

Figure S60. ^{11}B NMR spectrum (128 MHz, $CDCl_3$) of **2q**

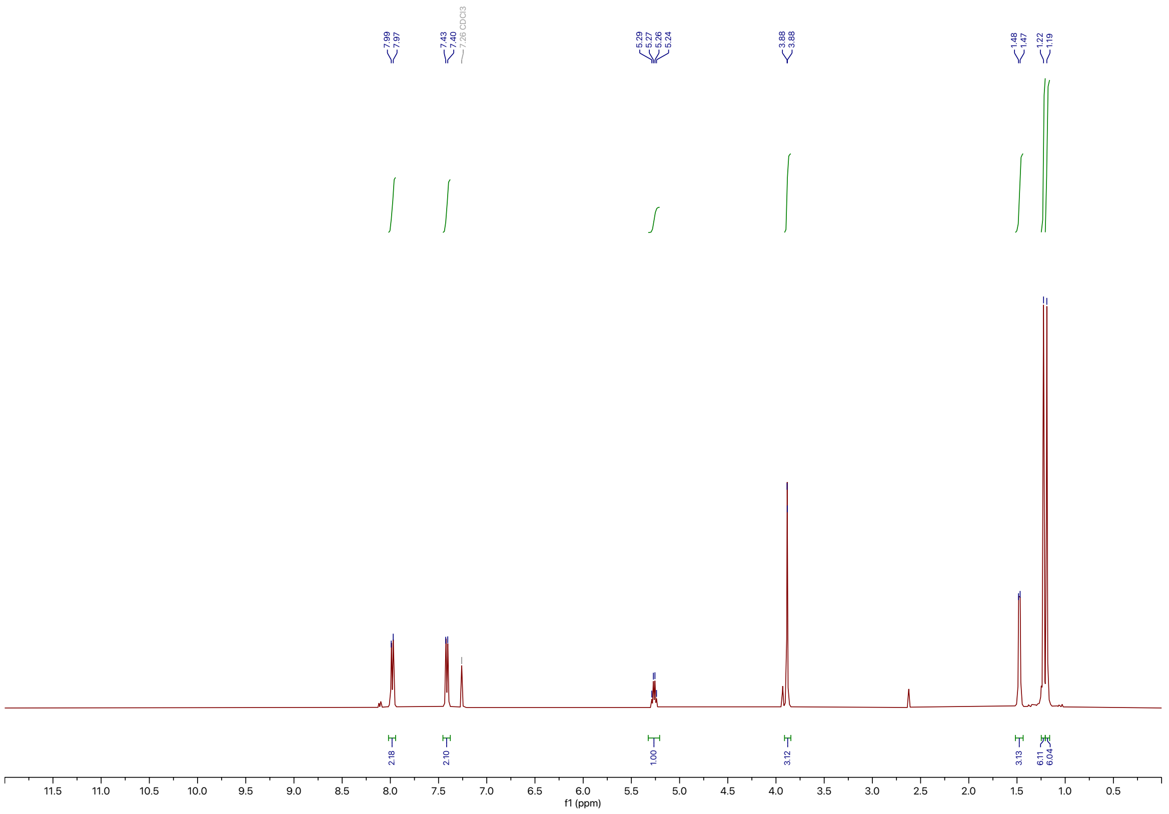

Figure S61. ^{1}H NMR spectrum (400 MHz, $CDCl_3$) of **2r**

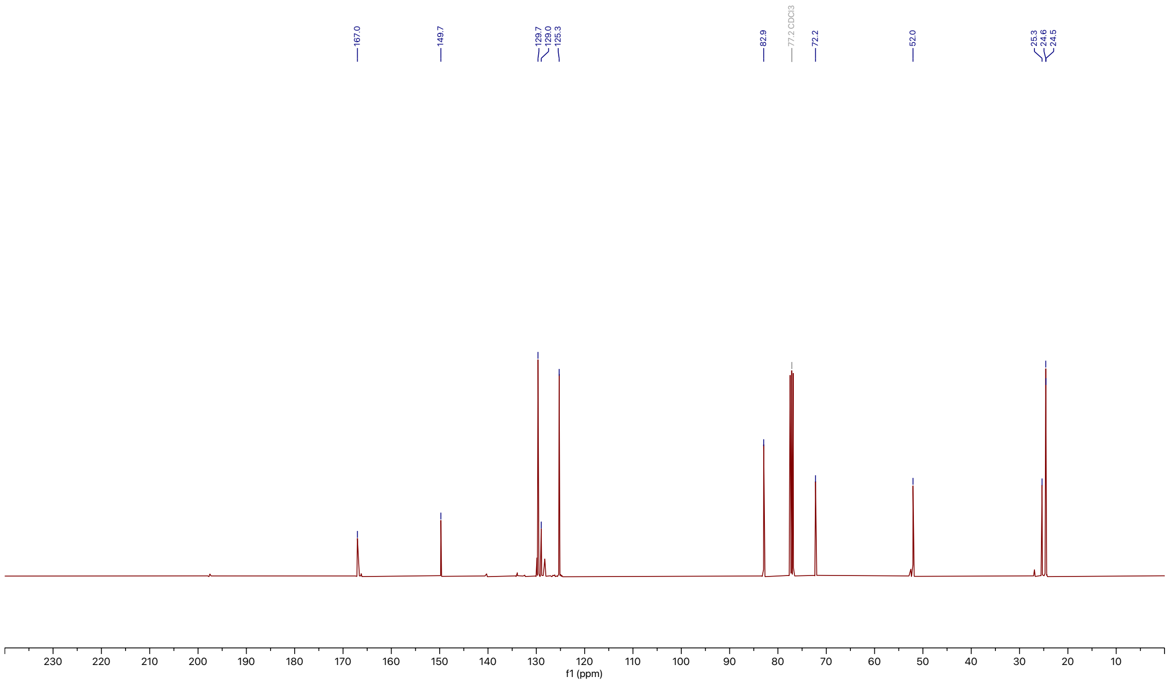

Figure S62. $^{13}C\{^{1}H\}$ NMR spectrum (101 MHz, $CDCl_3$) of **2r**

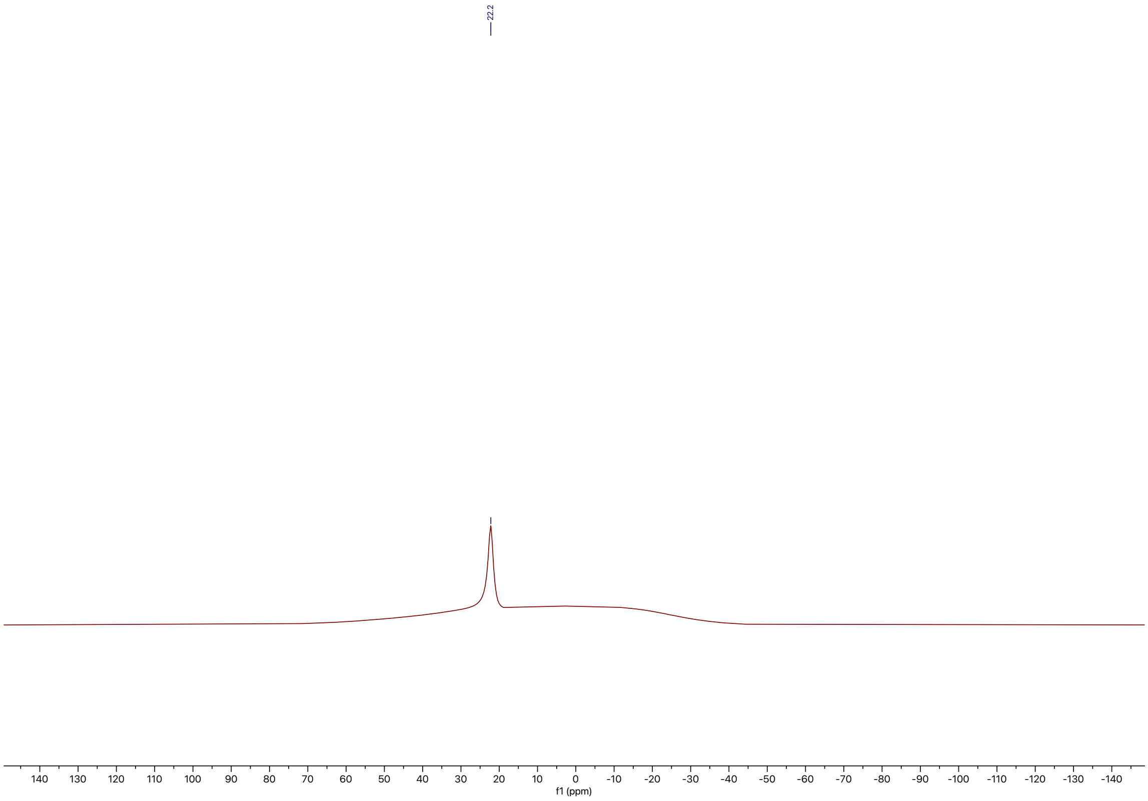

Figure S63. ^{11}B NMR spectrum (128 MHz, $CDCl_3$) of **2r**

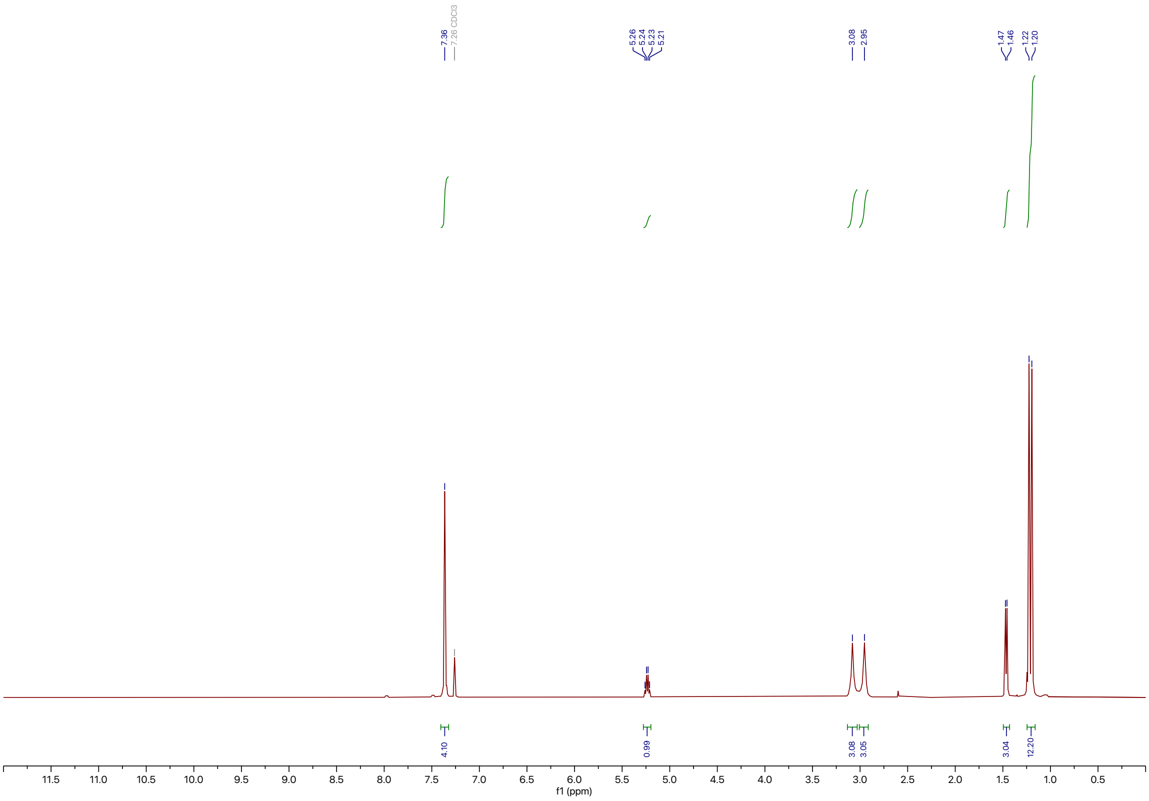

Figure S64. ^{1}H NMR spectrum (400 MHz, $CDCl_3$) of **2s**

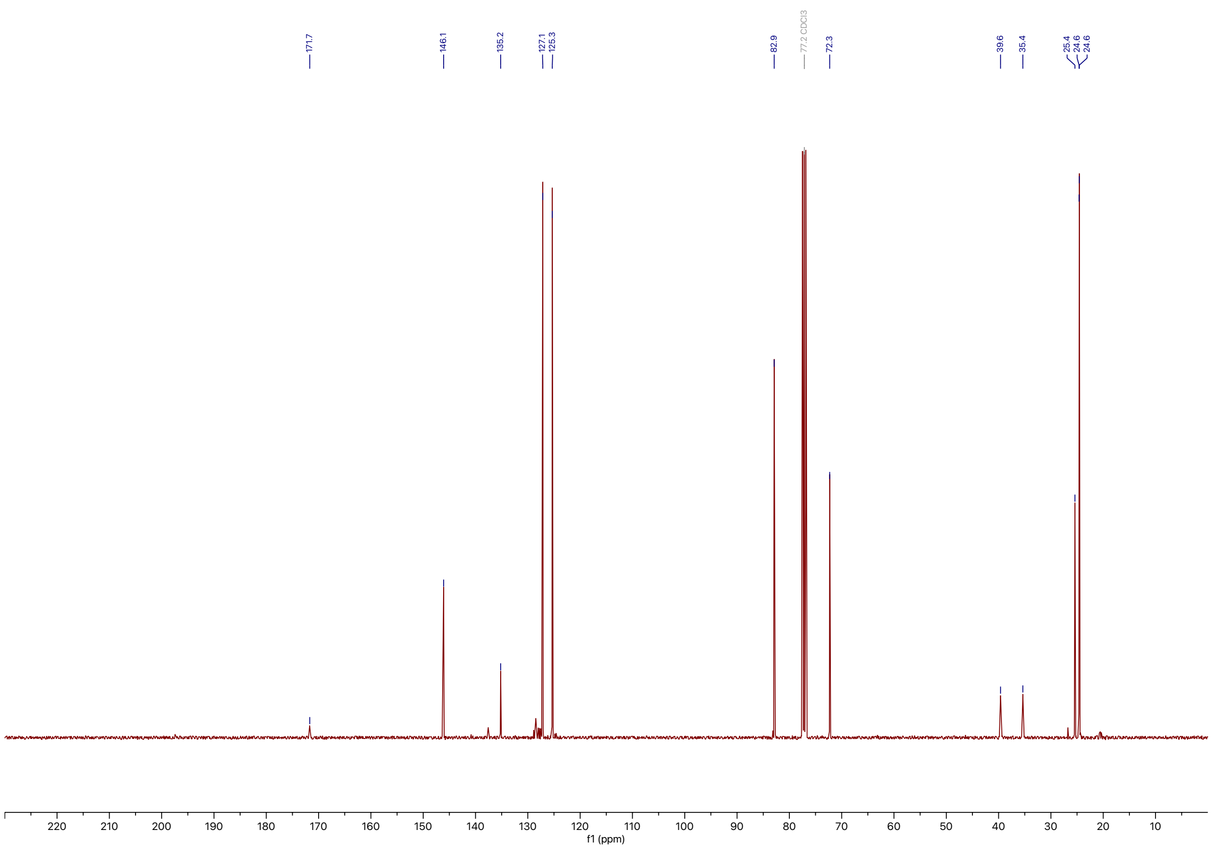

Figure S65. $^{13}C\{^{1}H\}$ NMR spectrum (101 MHz, $CDCl_3$) of **2s**

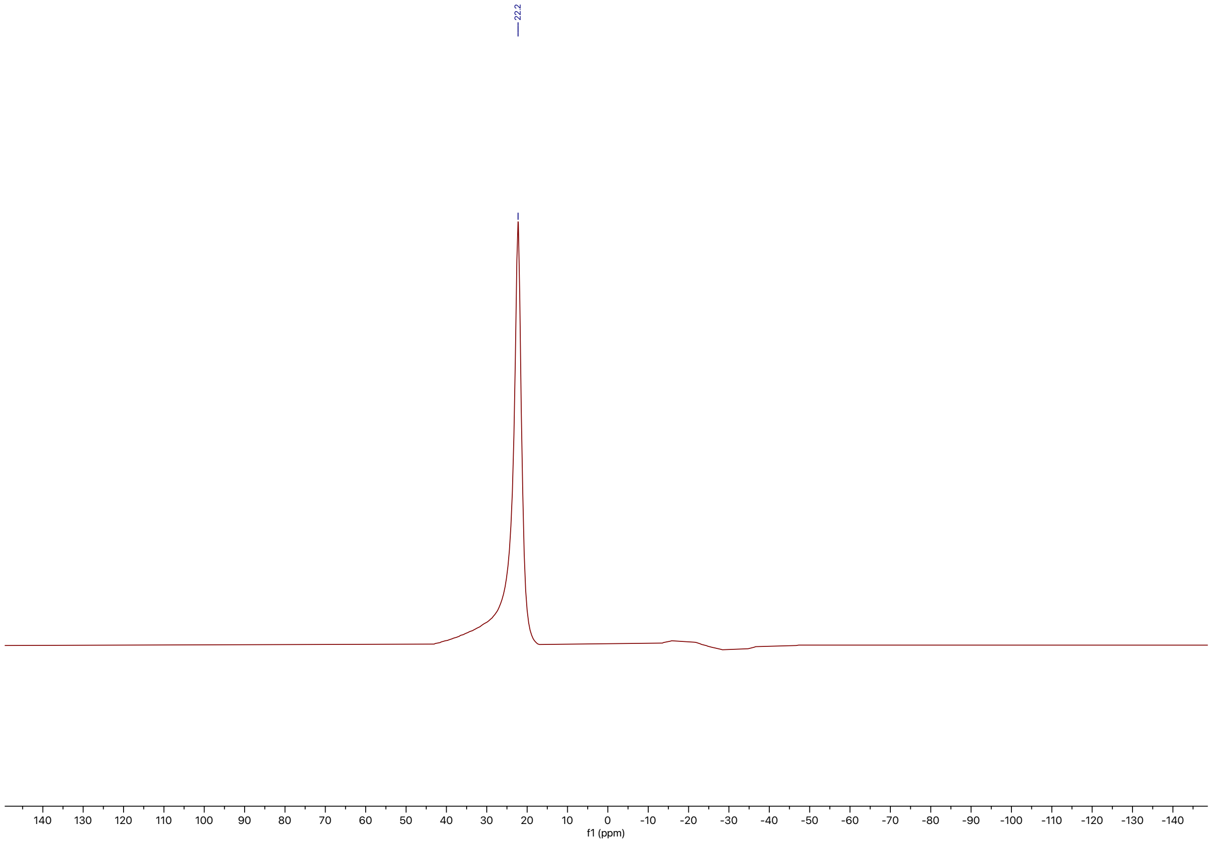

Figure S66. ^{11}B NMR spectrum (128 MHz, $CDCl_3$) of **2s**

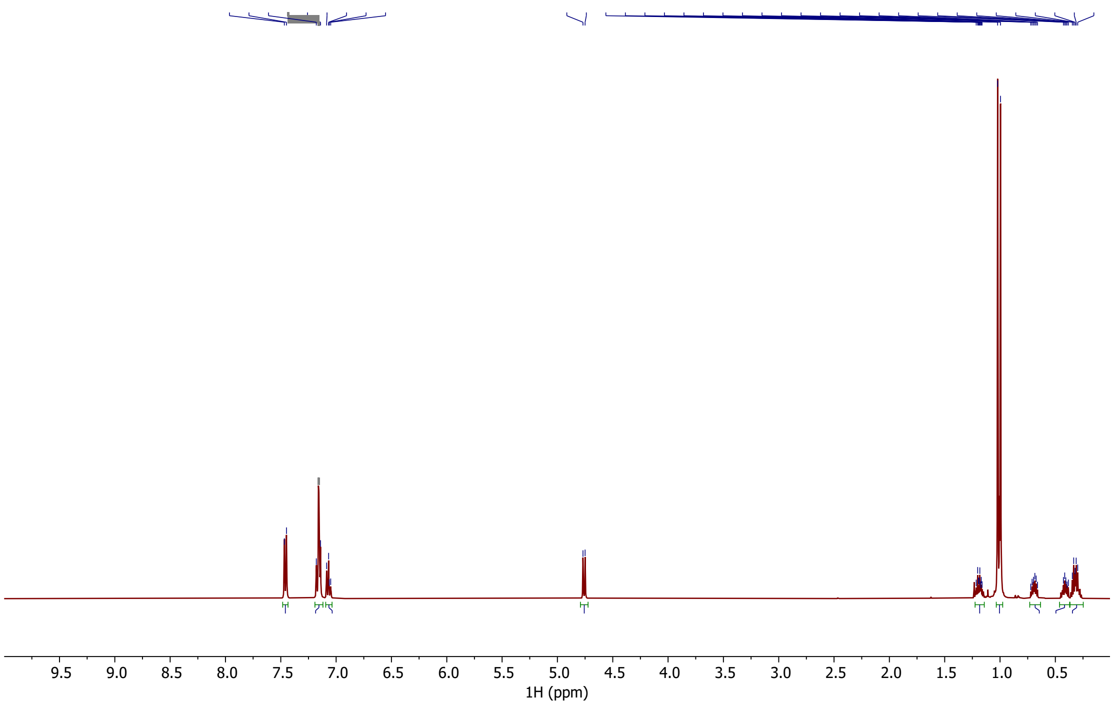

Figure S67: ^{1}H NMR spectrum (400 MHz, $CDCl_3$) of **2t**

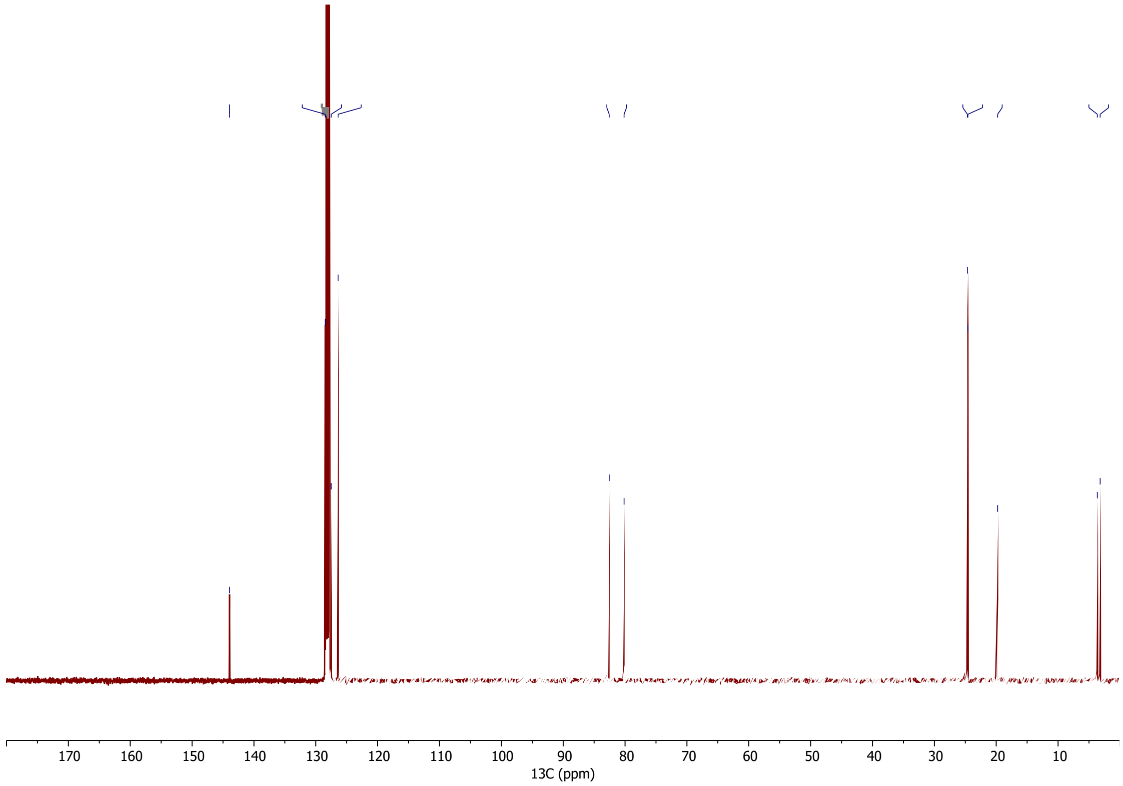

Figure S68: ^{13}C NMR spectrum (101 MHz, $CDCl_3$) of **2t**

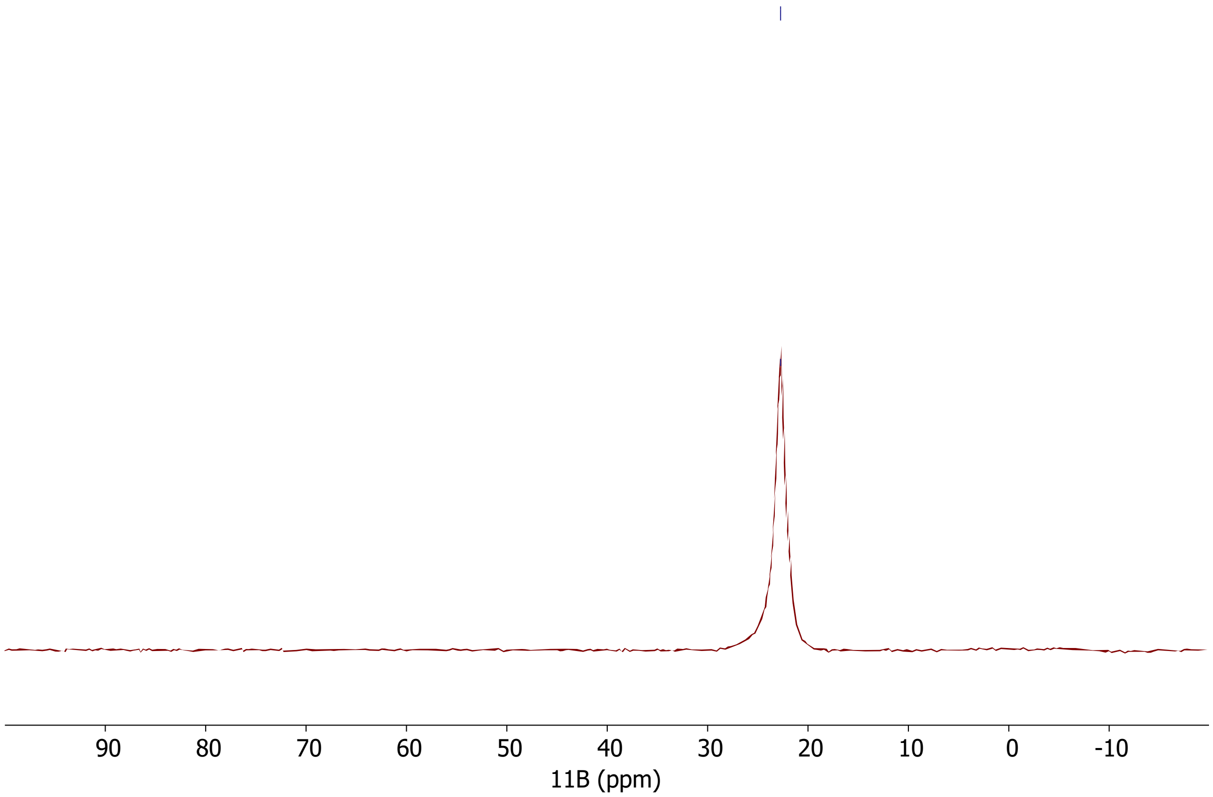

Figure S69: ^{11}B NMR spectrum (128 MHz, $CDCl_3$) of **2t**

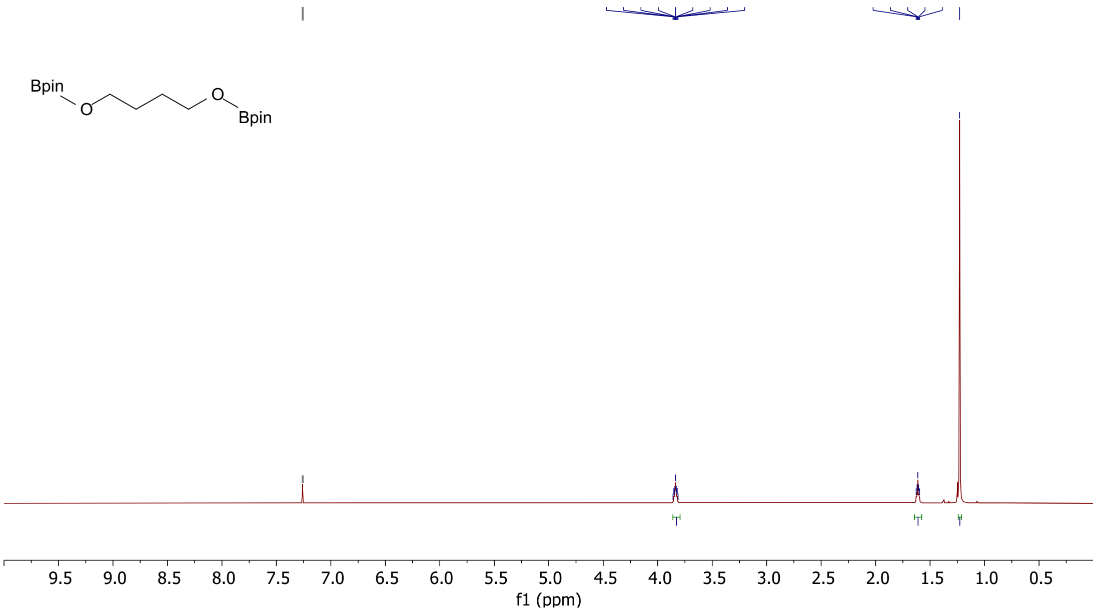

Figure S70: ^{1}H NMR spectrum (400 MHz, $CDCl_3$) of **4a**

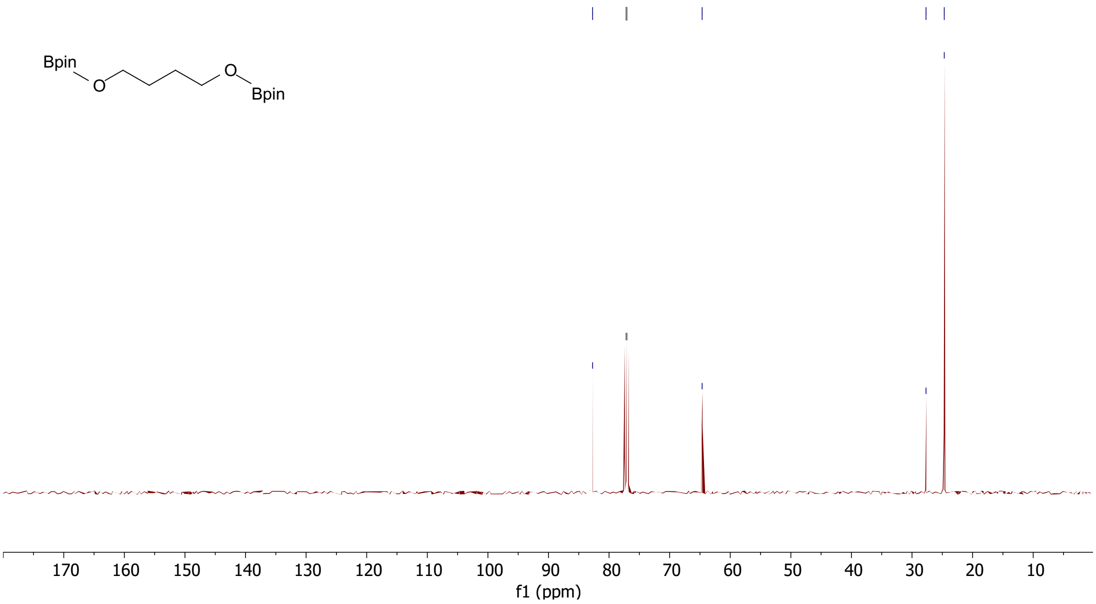

Figure S71: $^{13}C\{^{1}H\}$ NMR spectrum (101 MHz, $CDCl_3$) of **4a**

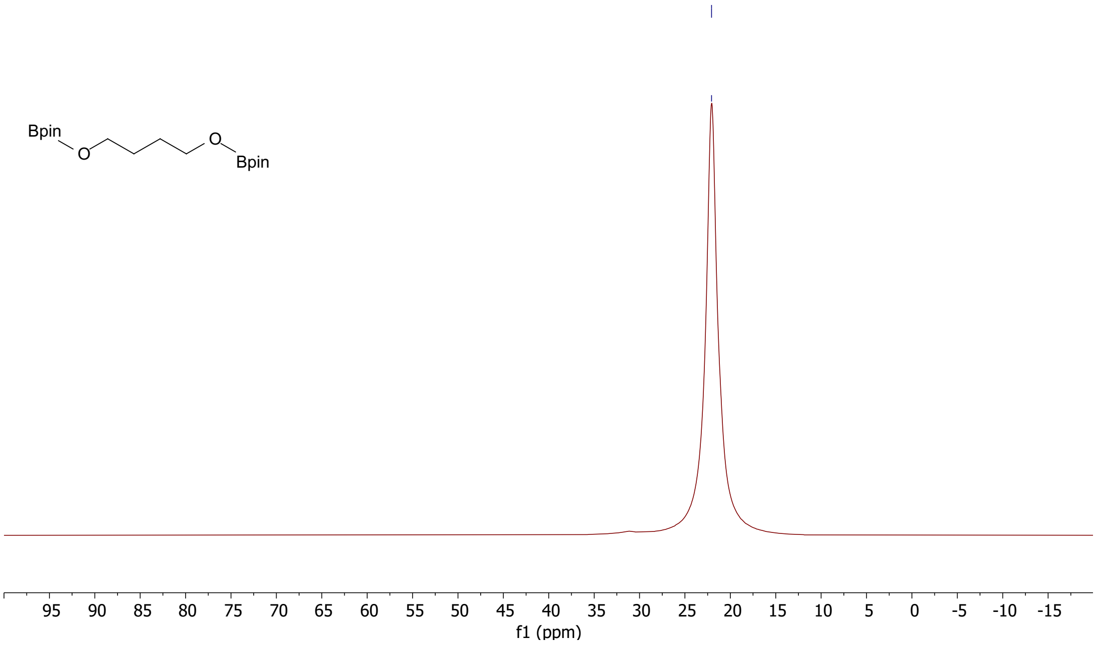

Figure S72: ^{11}B NMR spectrum (128 MHz, $CDCl_3$) of **4a**

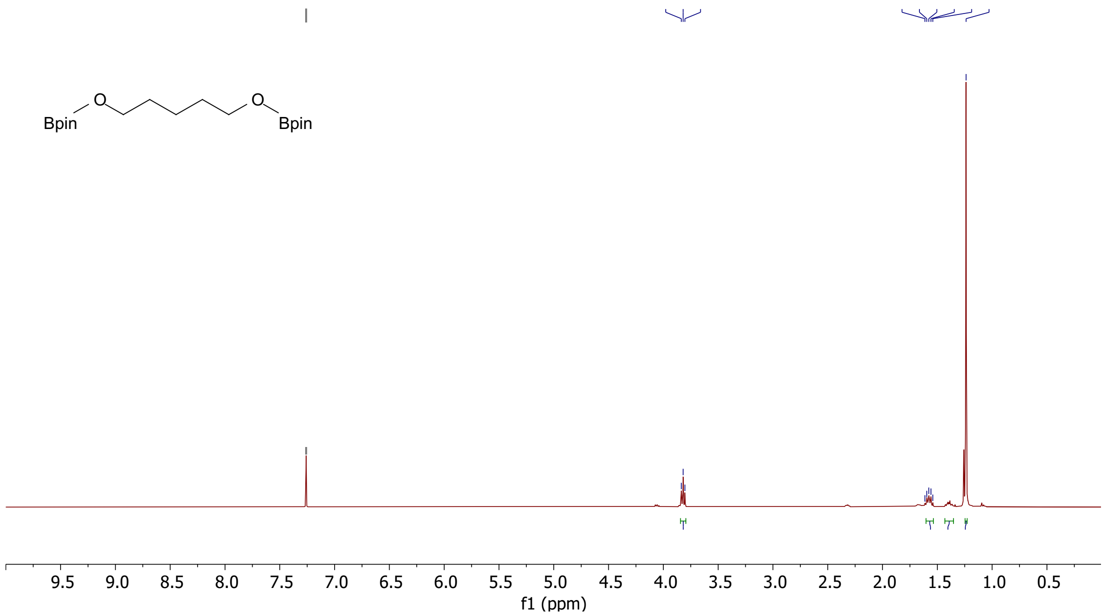

Figure S73: ^{1}H NMR spectrum (400 MHz, $CDCl_3$) of **4b**

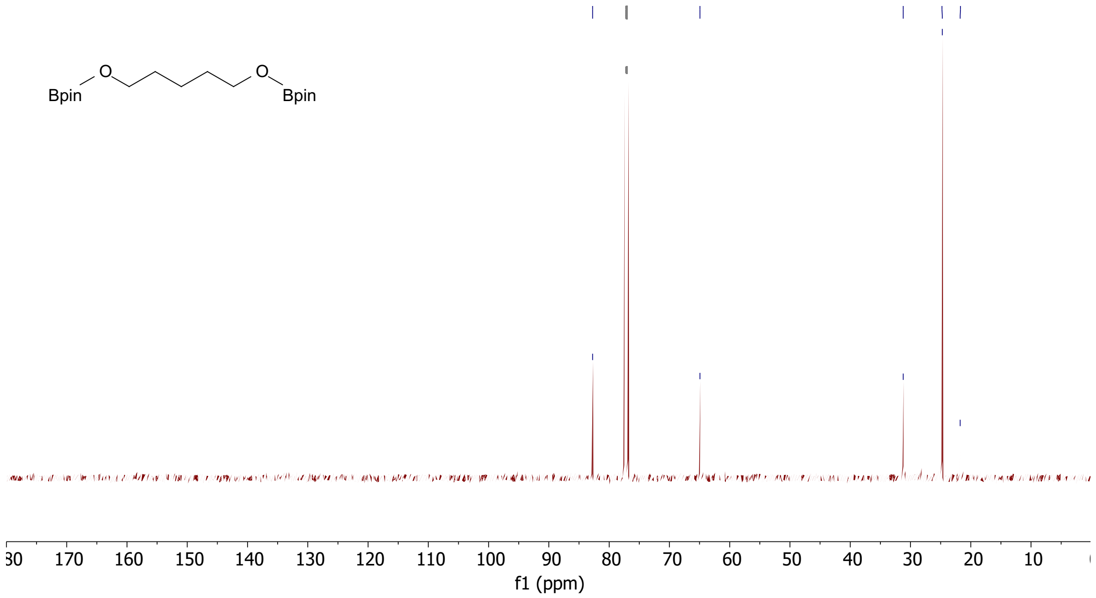

Figure S74: $^{13}C\{^{1}H\}$ NMR spectrum (101 MHz, $CDCl_3$) of **4b**

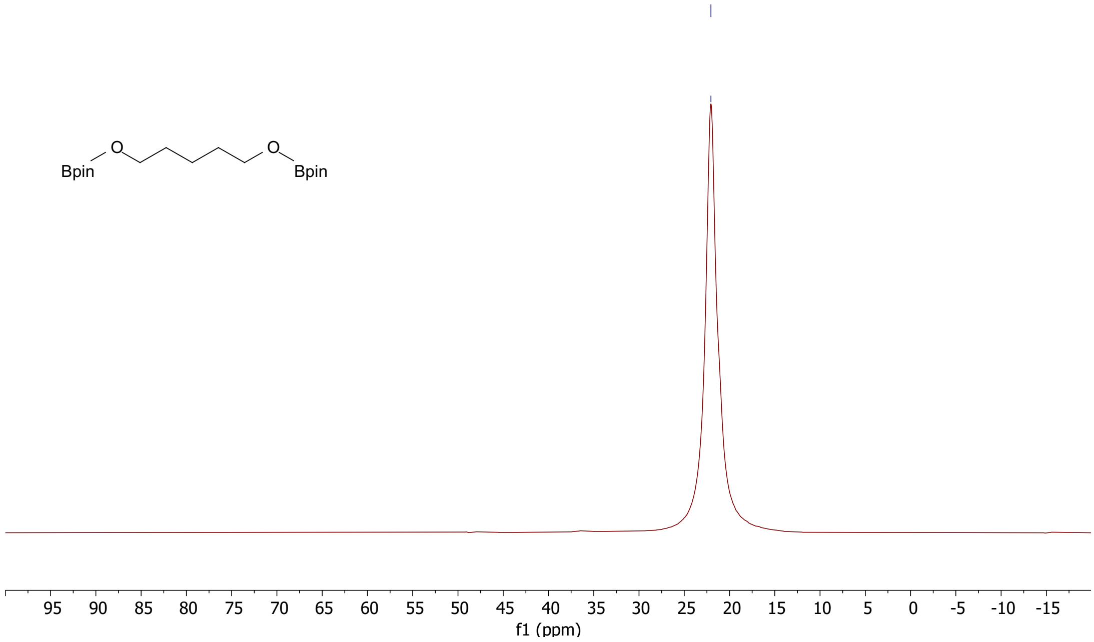

Figure S75: ^{11}B NMR spectrum (128 MHz, $CDCl_3$) of **4b**

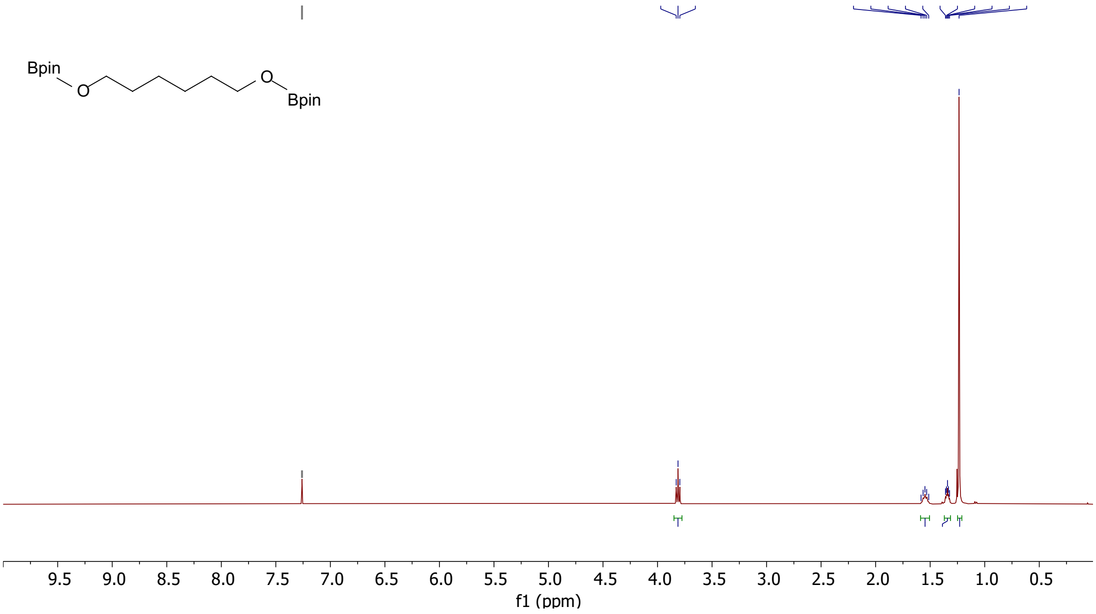

Figure S76: ^{1}H NMR spectrum (400 MHz, $CDCl_3$) of **4c**

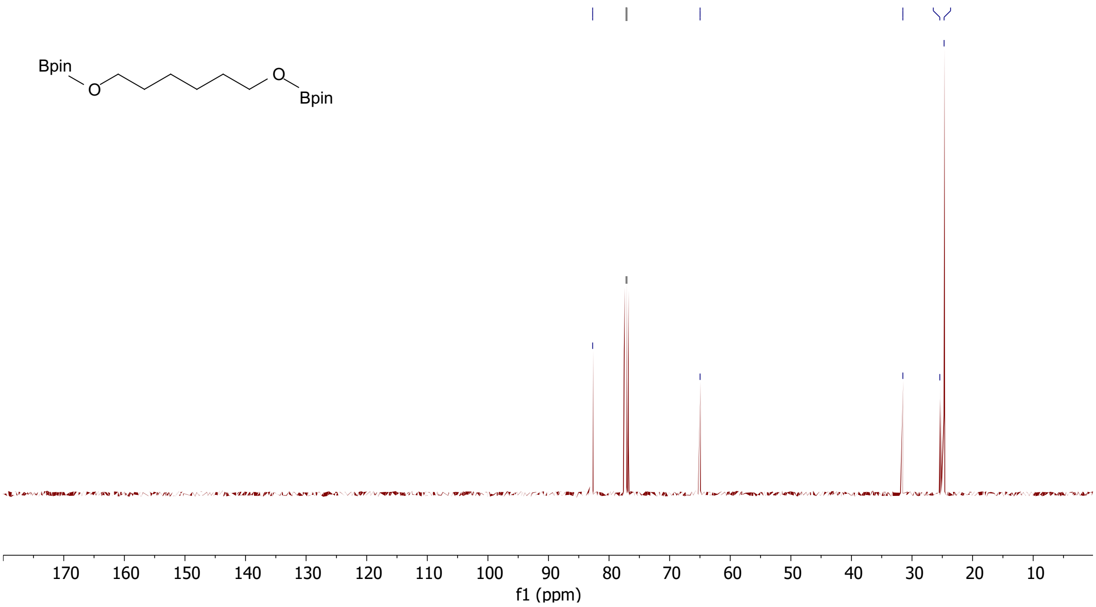

Figure S77: $^{13}C\{^{1}H\}$ NMR spectrum (101 MHz, $CDCl_3$) of **4c**

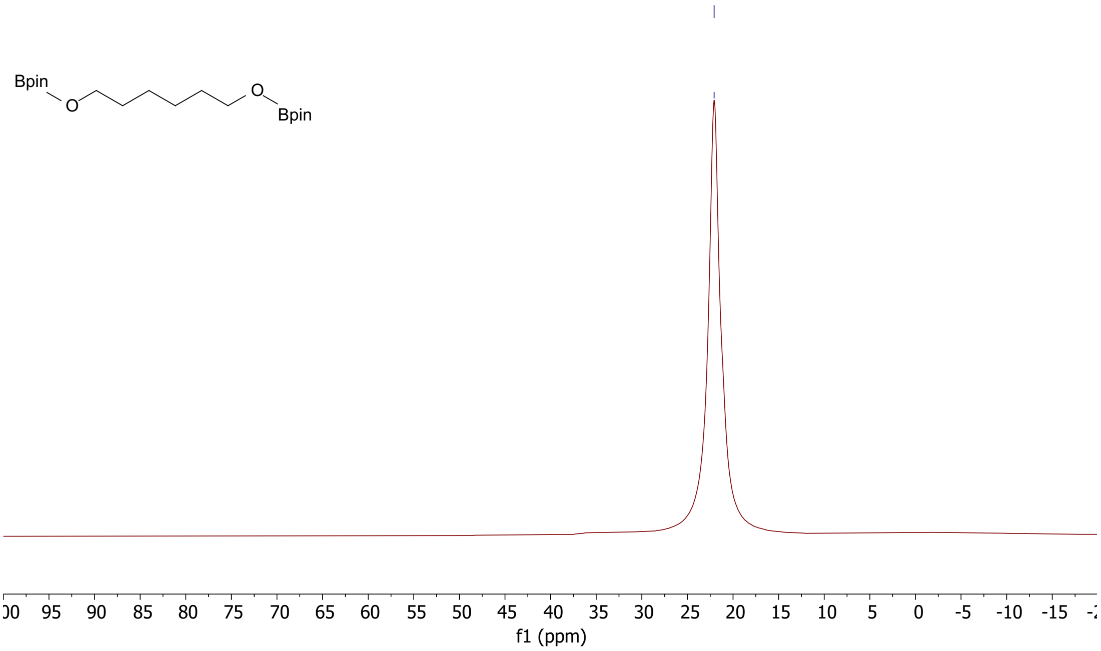

Figure S78: ^{11}B NMR spectrum (128 MHz, $CDCl_3$) of **4c**

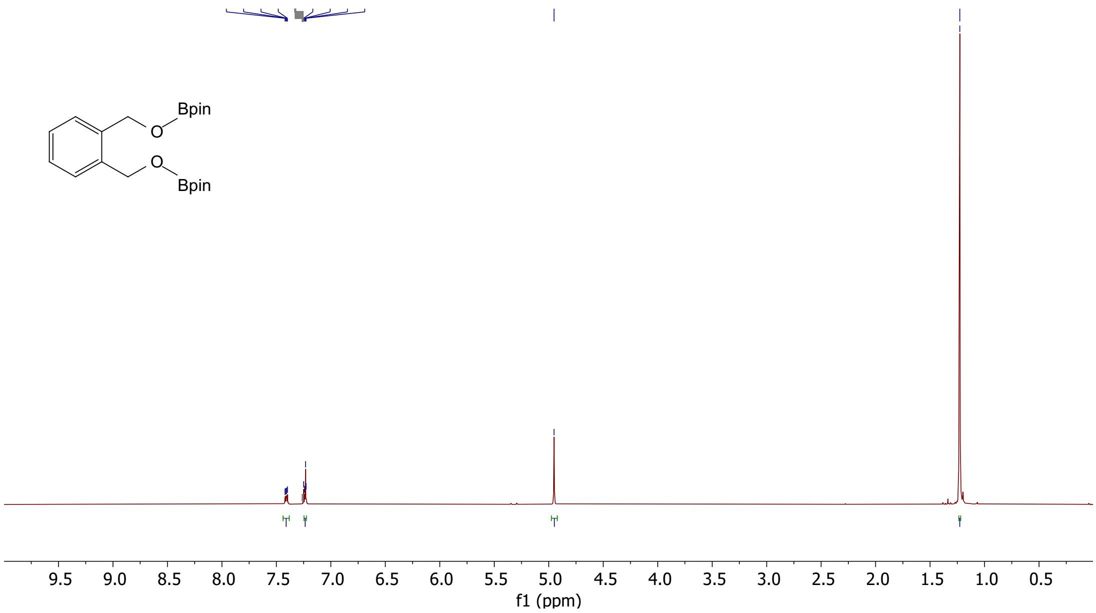

Figure S79: ^{1}H NMR spectrum (400 MHz, $CDCl_3$) of **4d**

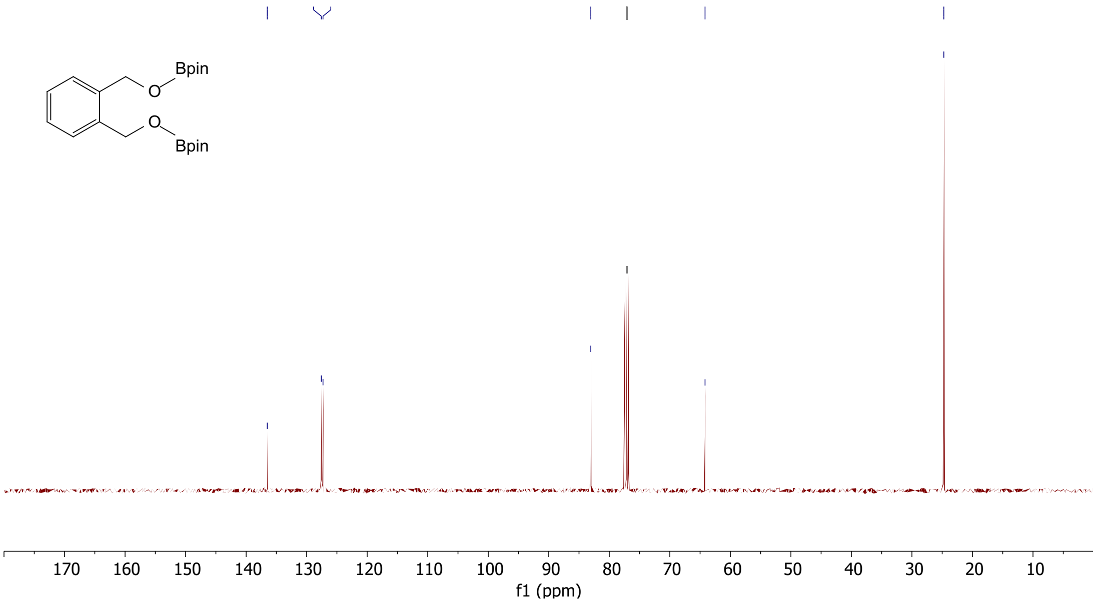

Figure S80: $^{13}C\{^{1}H\}$ NMR spectrum (101 MHz, $CDCl_3$) of **4d**

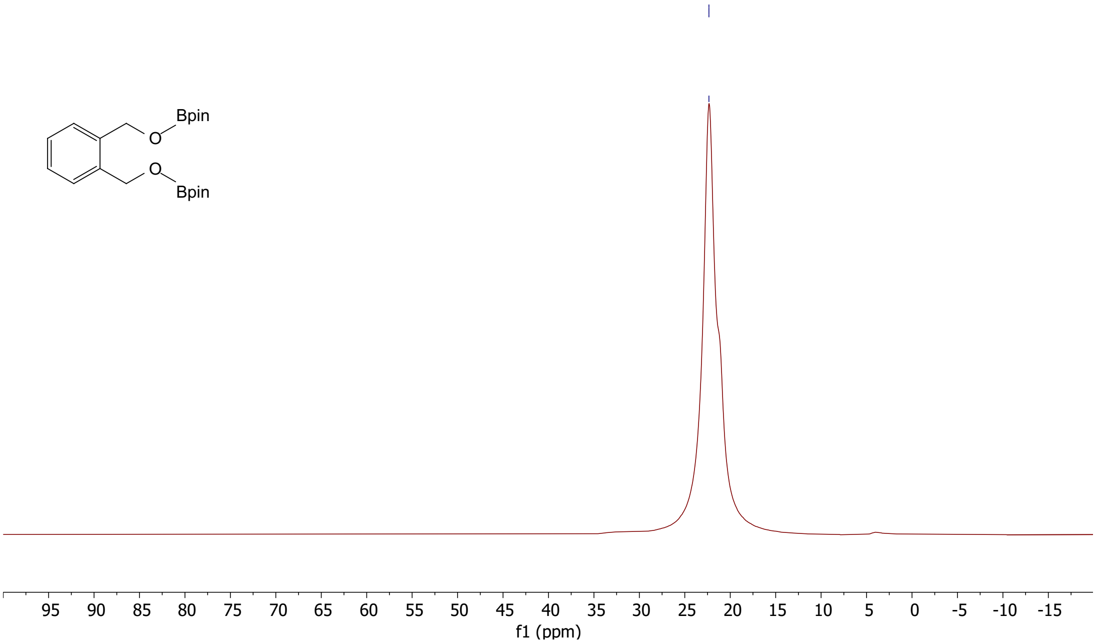

Figure S81: ^{11}B NMR spectrum (128 MHz, $CDCl_3$) of **4d**

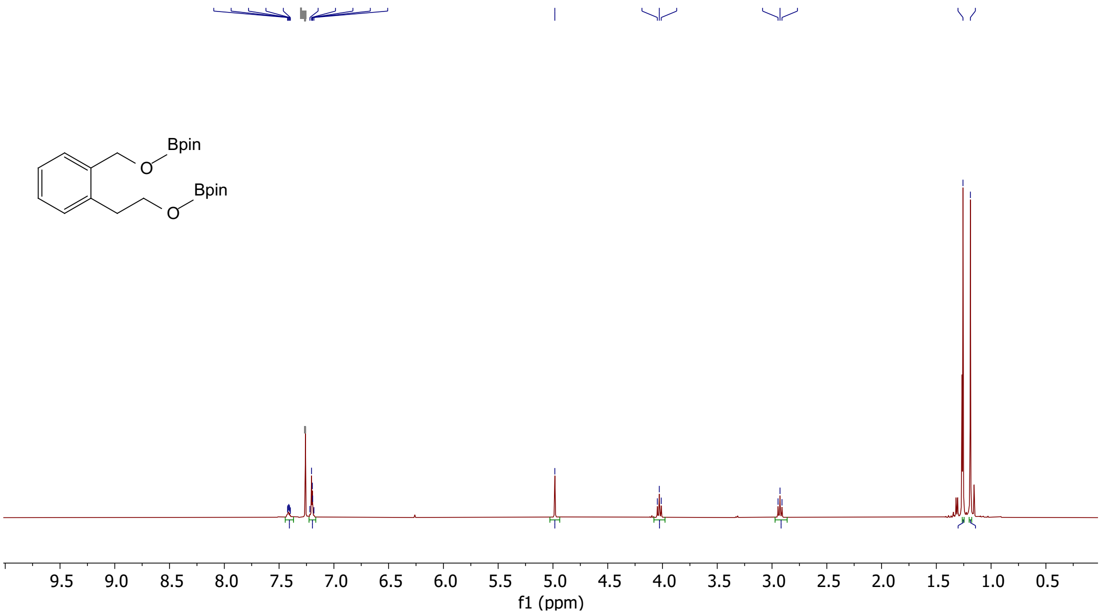

Figure S82: ^{1}H NMR spectrum (400 MHz, $CDCl_3$) of **4e**

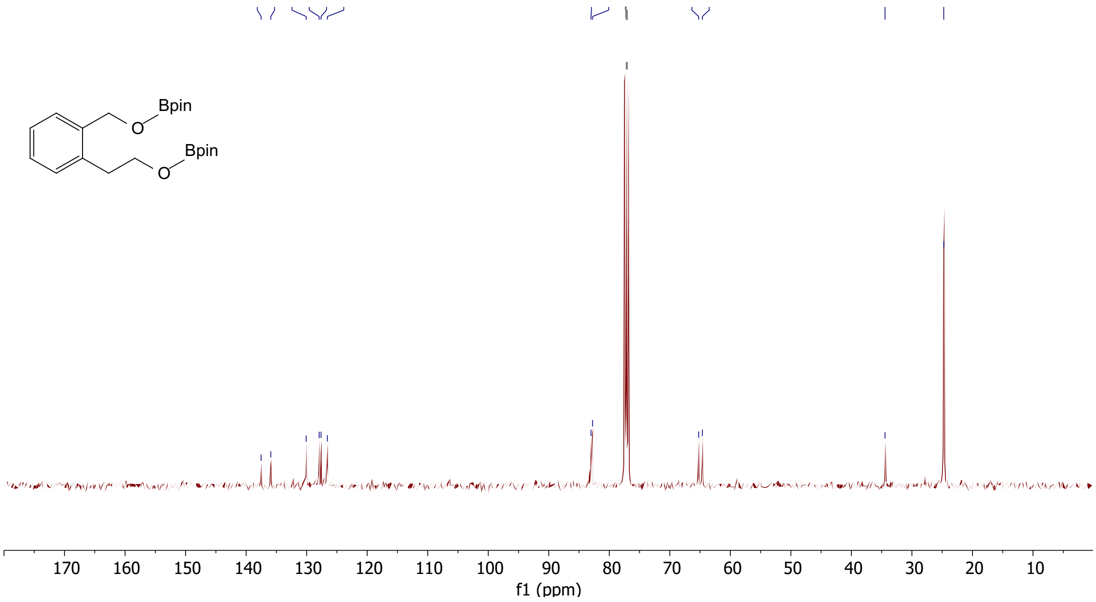

Figure S83: $^{13}C\{^{1}H\}$ NMR spectrum (101 MHz, $CDCl_3$) of **4e**

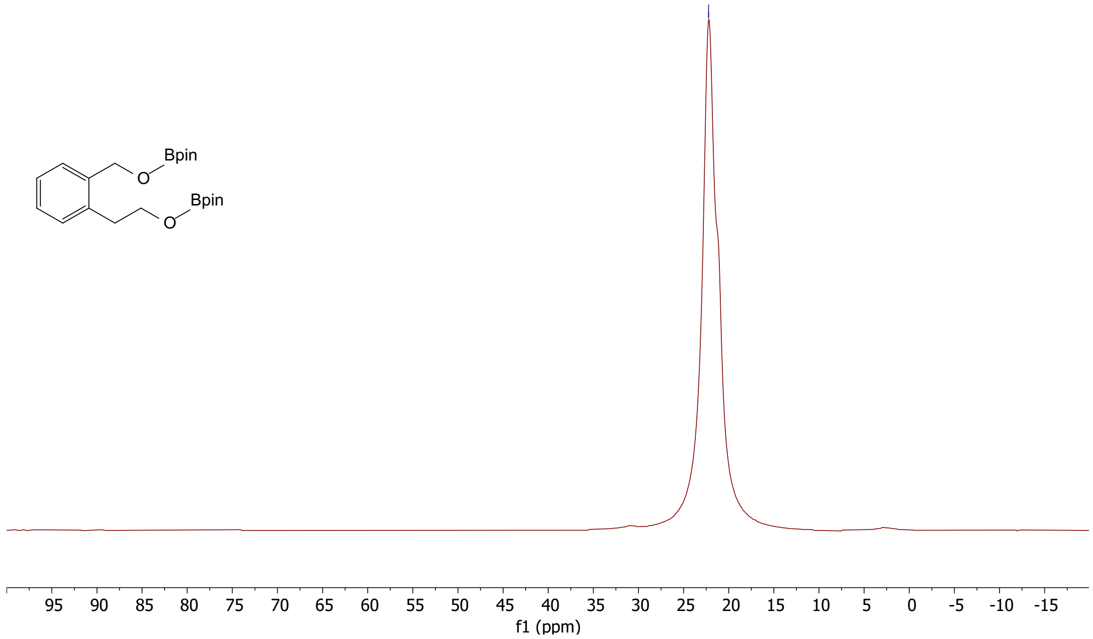

Figure S84: ^{11}B NMR spectrum (128 MHz, $CDCl_3$) of **4e**

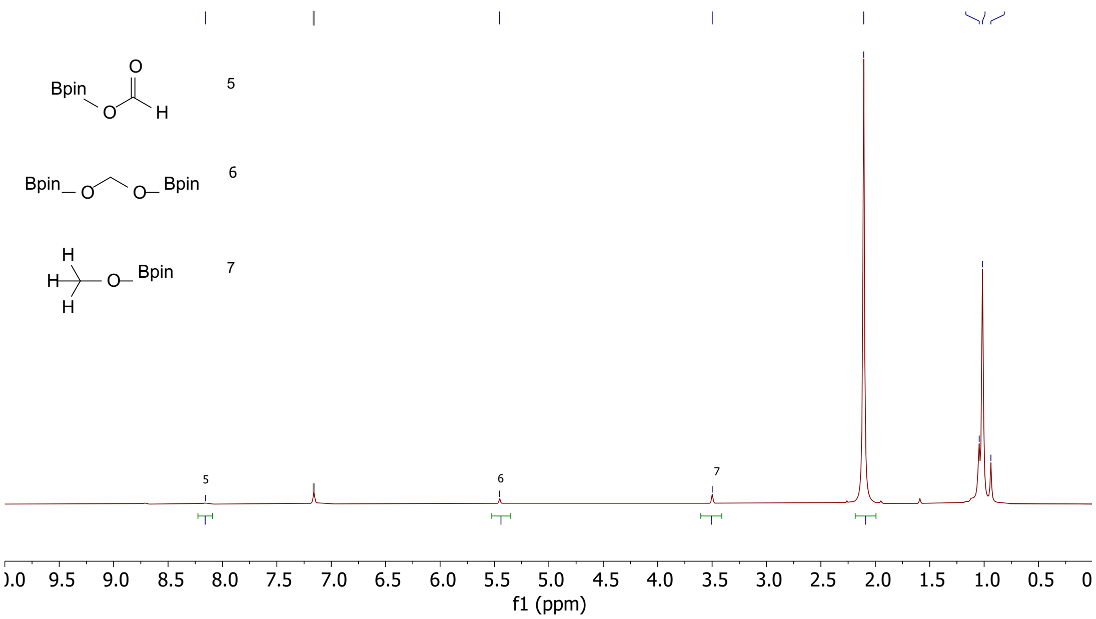

Figure S85: ^{1}H NMR spectrum (400 MHz, C_6D_6) entry 1 CO_2 reaction

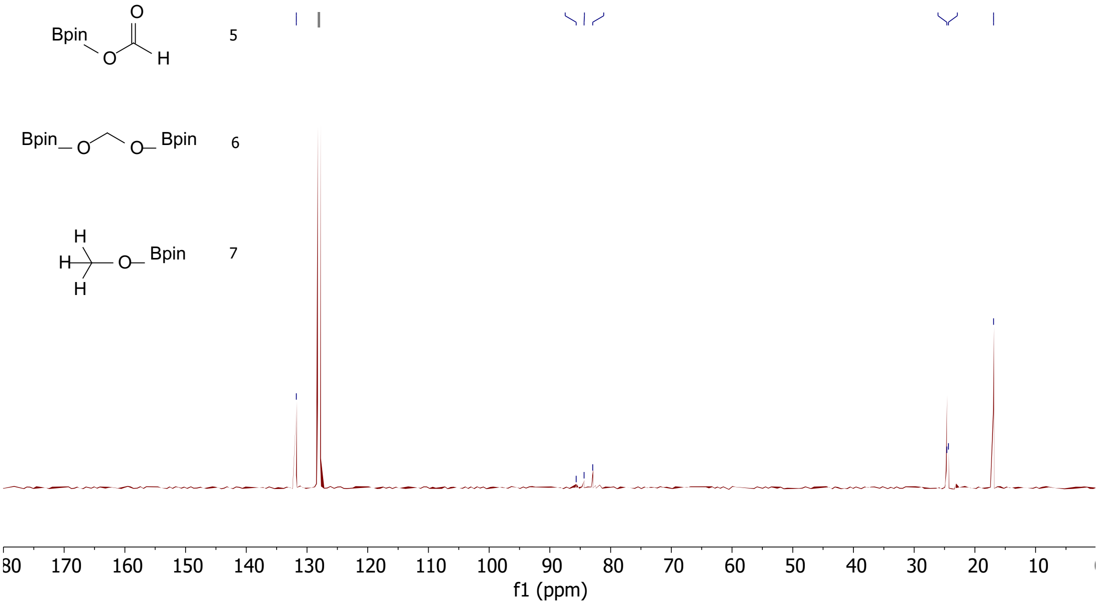

Figure S86: $^{13}C\{^{1}H\}$ NMR spectrum (101 MHz, C_6D_6) entry 1 CO_2 reaction

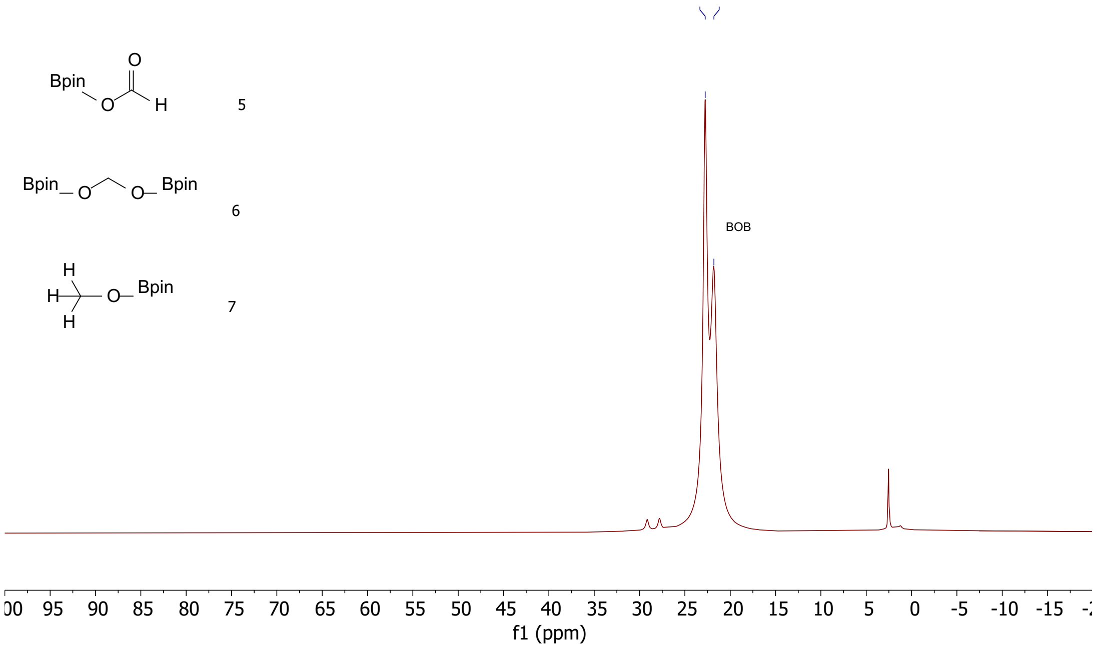

Figure S87: ^{11}B NMR spectrum (128 MHz, C_6D_6) entry 1 CO_2 reaction

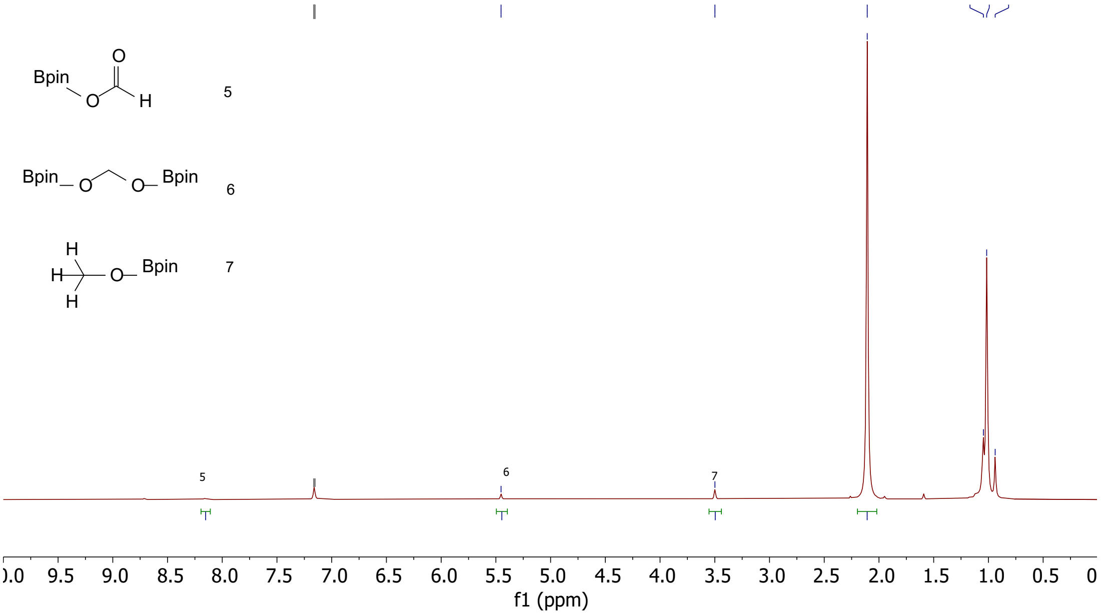

Figure S88: ^{1}H NMR spectrum (400 MHz, C_6D_6) entry 2 CO_2 reaction

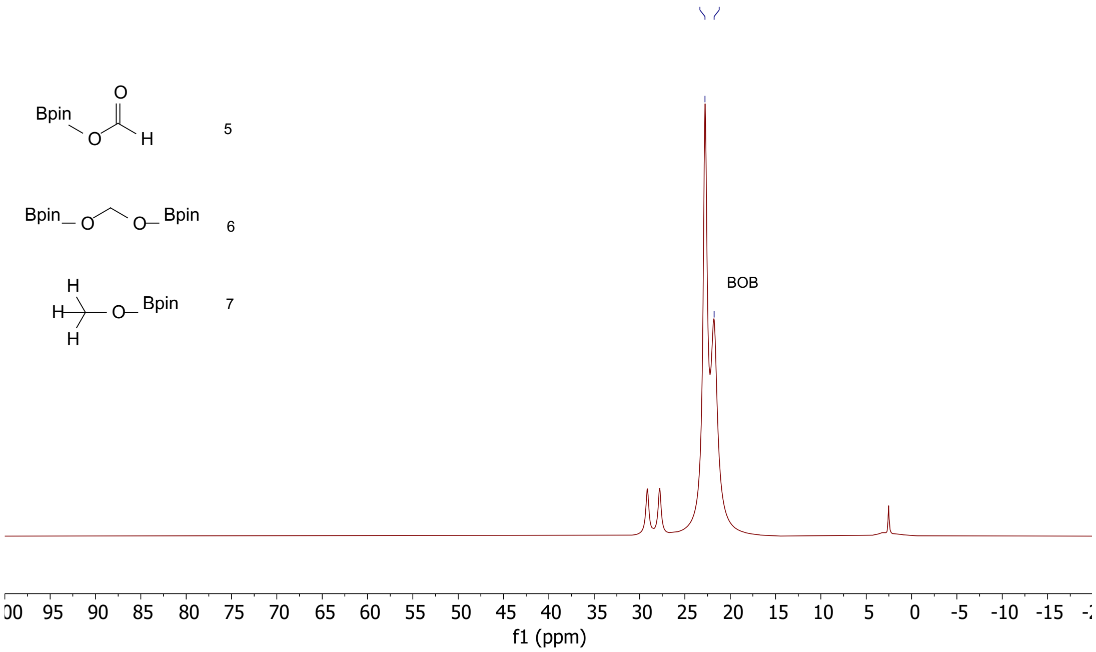

Figure S89: ^{11}B NMR spectrum (128 MHz, C_6D_6) entry 2 CO_2 reaction

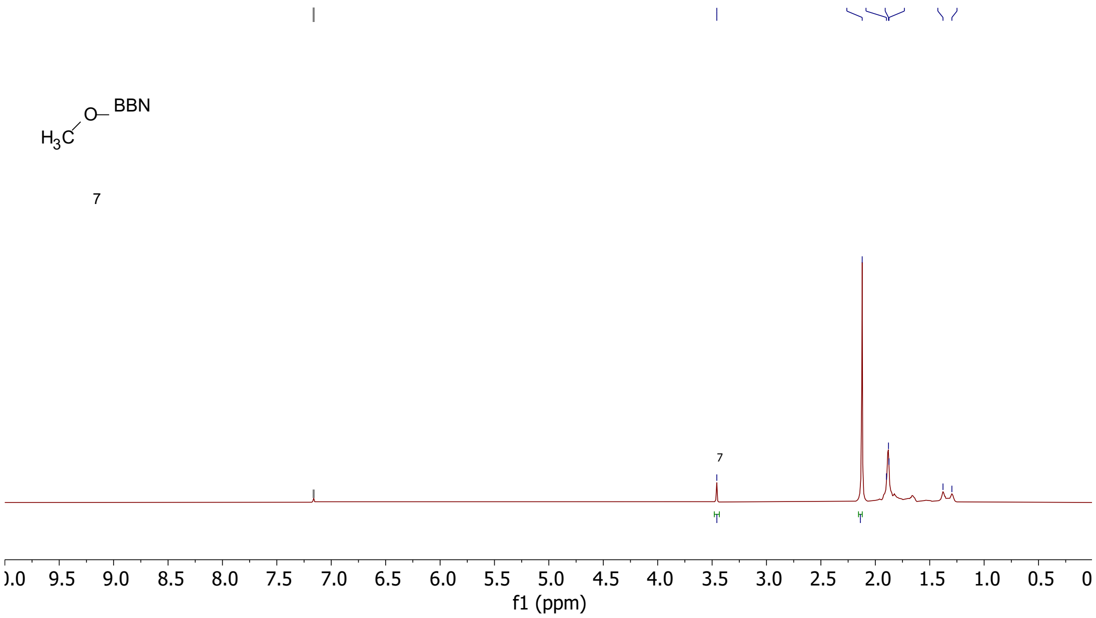

Figure S90: ^{1}H NMR spectrum (400 MHz, C_6D_6) entry 3 CO_2 reaction

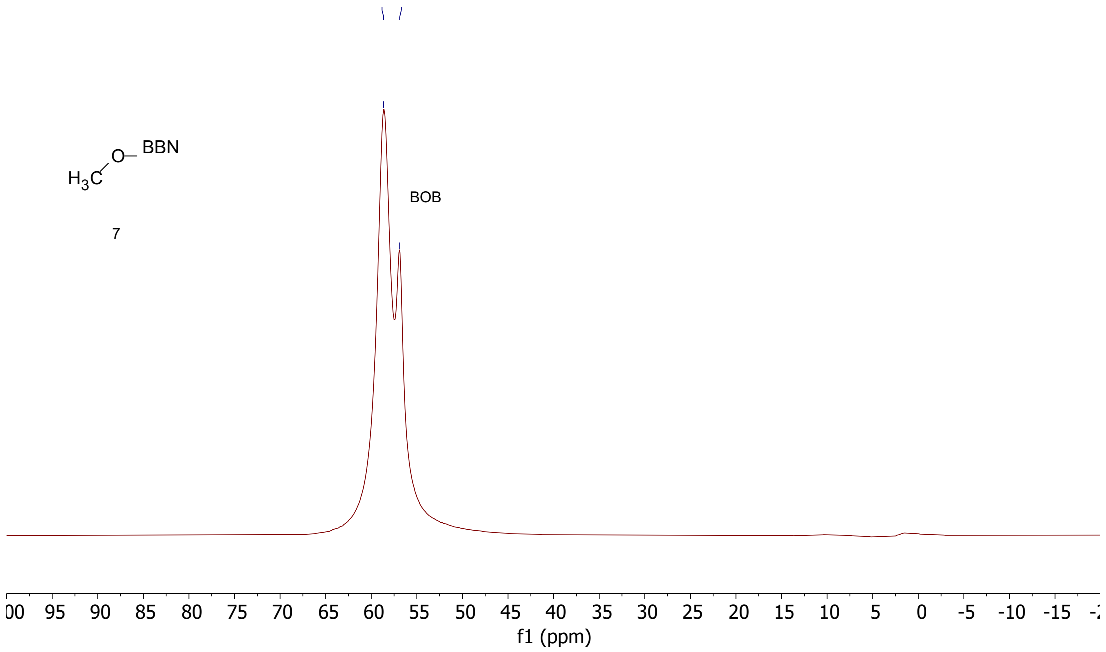

Figure S91: ^{11}B NMR spectrum (128 MHz, C_6D_6) entry 3 CO_2 reaction

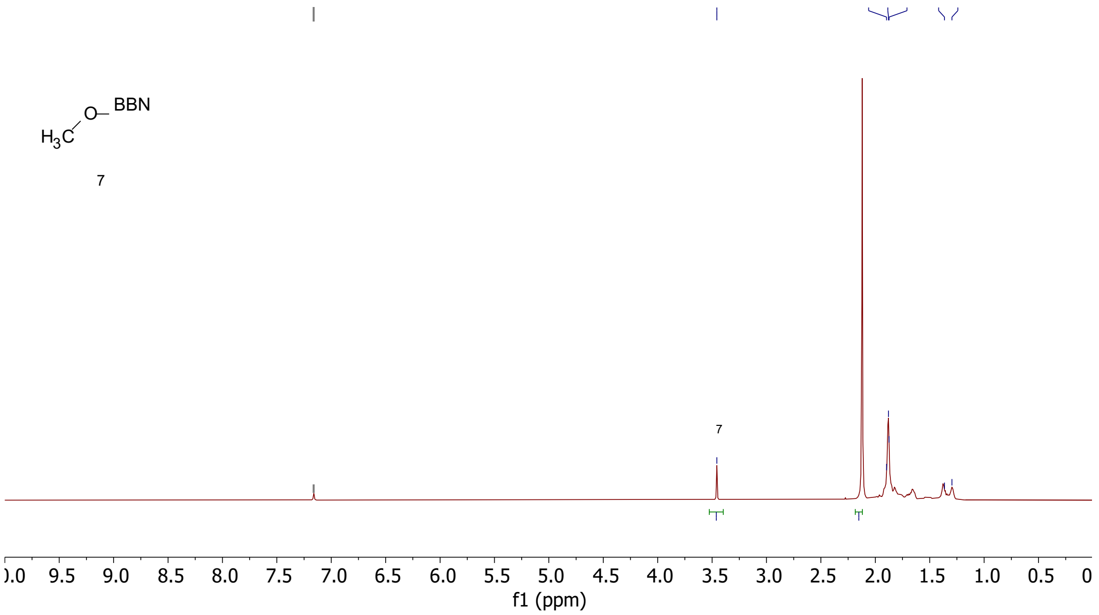

Figure S92: ^{1}H NMR spectrum (400 MHz, C_6D_6) entry 4 CO_2 reaction

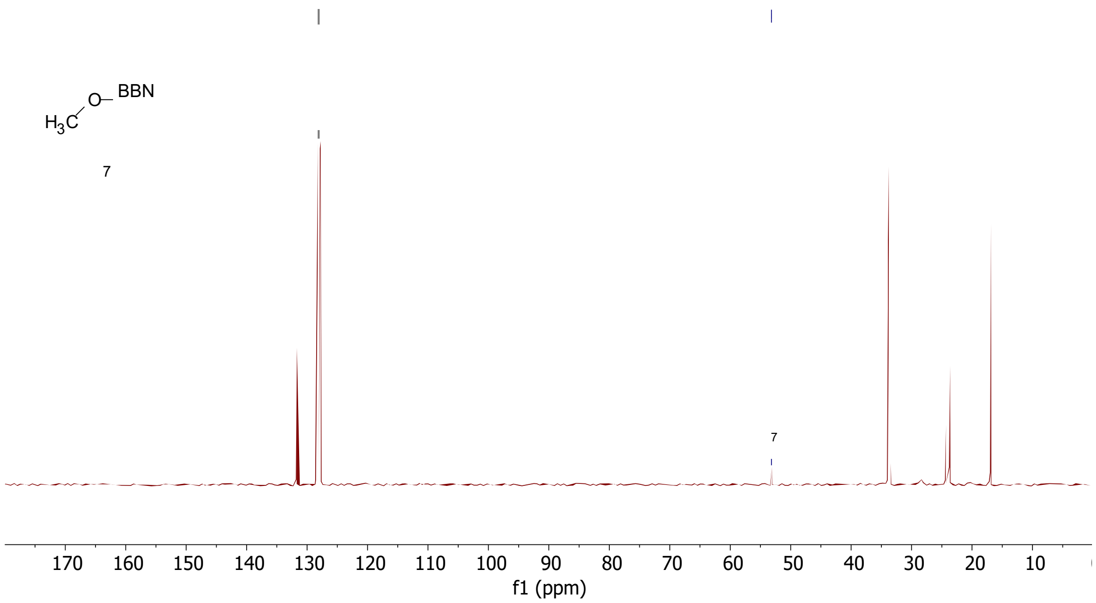

Figure S93: $^{13}C\{^1H\}$ NMR spectrum (101 MHz, C_6D_6) entry 4 CO_2 reaction

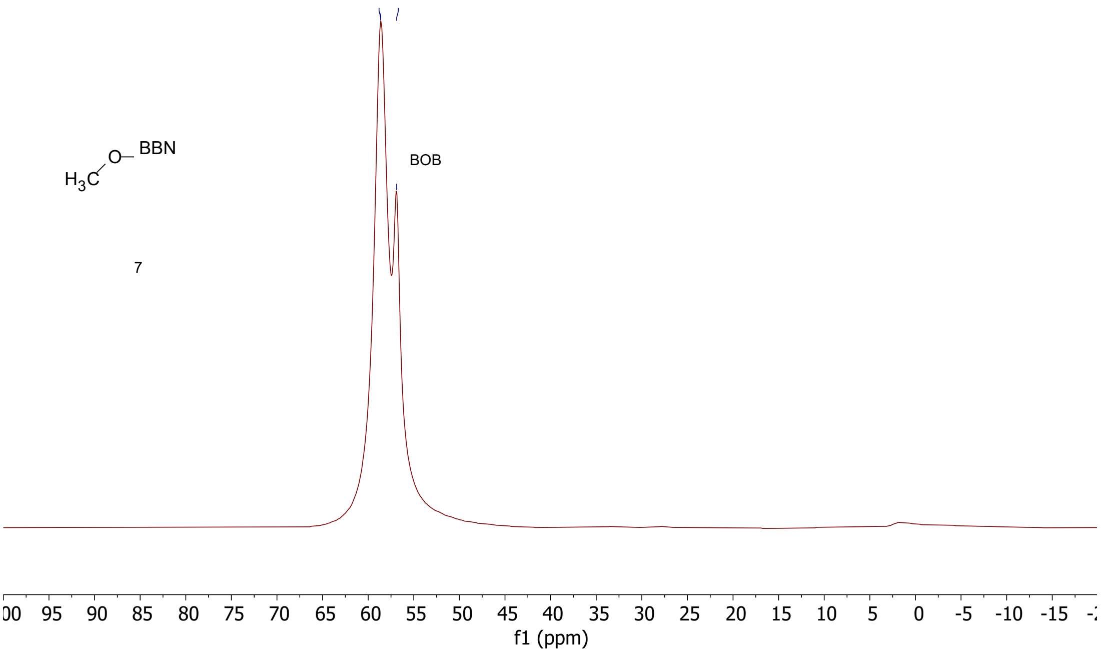

Figure S94: ^{11}B NMR spectrum (128 MHz, C_6D_6) entry 4 CO_2 reaction

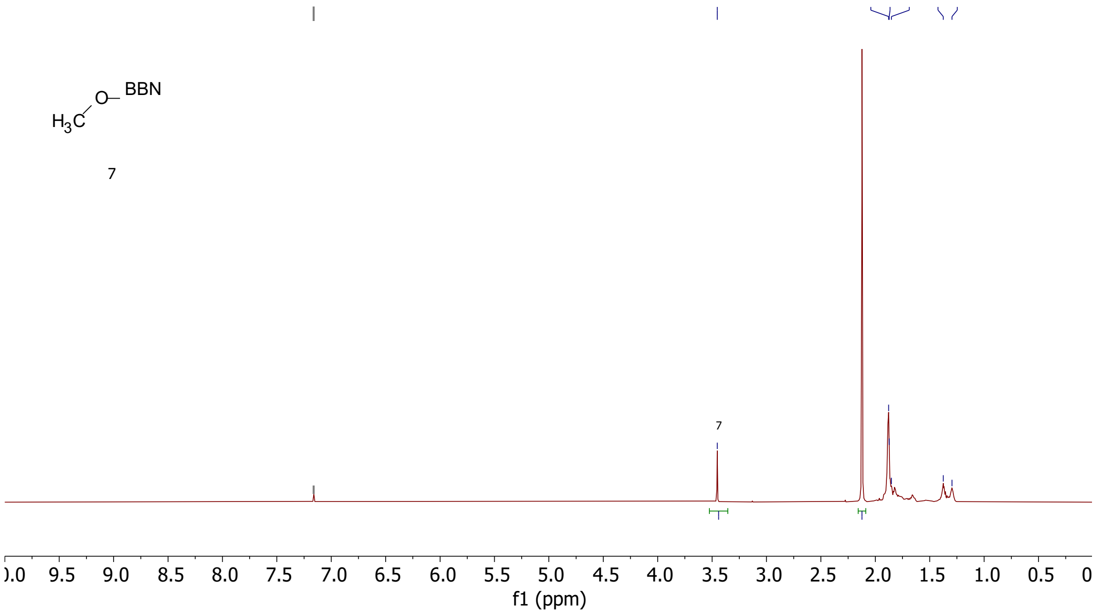

Figure S95: ^{1}H NMR spectrum (400 MHz, C_6D_6) entry 5 CO_2 reaction

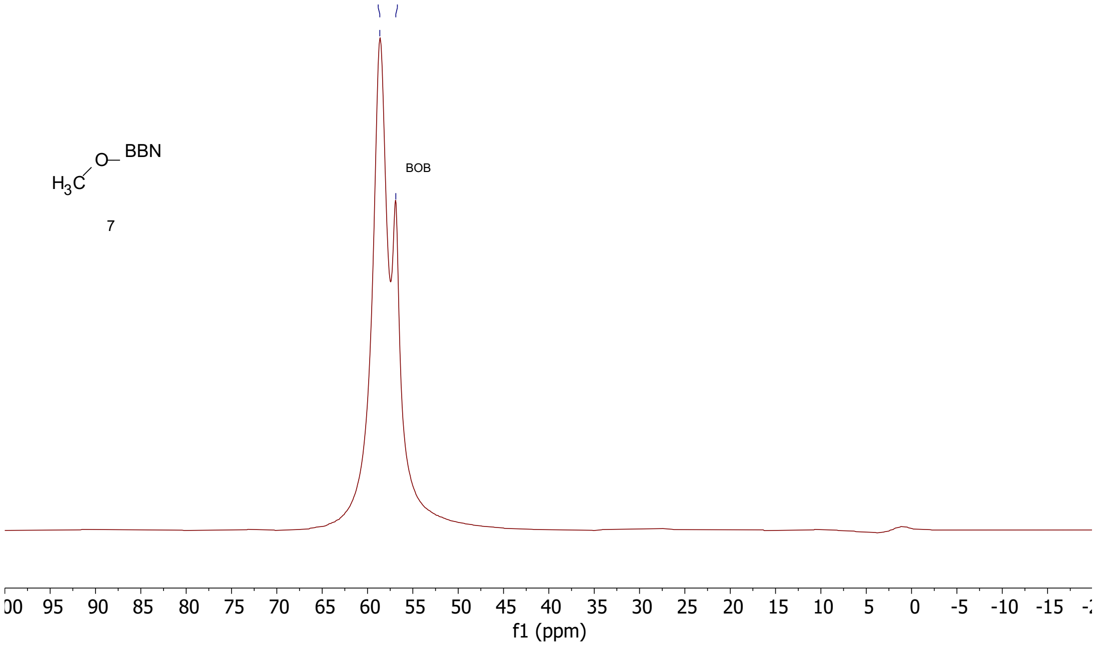

Figure S96: ^{11}B NMR spectrum (128 MHz, C_6D_6) entry 5 CO_2 reaction

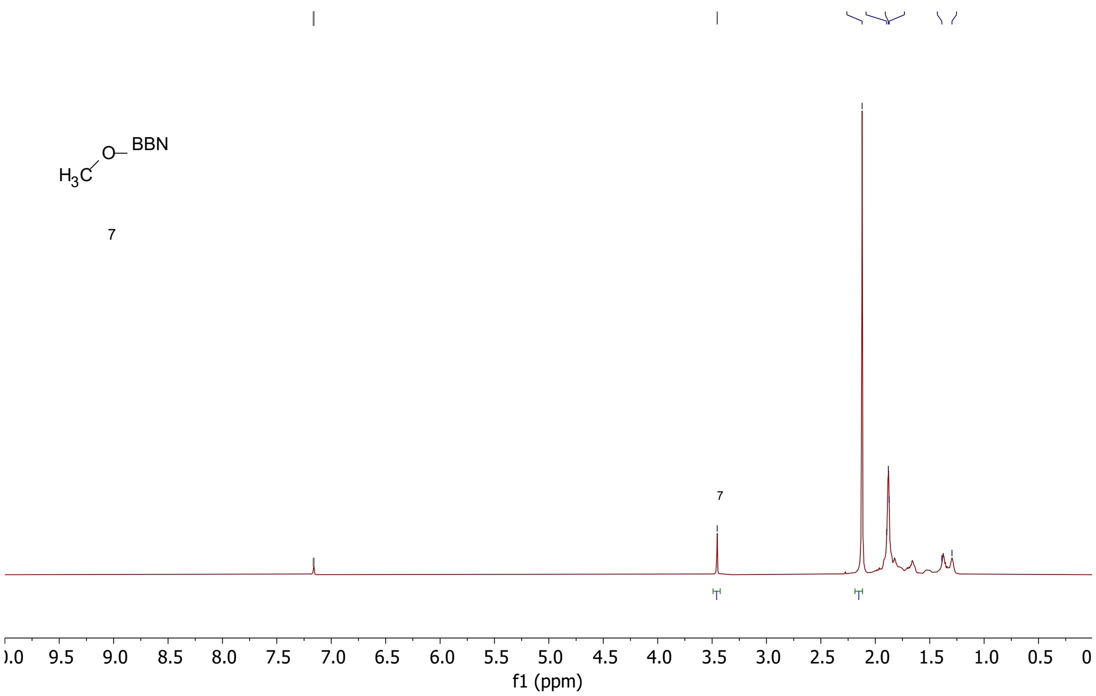

Figure S97: ^{1}H NMR spectrum (400 MHz, C_6D_6) entry 6 CO_2 reaction

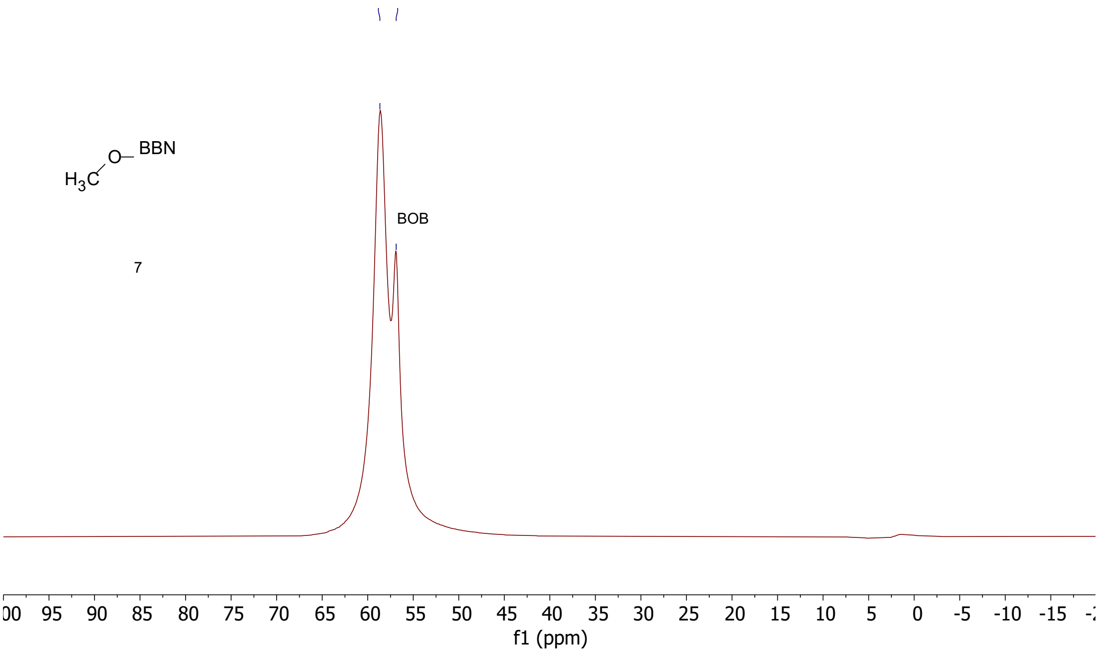

Figure S98: ^{11}B NMR spectrum (128 MHz, C_6D_6) entry 6 CO_2 reaction

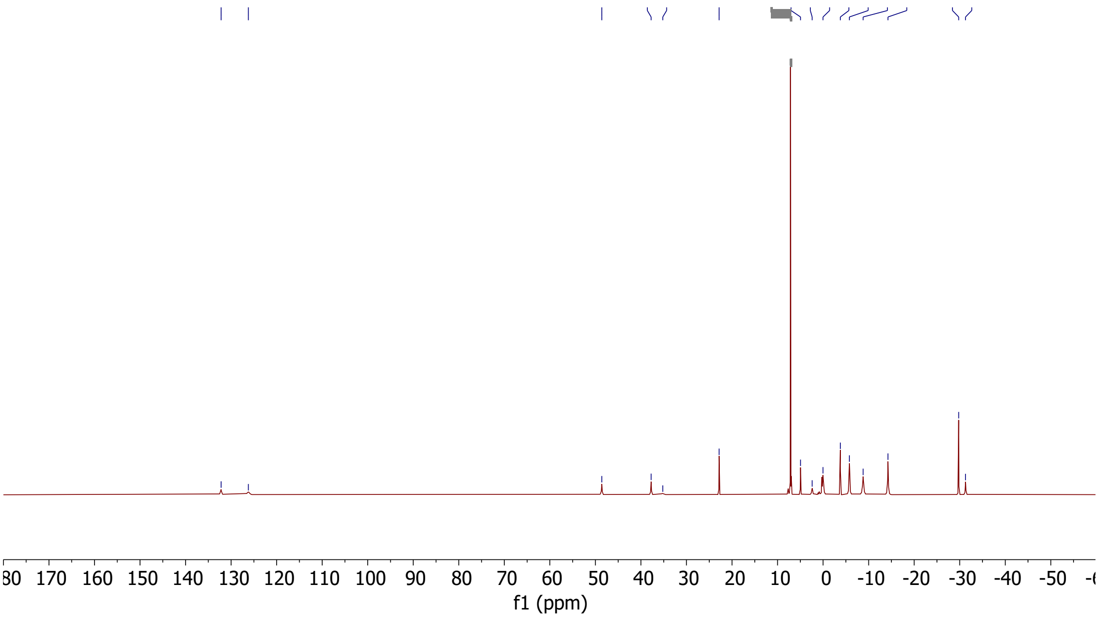

Figure S99: ^{1}H NMR spectrum (400 MHz, C_6D_6) of complex **9**

References

1. Bhawar, R., Patil, K. S. and Bose, S. K. CeO2–nanocubes as efficient and selective catalysts for the hydroboration of carbonyl groups. *New J. Chem.* **45**, 15028–15034 (2021).
2. Kumar, G. S., Harinath, A., Narvariya, R. and Panda, T. K. Homoleptic Zinc-Catalyzed Hydroboration of Aldehydes and Ketones in the Presence of HBpin. *Eur. J. Inorg. Chem.* **5**, 467–474 (2020).
3. Zhu, Z. *et al*. *n*-Butyllithium Catalyzed Selective Hydroboration of Aldehydes and Ketones. *J. Org. Chem.* **17**, 10677–10683 (2018).
4. R. Kumar, *et al*. Zirconium Complex as an Efficient Catalyst in the Hydroboration of Nitriles, Alkynes, and Carboxylic Esters: A Combined Experimental and Computational Study. *Organometallics*, **16**, 2216–2227 (2023).
5. Zheng, Y. *et al.* A bis-dianionic β-ketoiminato octalithium complex as a universal catalyst for hydroboration with broad scope. *New J. Chem.* **47**, 19367–19371 (2023).
6. *CrysAlisPro*, Agilent Technologies, Yarton, UK, 2017.
7. Bourhis, L. J. *et al*. The anatomy of a comprehensive constrained, restrained refinement program for the modern computing environment – Olex2 dissected. *Acta Cryst.* **A71**, 59–75 (2015).
8. Dolomanov, O. V. *et al.* OLEX2: a complete structure solution, refinement and analysis program. *J. Appl. Crystallogr.* **42**, 339–341 (2009).
9. Farrugia, L. J. WinGX and ORTEP for Windows: an update. *J. Appl. Crystallogr.* **45**, 849–854 (2012).
10. Espinai-Viguri, M., Neale, S. E., Coles, N. T., Macgregor, S. A. and Webster, R. L. Room Temperature Iron-Catalyzed Transfer Hydrogenation and Regioselective Deuteration of Carbon–Carbon Double Bonds. *J. Am. Chem. Soc.* **141**, 572–582 (2019).
11. Lin, Y.-S., Lin, G.-D., Mao, S.-P. and Chai, J.-D. Long-Range Corrected Hybrid Density Functionals with Improved Dispersion Corrections. *J. Chem. Theory Comput.* **9**, 263–272 (2013).
12. Weigend, F. and Ahlrichs, R. Balanced basis sets of split valence, triple zeta valence and quadruple zeta valence quality for H to Rn: Design and assessment of accuracy. *Phys. Chem. Chem. Phys.* **7**, 3297–3305 (2005).
13. Available at iqmol.org (accessed July 2025)
14. Glendening, E. D., Badenhoop, J. K., Reed, A. E., Carpenter, J. E., Bohmann, J. A., Morales, C. M. and Weinhold F. NBO 5.0. (Theoretical Chemistry Institute, University of Wisconsin, Madison, WI, 2001); http://www.chem.wisc.edu/~nbo5
15. Epifanovsky, E. *et al*. Software for the frontiers of quantum chemistry: An overview of developments in the Q-Chem 5 package. J. Chem. Phys. 155, 084801 (2021).
16. Soda T. *et al.* Ab initio computations of effective exchange integrals for H–H, H–He–H and Mn2O2 complex: comparison of broken-symmetry approaches. *Chem. Phys. Lett.* **319**, 223-230 (2000).

17. Gaussian 16, Revision A.03, Frisch, M. J. *et al*. Gaussian, Inc., Wallingford CT, 2016.
18. Grimme, S., Hansen, A., Ehlert, S. and Mewes, J. -M. r^2SCAN-3c: A "Swiss army knife" composite electronic-structure method. *J. Chem. Phys.* **154**, 064103 (2021).
19. (a) F. Neese, *WIRES Comput. Molec. Sci.* 2012, **2**, 73-78; (b) F. Neese, *WIRES Comput. Molec. Sci.* 2018, **8**, 1-6; (c) F. Neese, F. Wennmohs, U. Becker and C. Riplinger, *J. Chem. Phys.* **152**, 224108 (2020)
20. M. Garcia-Rates and F. Neese, *J. Comput. Chem.* **40**, 1816–1828 (2019)
21. M. Garcia-Rates and F. Neese, *J. Comput. Chem.* **41**, 922–939 (2020).
